# A Brain Region-Specific Predictive Gene Map for Autism Derived by Profiling a Reference Gene Set

**DOI:** 10.1371/journal.pone.0028431

**Published:** 2011-12-09

**Authors:** Ajay Kumar, Catherine Croft Swanwick, Nicole Johnson, Idan Menashe, Saumyendra N. Basu, Michael E. Bales, Sharmila Banerjee-Basu

**Affiliations:** 1 MindSpec, McLean, Virginia, United States of America; 2 NMBI Systems, New York, New York, United States of America; King's College London, United Kingdom

## Abstract

Molecular underpinnings of complex psychiatric disorders such as autism spectrum disorders (ASD) remain largely unresolved. Increasingly, structural variations in discrete chromosomal loci are implicated in ASD, expanding the search space for its disease etiology. We exploited the high genetic heterogeneity of ASD to derive a predictive map of candidate genes by an integrated bioinformatics approach. Using a reference set of 84 *Rare* and *Syndromic* candidate ASD genes (AutRef84), we built a composite reference profile based on both functional and expression analyses. First, we created a functional profile of AutRef84 by performing Gene Ontology (GO) enrichment analysis which encompassed three main areas: 1) neurogenesis/projection, 2) cell adhesion, and 3) ion channel activity. Second, we constructed an expression profile of AutRef84 by conducting DAVID analysis which found enrichment in brain regions critical for sensory information processing (olfactory bulb, occipital lobe), executive function (prefrontal cortex), and hormone secretion (pituitary). Disease specificity of this dual AutRef84 profile was demonstrated by comparative analysis with control, diabetes, and non-specific gene sets. We then screened the human genome with the dual AutRef84 profile to derive a set of 460 potential ASD candidate genes. Importantly, the power of our predictive gene map was demonstrated by capturing 18 existing ASD-associated genes which were not part of the AutRef84 input dataset. The remaining 442 genes are entirely novel putative ASD risk genes. Together, we used a composite ASD reference profile to generate a predictive map of novel ASD candidate genes which should be prioritized for future research.

## Introduction

Autism (MIM 209850) is a broad-spectrum multi-factorial condition which onsets in the first years of life and persists throughout the lifetime [1]. A triad of deficits in the areas of social communication, language development, and repetitive activities/restricted range of interests defines the core symptoms used in the diagnosis of autism (DSM IV, 1994). The affected areas show a broad range of variability in terms of both symptoms and severity; co-morbidity of epilepsy and mental retardation are often observed. Autism spectrum disorders (ASD) is a commonly used term to cover the wide variations of autism. The dramatic rise in the prevalence of ASD in recent years is of major public concern [2]–[3].

A strong genetic component underlying ASD has been firmly established from various lines of studies [4]–[7]. The search for ‘causative’ gene(s) has resulted in >10 whole genome scans reporting numerous putative linkage regions for ASD susceptibility [8]–[9]. Genetic association studies have identified numerous candidate genes for ASD [10]–[12]; however, most candidates fail to replicate between studies and populations. In a minor proportion of cases, chromosomal aberrations have been identified [13]. Recently, submicroscopic copy number variations (CNVs) were strongly associated with ASD [8], [14]–[15]. Additionally, ASD is consistently associated with a number of specific genetic disorders such as Fragile X Syndrome [16]–[17]. Single gene mutations are also linked to rare cases of ASD [18]–[19].

Together, hundreds of diverse genetic loci gathered from high throughput studies have been implicated in this disorder. Addressing the complexity of ASD, we have developed AutDB [20]–[21], a publicly available web-portal for on-going collection, manual curation, and visualization of genes linked to the disorder. First released by our laboratory in 2007, AutDB is widely used by both individual laboratories [22]–[25] and consortiums (Simons Foundation) [26] for understanding genetic bases of ASD.

Functional studies for isolated candidate genes have provided important insight into ASD but are largely restricted to rare monogenic forms of the disorder [27]–[28]. Here, we have exploited the genetic heterogeneity of ASD to create a predictive gene map for novel ASD candidate genes. To build this predictive map, we assembled a reference dataset of 84 ASD candidate genes from AutDB for dual profiling with both functional and expression analysis. We then used this dual profile to construct a predictive gene map for ASD which can be utilized in future research regarding pathogenesis of this complex psychiatric disorder.

## Results

### AutRef84 as an ASD Gene Reference Dataset

A reference dataset of ASD candidate genes was initially extracted from the autism gene database AutDB [20]–[21]. This resource provides systematic collection of candidate genes linked to ASD encompassing four genetic classifications 1) *Rare:* rare single gene variants, disruptions/mutations, and submicroscopic deletions/duplications directly linked to ASD, 2) *Syndromic*: genes implicated in syndromes in which a significant subpopulation develops autistic symptoms, 3) *Association*: small risk-conferring candidate genes with common polymorphisms identified from genetic association studies in idiopathic ASD, and 4) *Functional*: functional candidates. Genes belonging to more than one category are classified with both names.

In order to generate a high-confidence predictive gene map for ASD, we restricted the ASD reference gene set to higher risk-conferring, more penetrant ASD candidate genes. We filtered out lower risk-conferring ASD candidate genes, including *Functional* candidates devoid of any experimentally determined genetic link with ASD, as well as *Association* genes, which have suggestive evidence linking them to ASD [25], [29]–[30]. For instance, none of the genes in AutDB belonging solely to the *Association* category have reached genome-wide GWAS with independent replication or meta-analysis. The vast majority of candidate genes identified from genetic association studies are unreplicated or underpowered.

**Figure 1:**
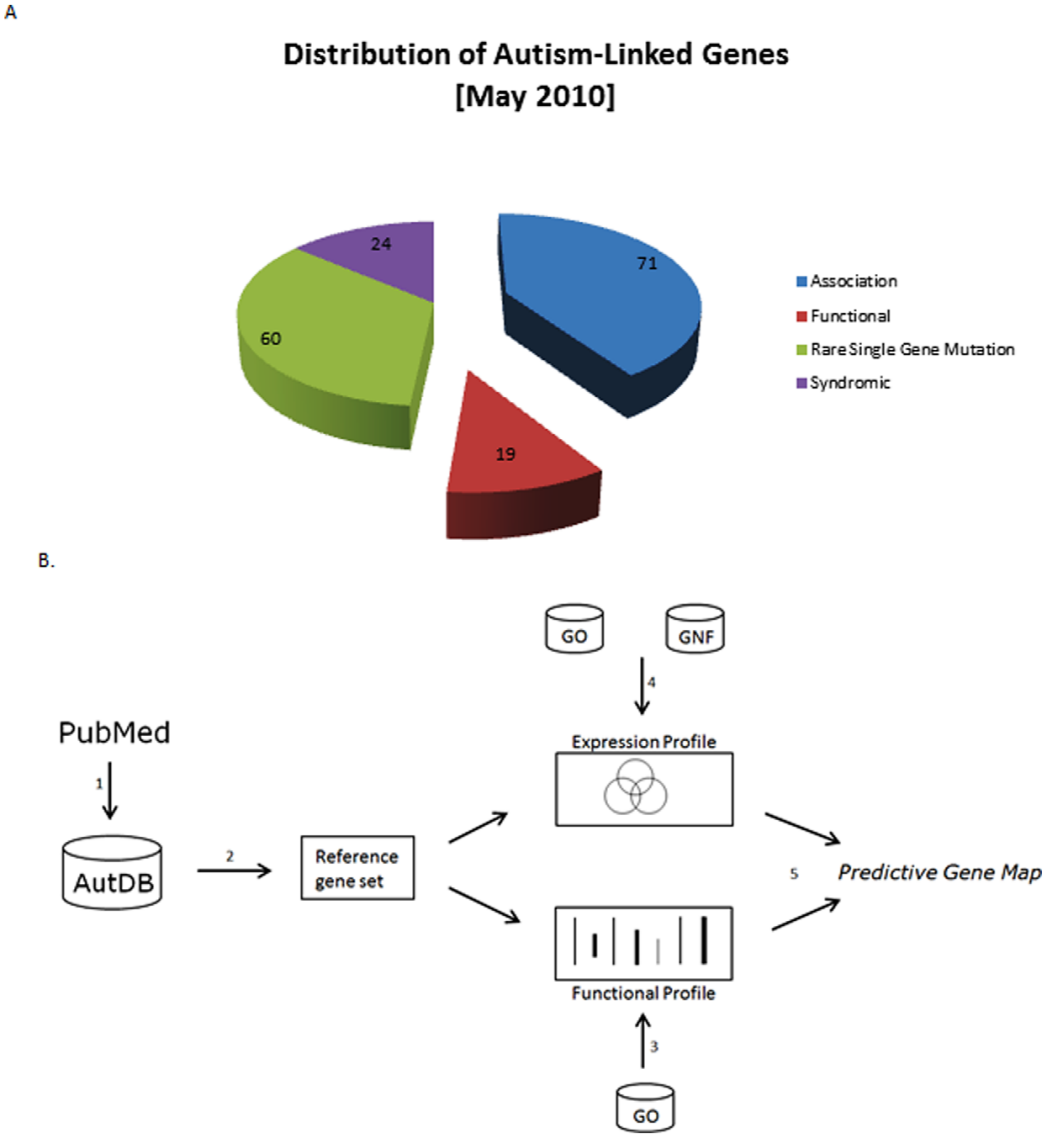
Integrated analysis of reference sets of genes Linked to ASD. A reference dataset of ASD-linked genes (AutRef84) was assembled from the *Rare* and *Syndromic* categories of AutDB (http://www.mindspec.org/autdb.html), a publicly available portal for ongoing collection of genes linked to ASD. **A**) Distribution of genetic categories in AutDB. **B**) Reference sets were analyzed using structured biological knowledge provided by Gene Ontology (GO) consortium [31].

The resulting ASD reference gene set, AutRef84, included 64 *Rare* and 20 *Syndromic* genes (**Figure 1A**), the total number of genes identified within these categories as of the data-freeze. The list of AutRef84 genes is presented in **Table 1**, whereas a more annotated version is provided as **Table S1**. The AutRef84 dataset encompasses well-studied candidates such as neuroligins (NLGN1, NLGN3, and NLGN4X), MECP2, FMR1, and TSC1/2, together with lesser-known genes with single reports including RPL10, CACNA1C, and DPP6. Hence, AutRef84 captures the broad landscape of ASD-linked genes suitable for applying statistical analysis to derive common gene functions.

**Table 1:** AutRef84: A Reference set of *Rare* and *Syndromic* ASD-linked genes.

Rare							Syndromic	
ANKRD11	CNTN4	FHIT	MBD4	PARK2	RFWD2	ST7	AHI1	DMD
AGAP1	CNTNAP2	GALNT13	MCPH1	PCDH10	RIMS3	SUCLG2	DHCR7	FMR1
APC	DLGAP2	GRPR	MDGA2	PCDH9	RPL10	TMEM195	DMPK	MECP2
ASTN2	DPP10	IL1RAPL1	NBEA	PLN	RPS6KA2	UBE3A	ADSL	NF1
AUTS2	DPP6	IMMP2L	NLGN1	PTCHD1	SCN1A		AGTR2	NTNG1
BZRAP1	DPYD	JMJD1C	NLGN3	RAB39B	SCN2A		ALDH5A1	PTEN
C3orf58	EIF4E	KCNMA1	NLGN4X	RAPGEF4	SEZ6L2		ARX	SLC6A8
CA6	FABP5	KIAA1586	NRXN1	RB1CC1	SHANK3		CACNA1C	TSC1
CACNA1H	FABP7	MBD1	ODF3L2	RBFOX1	SLC4A10		CACNA1F	TSC2
CADM1	FBXO40	MBD3	OR1C1	REEP3	SLC9A9		CDKL5	XPC

We then applied AutRef84 to generate a predictive gene map for ASD, as depicted by the schematic of our workflow presented in **Figure 1B**. In brief, we first performed a dual profile of AutRef84 consisting of both functional and expression analyses. We then screened the human genome with both branches of this profile in order to identify putative novel ASD candidate genes, as described below.

### Functional Profile: Common Biological Functions in AutRef84

To ascertain common biological functions associated with ASD-related genes, we adopted an integrated bioinformatics approach based on structured biological knowledge provided by Gene Ontology (GO) consortium [31]. We applied GO enrichment analysis to the AutRef84 dataset based on conditional Hypergeometric calculation of over-represented GO terms using Bioconductor packages [32]. Briefly, all three branches of GO knowledge structure (biological process (BP), molecular function (MF), and cellular component (CC)) were utilized for this analysis. To test for GO category enrichment, we performed the conditional Hypergeometric function of Bioconductor using a set of stringent filter criteria: 1) *P*-value cutoff of 0.001, 2) limited GO annotation category size of 100≤x≤1000 to minimize artificial elevation of *P*-value, and 3) gene count of >4 in significant categories. Applying these filters, a total of 15 enriched GO categories were identified in AutRef84: 10 BP categories, three MF categories, and two CC categories (**Table 2**). Examples of enriched categories with highest gene content per GO branch include *cell adhesion* (BP: 13 genes; *P* = 1.1×10^−4^), *cation transport* (BP: 10 genes, *P* = 7.9×10^−4^), *neurogenesis* (BP: 10 genes, *P* = 4.0×10^−5^), *voltage-gated channel activity* (MF: 6 genes; *P* = 3.4×10^−4^), and *synapse* (CC; 7 genes; *P* = 6.2×10^−4^).

**Table 2:** Functional Profile of AutRef84: Enriched Gene Ontology (GO) Categories.^1^

		GO ID	GO Term	P- Value	Odds Ratio	Exp Count	AutRef84 Genes	Size
**ASD**	**BP**	GO:0007417	Central Nervous System Development	4.00E-05	6.235	1.656	9	303
		GO:0022008	Neurogenesis	4.00E-05	5.485	2.104	10	385
		GO:0030182	Neuron Differentiation	6.00E-05	5.847	1.760	9	322
		GO:0007155	Cell Adhesion	1.10E-04	3.907	3.902	13	714
		GO:0009968	Negative Regulation of Signal Transduction	3.30E-04	7.180	0.929	6	170
		GO:0031175	Neuron Projection Development	4.80E-04	6.646	1.000	6	183
		GO:0044057	Regulation of System Process	6.00E-04	6.355	1.044	6	191
		GO:0019220	Regulation of Phosphate Metabolic Process	9.70E-04	4.344	2.049	8	375
		GO:0006814	Sodium Ion Transport	6.90E-04	7.818	0.705	5	129
		GO:0006812	Cation Transport	7.90E-04	3.740	3.012	10	536
	**MF**	GO:0022843	Voltage-Gated Cation Channel Activity	7.00E-05	9.662	0.699	6	135
		GO:0031402	Sodium Ion Binding	2.60E-04	9.760	0.570	5	110
		GO:0022832	Voltage-Gated Channel Activity	3.40E-04	7.098	0.938	6	181
	**CC**	GO:0034703	Cation Channel Complex	6.00E-05	9.980	0.675	6	125
		GO:0045202	Synapse	6.20E-04	5.309	1.454	7	269
**Diabetes**	**BP**	GO:0065008	Regulation of Biological Quality	2.49E-09	9.553	2.772	16	948
		GO:0003013	Circulatory System Process	2.67E-09	17.334	0.747	10	194
		GO:0009893	Positive Regulation of Metabolic Process	6.80E-08	7.801	2.450	14	662
		GO:0016265	Death	8.95E-04	3.552	3.706	11	963
		GO:0012501	Programmed Cell Death	1.55E-03	3.503	3.356	10	872
	**MF**	GO:0005179	Hormone Activity	6.26E-03	8.644	0.376	3	101
		GO:0019900	Kinase Binding	9.82E-03	7.293	0.443	3	119
	**CC**	GO:0000267	Cell Fraction	2.00E-03	3.608	2.881	9	805
		GO:0031090	Organelle Membrane	4.62E-03	3.419	2.671	8	787
**Control** *	**BP**	GO:0032940	Secretion By Cell	2.36E-05	4.921	2.856	12	524
		GO:0010647	Positive Regulation Of Cell Communication	4.50E-05	4.964	2.570	11	478
		GO:0023056	Positive Regulation Of Signaling	4.76E-05	4.931	2.586	11	481
		GO:0010740	Positive Regulation Of Intracellular Protein Kinase Cascade	6.29E-05	6.644	1.365	8	254
		GO:0018130	Heterocycle Biosynthetic Process	8.27E-06	6.727	1.741	10	343
		GO:0034654	Nucleobase, Nucleoside, Nucleotide And Nucleic Acid Biosynthetic Process	1.84E-05	6.881	1.513	9	298
		GO:0044283	Small Molecule Biosynthetic Process	6.05E-05	4.467	3.158	12	622
		GO:0009165	Nucleotide Biosynthetic Process	7.63E-05	6.472	1.406	8	277
		GO:0051090	Regulation Of Transcription Factor Activity	2.94E-05	7.432	1.226	8	225
		GO:0051098	Regulation Of Binding	3.30E-05	6.341	1.624	9	298
		GO:0043254	Regulation Of Protein Complex Assembly	2.69E-05	11.554	0.591	6	110
		GO:0031329	Regulation Of Cellular Catabolic Process	7.47E-05	6.488	1.401	8	272
		GO:0009141	Nucleoside Triphosphate Metabolic Process	8.57E-06	6.020	2.153	11	418
		GO:0009205	Purine Ribonucleoside Triphosphate Metabolic Process	4.20E-05	5.505	2.102	10	408
		GO:0009259	Ribonucleotide Metabolic Process	8.22E-05	5.058	2.277	10	442
		GO:0071844	Cellular Component Assembly At Cellular Level	5.31E-05	4.004	4.165	14	797
		GO:0051336	Regulation Of Hydrolase Activity	6.90E-05	4.716	2.692	11	494
		GO:0006259	DNA Metabolic Process	1.42E-05	4.856	3.199	13	621

BP = Biological Process, MF = Molecular Function, CC = Cellular Component.

* = 1000 gene sets of n = 84, randomly assembled from OMIM.

For additional support, we also performed GO enrichment analysis using the DAVID bioinformatics resource, which employs a Fisher's Exact Test instead of a conditional Hypergeometric test. With a *P*-value cut-off of p<0.05, we derived a total of 26 enriched GO categories using DAVID analysis: 21 BP categories and five CC categories (**Table S2**). Whereas all categories from both analyses related to similar themes, the two CC categories derived from Bioconductor matched exactly with those generated from DAVID, as did four of the BP categories.

We further characterized functionality of the AutRef84 gene set by conducting pathway analysis with Pathway Express [33]. Using the KEGG database, we derived five significantly enriched molecular pathways: *cell adhesion molecules* (6 genes, *P* = 7.5×10^−6^), *mTOR signaling pathway* (4 genes, *P* = 3.3×10^−5^), *calcium signaling pathway* (5 genes, *P* = 4.4×10^−4^), *p53 signaling pathway* (3 genes, *P* = 1.8×10^−3^), and *MAPK signaling pathway* (5 genes, *P* = 2.6×10^−3^) (**Table S3**). One of these pathways, *cell adhesion molecules*, is shared exactly with the GO enrichment analysis. The other categories are shared indirectly: *calcium signaling pathway* is included in the GO enrichment categories *cation transport* and *cation channel activity*, while the *mTOR*, *p53*, and *MAPK signaling pathways* relate to the GO enrichment category *negative regulation of signaling transduction*.

**Figure 2:**
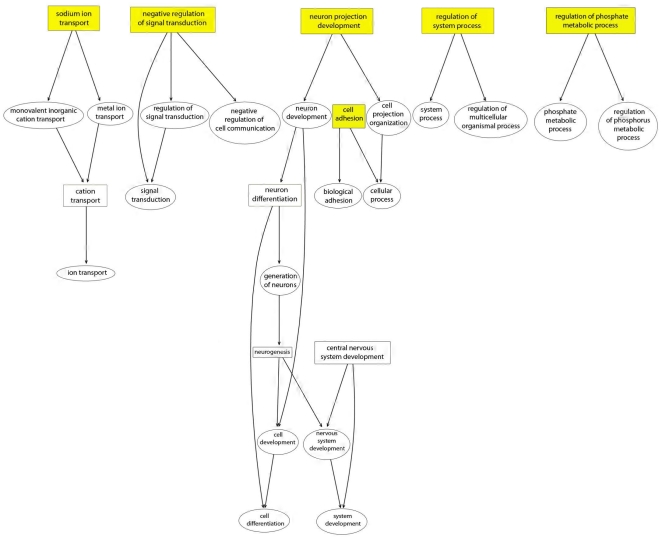
AutRef84 functional profile: graphical representation of over-represented Biological Process (BP) categories. Using Bioconductor, we generated directed acyclic graphs based on GO knowledge structure. Enriched GO categories of AutRef84 are represented by rectangular boxes. Terminal nodes are illustrated in yellow. The largest structural component of the BP GO Tree is connected to *neuron projection development*, which includes the enriched GO categories of *neuron differentiation* (9 genes, P = 6.0×10^−5^), *neurogenesis* (10 genes, 4.0×10^−5^), and *central nervous system development* (9 genes, P = 4.0×10^−5^). Other enriched terminal nodes relate to ion channel activity or cell adhesion.

To visualize the relationship among enriched GO categories, we generated directed acyclic graphs based on GO knowledge structure. The terminal nodes of these GO Trees, devoid of any ascendants provide a framework for semantics of candidate gene functions in AutRef84. Within the BP graph (**Figure 2**), enriched terminal nodes relate to ion channel activity (*sodium ion transport*: 5 genes, P = 6.9×10^−4^; *negative regulation of signal transduction*: 6 genes, P = 3.3×10^−4^; *regulation of system process*: 6 genes, P = 6.0×10^−4^; *regulation of phosphate metabolic process*: 8 genes, P = 9.7×10^−4^), neurogenesis/projection (*neuron projection development*: 6 genes, P = 4.8×10^−4^), or cell adhesion (*cell adhesion:* 11 genes; P = 8.4×10^−4^). Notably, the largest structural component of the BP GO Tree is connected to *neuron projection development*, which includes the enriched GO categories of *neuron differentiation* (9 genes, P = 6.0×10^−5^), *neurogenesis* (10 genes, 4.0×10^−5^), and *central nervous system development* (9 genes, P = 4.0×10^−5^). Within the AutRef84 MF graph (**Figure S1**), enriched terminal nodes also relate to ion channel activity (*voltage-gated cation channel activity*: 6 genes, P = 7.0×10^−5^; *sodium ion binding*: 5 genes, P = 2.6×10^−4^). Similarly, within the AutRef84 CC graph (**Figure S2**), enriched terminal nodes describe cellular components important for ion channel activity (*cation channel complex*: 6 genes, P = 6.0×10^−5^) or ion channel activity/cell adhesion (*synapse*: 7 genes, P = 6.2×10^−4^).

### Expression Profile: Region-Specific Enrichment of AutRef84

**Figure 3:**
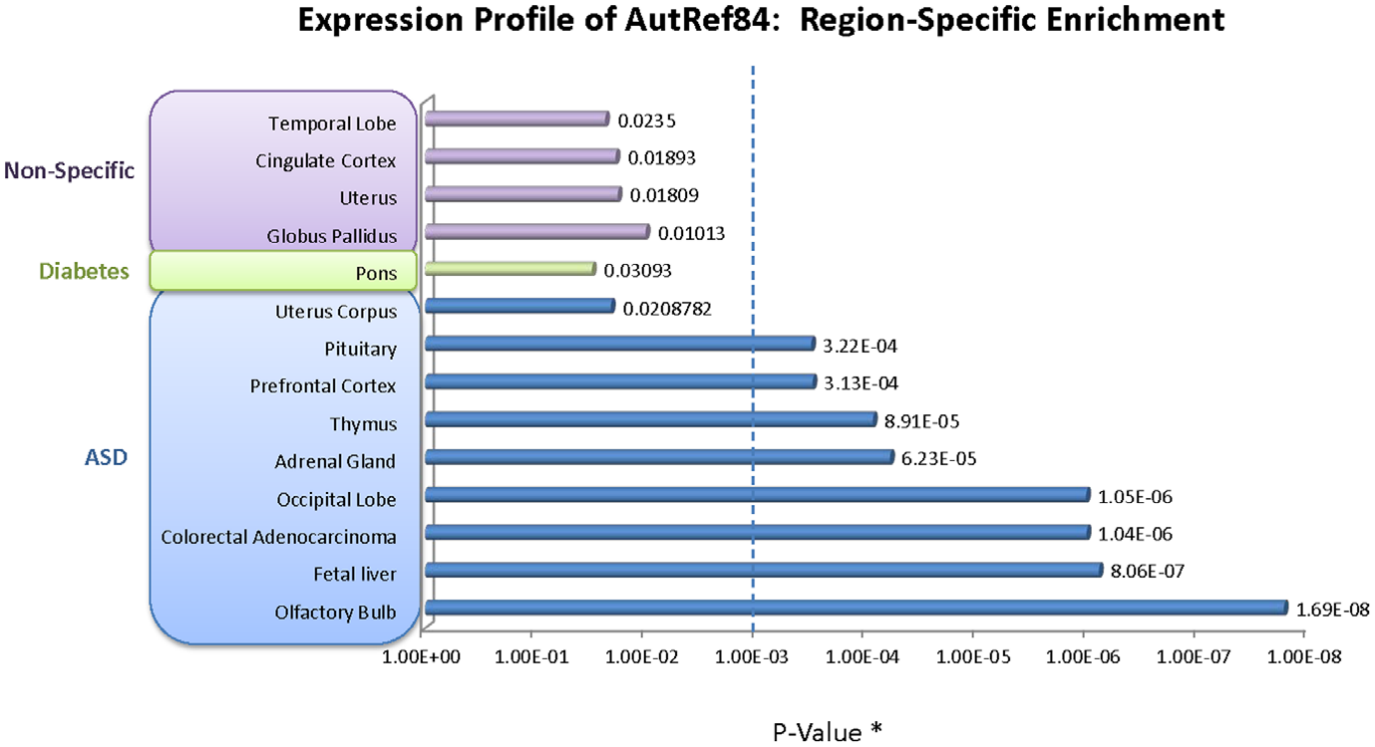
AutRef84 expression profile: region-specific enrichment of gene expression. Analysis of tissue expression profiles for AutRef84 genes using the DAVID bioinformatics tool (http://david.abcc.ncifcrf.gov/) demonstrates region-specific enrichment with high statistical significance (p<0.0001) in four areas of the central nervous system: olfactory bulb, occipital lobe, prefrontal cortex, and pituitary. Whereas the olfactory bulb and occipital lobe are involved in sensory processing (smell and vision, respectively), the prefrontal cortex controls executive function and the pituitary gland directs hormone secretion. None of the enriched regions overlapped with those of diabetes or non-specific disease gene sets. * = Bonferroni corrected.

We next performed tissue-specific expression analysis of AutRef84 using the DAVID bioinformatics resource. We discovered nine anatomical regions of highly enriched ASD candidate gene expression (P≤1.0×10^−4^; accounting for multiple testing), including four brain regions: 1) the olfactory bulb (33 genes, P = 1.7×10^−8^), which transmits smell; 2) the occipital lobe (32 genes, P = 1.0×10^−6^), which processes visual information; 3) the prefrontal cortex (25 genes, P = 3.1×10^−4^), which is important for executive function; and 4) the pituitary (26 genes, P = 3.2×10^−4^), which regulates hormone secretion (**Figure 3**). The enrichment of ASD-linked genes within non-brain regions is not surprising, given the pleiotropic expression of genes within the human body. However, due to the categorization of ASD as a neuropsychiatric illness, we focused exclusively on enriched brain regions. A list of AutRef84 genes expressed within each region is provided in **Table S4**.

**Figure 4:**
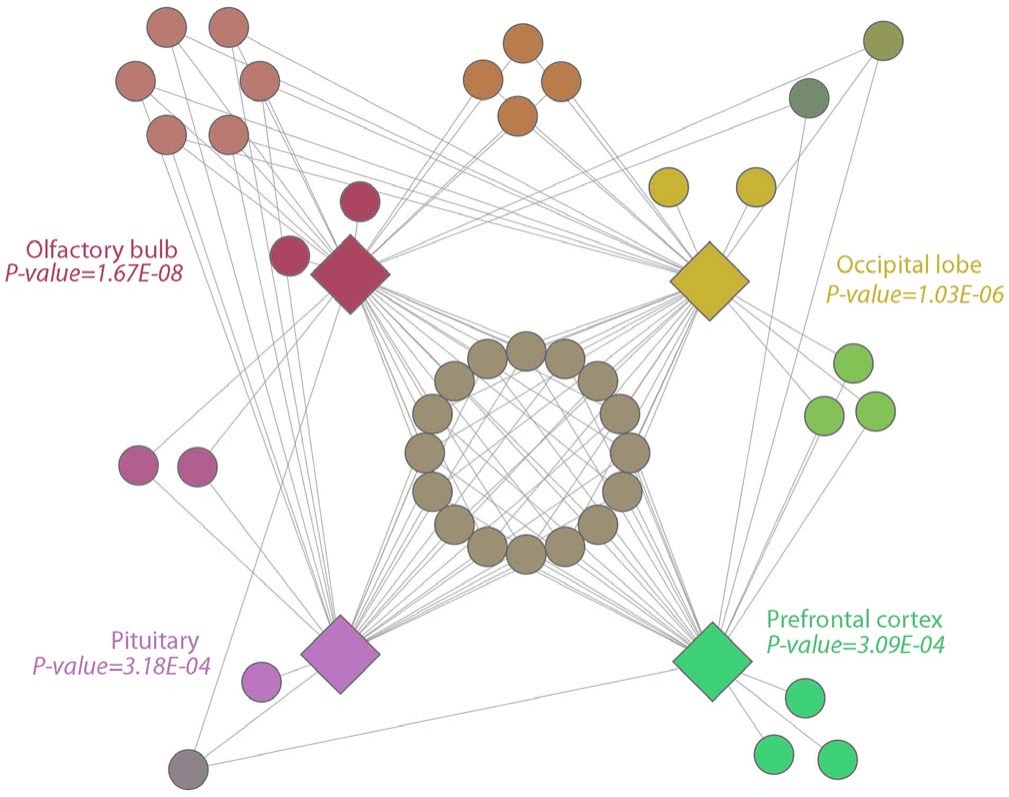
Network analysis of the AutRef84 expression profile. In this visual representation of the network, each group of gene nodes is spatially positioned near the brain region or regions in which the genes are expressed. The color of each group of gene nodes was derived by averaging red, green, and blue values of the colors of the linked brain region nodes.

We then performed network representation to illustrate relationships of AutRef84 gene expression among these shared brain regions (**Figure 4**). Of the AutRef84 gene set, a total of 45 genes were enriched among these four brain regions. A core set of 16 genes showed enriched expression in all four brain regions: A2BP1, APC, CNTNAP2, DPP6, FABP7, IL1RAPL1, NBEA, NF1, NLGN3, NLGN4X, NRXN1, PCDH9, RAPGEF4, RIMS3, SCN2A, and SEZ6L2. Additionally, eight genes were expressed in three regions (including the Rett Syndrome gene MECP2 and the Fragile X Syndrome gene FMR1), ten genes were expressed in two regions, and eight genes were expressed in only one of these enriched regions.

### Disease Specificity of AutRef84 Dual Profile

To examine disease specificity of the AutRef84 dual profile, we compared it to both 1) a diabetes set of 54 genes serving as a disease-specific control (**Table S5**), 2) a non-specific disease reference set of 78 genes generated by randomly sampling genes the OMIM database which did not show significant association to any one particular disease, and 3) 1000 control gene sets of size n = 84 that were randomly sampled from the OMIM database.

First, we analyzed AutRef84 functional profile specificity. By performing GO enrichment analysis with the same filtering criteria as above, we found that the diabetes gene set was enriched within nine GO categories (five BP categories, two MF categories, and two CC categories) (**Table 2**), and the 1000 random control gene sets were enriched within 18 GO categories (all BP categories) (**Table 2**). None of the top enriched GO terms were shared among ASD, diabetes, and control datasets. Whereas the top enriched GO terms for ASD were concentrated in synaptic functions, the diabetes reference set was enriched for metabolic cellular processes, and the control reference sets were distributed primarily across various signaling pathways.

Second, we examined AutRef84 expression profile specificity. For this analysis, we used compared AutRef84 with the diabetes gene set as well as a non-specific disease gene set assembled by randomly sampling OMIM (**Table S6**). Region-specific enrichment of the diabetes gene set was restricted to the pons, whereas expression of the non-specific disease dataset was concentrated in the uterus and three brain regions distinct from that of ASD (**Figure 3**). Like the functional profile, no enrichment was shared among ASD, diabetes, and non-specific disease datasets. Although each gene set showed enrichment in distinct brain regions, it is noteworthy that statistical significance of the enriched ASD brain regions was generally two orders of magnitude higher than those of diabetes and non-specific disease datasets. Eight of the nine ASD regions satisfy a more stringent *P*-value of 0.001 (**Figure 3**, dotted line), whereas none of the diabetes and control regions meet this cut-off, implying that brain region enrichment of ASD genes is much less likely to occur by chance.

### Predictive Gene Map Generated with Dual AutRef84 Profile

**Figure 5:**
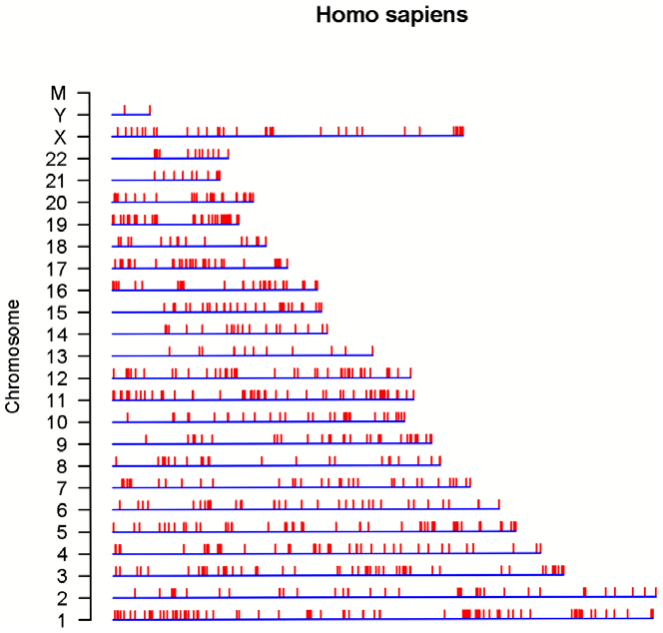
Genome-wide screening with the functional AutRef84 profile. The functional profile of AutRef84 was used to predict ASD genes and map them to their appropriate location on the chromosome. To perform this data mining, we used the biomaRT package of Bioconductor from human genome at the Ensembl database (http://www.ensembl.org/Homo_sapiens) to create a graphical representation of chromosomal locations of genes matching with the functional AutRef84 profile. The complete list of 1185 matching genes is provided as **Table S7**. This map indicates uneven distribution with dense packing of matched genes in discrete chromosomal regions, 13 of which reached statistical significance (**Table S8**).

The creation of AutRef84 provides a usable framework for systems biology analysis of molecular events underlying ASD pathogenesis. Here we applied the dual AutRef84 profile to generate a predictive gene map for ASD. First we used the functional AutRef84 profile to screen the human genomic sequence at Ensembl database (NCBI build 36) with the biomaRt package of Bioconductor. We identified 1185 genes matching the functional AutRef84 profile (**Table S7**), and their genome-wide distribution pattern is illustrated in **Figure 5**. This map indicates uneven distribution, with dense packing of matched genes in 13 discrete chromosomal regions that reached statistical significance using a DAVID analysis with a *P*-value cut-off value of p<0.05 (**Table S8**).

**Figure 6:**
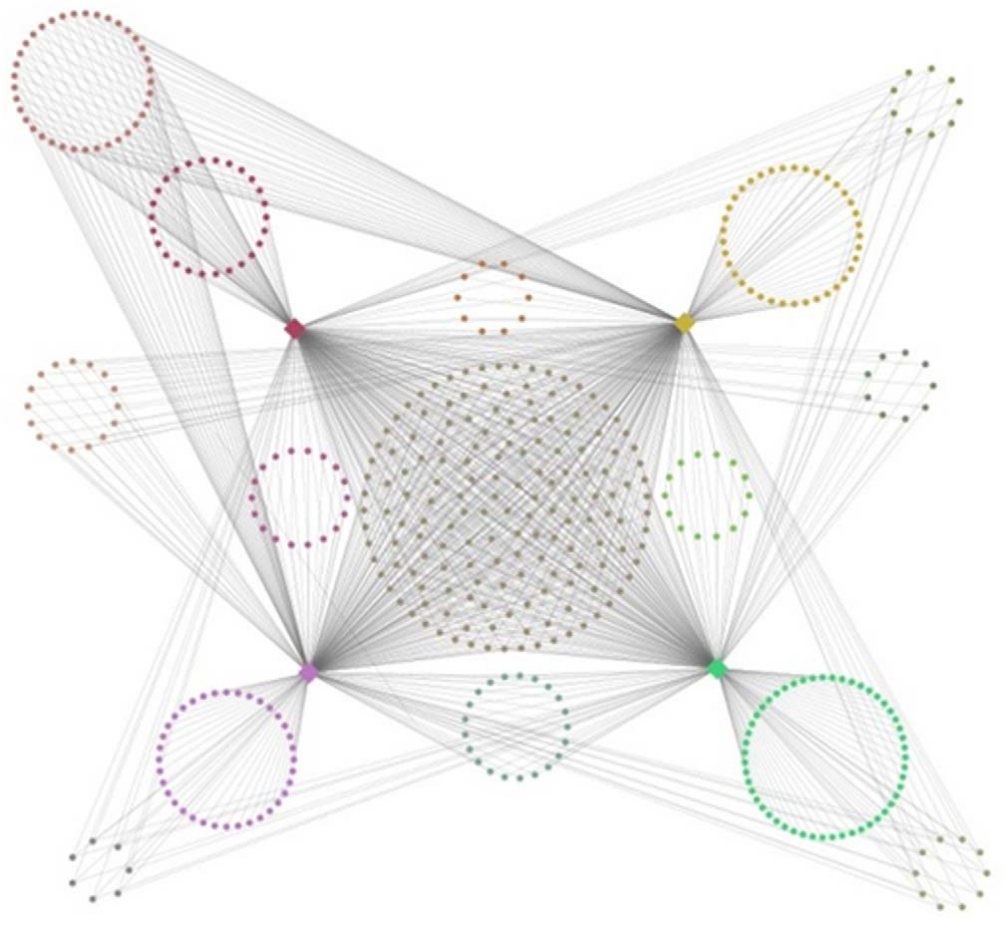
Network representation of ASD predictive gene map matching the dual profile of AutRef84. After initially identifying 1185 genes matching the AutRef84 functional profile, we filtered this set by performing tissue-specific enrichment analysis and network representation of its shared brain regions within the AutRef84 expression profile. Using this method of dual profiling, we defined a prioritized subset of 460 genes predicted to be mutated in individuals with ASD. Within this subset, 159 genes are expressed in all four enriched brain regions of AutRef84, 62 genes were common to three of the enriched brain regions, 89 genes overlapped in two of the enriched regions, and 150 genes were expressed in only one enriched brain region (**Table S9**). Node placement and coloring were determined as described in **Figure 4**.

We then applied the AutRef84 expression profile to filter this initial predictive gene set. Using network representation of shared brain expression within the set of 1185 genes described above, we defined a subset of 460 genes matching both functional and expression profiles of AutRef84 (**Figure 6**). Of this subset, 159 genes are expressed in all four enriched brain regions, 62 genes were common to three of the enriched brain regions, 89 genes overlapped in two of the enriched regions, and 150 genes were expressed in only one of the enriched brain regions (**Table S9**).

Importantly, the accuracy of the final predictive gene map was demonstrated by correctly capturing 18 existing ASD-associated genes which were not part of the AutRef84 input dataset (**Table S10**): 13 genes linked to ASD by genetic association studies (therefore part of the *Association* category of AutDB), three genes whose function is relevant to ASD (therefore included in the *Functional* category of AutDB), and two *Rare*/*Syndromic* genes that have been discovered since the original AutRef84 data-freeze. Some of these candidate genes are particularly interesting candidates for ASD, such as GABRB3, a GABA receptor subunit linked to ASD by multiple association studies [34]–[41], NPAS2, a transcription factor involved in circadian rhythms that has been associated with ASD [42]; RELN, an extracellular matrix protein involved in cell migration whose association with ASD has been replicated [43]–[46]; and SEMA5A, an axon guidance molecule shown to be downregulated in ASD [47]. However, the remaining 442 genes have not previously been associated with ASD and form a novel pool of potential ASD candidate genes.

## Discussion

In this report, we have defined the first composite reference profile of ASD candidate genes, AutRef84. In contrast to another recent ASD profile based on functional annotation of candidate genes [48], here we created a dual reference profile of AutRef84 by performing both functional and expression analyses of ASD candidate genes. Derived from data extracted from >158 references, AutRef84 consolidates knowledge about *Rare* and *Syndromic* ASD genes, whose relationship to ASD has been firmly established. For the functional profile, we conducted GO enrichment to discover that AutRef84 genes are enriched in biological functions related to three major areas: 1) neurogenesis/projection, 2) cell adhesion, and 3) ion channel activity. For the expression profile, we analyzed tissue-specific expression patterns to find that AutRef84 genes are enriched in brain regions vital to sensory information processing (olfactory bulb, occipital lobe), executive function (prefrontal cortex), and hormone secretion (pituitary). We then applied this dual profile to create a genome-wide predictive gene map for ASD consisting of 460 putative candidate genes. Of these 460 genes, 18 were previously associated with ASD but were included in our input AutRef84 dataset, demonstrating the predictive power of this gene map. The remaining 442 genes are entirely novel putative ASD risk genes. Together, our predictive gene map can serve as a tool for researchers to prioritize molecular pathways underlying ASD pathogenesis, thereby accelerating the discovery of targeted treatments for this disorder.

Our functional profile revealed that ASD candidate genes are concentrated in three biological processes critical for synaptic transmission: neurogenesis/projection, cell adhesion, and ion channel activity. A ‘synaptic dysfunction’ hypothesis for ASD is widely acknowledged [49]–[50]. However, molecular support for this hypothesis rests mainly on cell adhesion binding partners neuroligins (NLGN3, NLGN4X) and neurexins (NRXN1), as well as the scaffolding protein SHANK3 – all identified in rare cases of ASD. The availability of curated, annotated datasets of ASD-linked genes provides unique computational opportunities to identify common biological functions associated with these genes [25]. Here, we use a reference set of genes, rigorous statistical analysis, and comparative analysis with multiple control datasets to provide molecular support for synaptic bases of ASD.

For instance, the largest concentration of synaptic categories for ASD-linked genes involves ion regulation. Six of the 15 enriched GO categories for AutRef84 were *sodium transport*, *cation transport*, *voltage-gated cation channel*, *sodium ion binding*, *voltage-gated channel activity*, and *cation channel complex*. This unbiased study of ASD candidate genes supports a previously established theory of ASD pathogenesis proposing an increased excitation∶inhibition ratio [51]. In correspondence with this theory, approximately 10–30% of individuals with autism are also diagnosed with epilepsy [52]–[53], a disease caused by ion channel dysfunction. To further examine the role of ion channels in both diseases, future studies should compare the functional profile of AutRef84 with one created from an epilepsy reference gene set.

Another major component of our ASD reference profile is neurogenesis/projection. Enriched GO categories within this neurobiological classification included *neurogenesis*, *neuron differentiation*, *neuron projection development*, *central nervous system development*, and *cell adhesion*. Impairments of these neurodevelopmental processes may contribute to accelerated head growth observed in children with ASD [54]–[55]. Additionally, neurogenesis continues to play a role in adult function of brain regions such as the hippocampus [56] and amygdala [57]. Inability of neurons to regenerate within these brain areas may lead to deficits in emotional processing observed in autism [58]–[59].

Our expression profile defines four critical brain regions of ASD pathogenesis: olfactory bulb, occipital lobe, prefrontal cortex, and pituitary. Dysfunction of each of these brain areas in ASD has been suggested by previous functional evidence. For example, he olfactory bulb, which transmits information pertaining to smell, has been strongly implicated in mouse models of ASD due to its well-established role in their social behavior [60]. Interestingly, humans with ASD also exhibit altered olfactory perception [61]–[62]. Electrical abnormalities have been observed in the occipital lobe of ASD individuals [63], suggesting that impaired facial recognition associated with ASD [64] may at least partially be due to altered visual processing. The prefrontal cortex is critical for executive function skills deficient in ASD, such as decision-making, attention, and working memory. In support, ASD individuals exhibit decreased activation of the prefrontal cortex when performing cognitive tasks [65]. Finally, impaired hormone secretion by the pituitary has long been proposed to contribute to ASD [66], although recent studies have highlighted the potential importance of hormones underlying social behavior, such as oxytocin and vasopressin [67].

Although our AutRef84 expression profile highlights anatomical regions likely to be involved in ASD pathogenesis, it should be interpreted with caution. The four brain regions described above (olfactory bulb, occipital lobe, prefrontal cortex, and pituitary) are the only ones which survived multiple testing in our statistical analysis, but previous evidence suggests that other brain regions functionally relevant to ASD such as the amygdala or cerebellum may also be involved [58], [68]. Likewise, some AutRef84 genes were enriched in non-brain regions, reflecting the pleiotropic expression of genes within the human body. Notably, although expression profiles of diabetes and control datasets also showed enrichment in some brain regions, these brain regions were distinct from of ASD genes and, more importantly were two orders of magnitude less statistically significant. Together, disease specificity of the AutRef84 dual profile indicates the utility of disease-based reference profiling.

Notably, the results of our computational analysis match evidence generated by single gene studies. For example, our expression profile identified the cell adhesion molecule CNTNAP2 as one of the core set of 16 AutRef84 genes enriched in all four significant brain regions, prioritizing it as a high-confidence ASD candidate gene. In support, one recent neuroimaging study used magnetic resonance imaging (MRI) and diffusion tensor imaging to demonstrate that subjects with an ASD-associated single nucleotide polymorphism in CNTNAP2 showed a significant reduction in grey and white matter volume of the occipital and frontal lobes compared with controls [69]. Likewise, a newly published functional MRI showed that another ASD-associated single nucleotide polymorphism of CNTNAP2 altered functional connectivity within the frontal lobe [70]. Additional functional studies will be critical for defining the contribution of our prioritized gene set to molecular pathways dysfunctional in ASD.

In conclusion, our predictive gene map for ASD is a valuable tool by which to prioritize the field of ASD genomics. Our composite reference profile of AutRef84 also provides insight into the molecular etiology of autism, with important implications for drug development. Moreover, our construction and evaluation of AutRef84 can act as a general model for consolidating collective knowledge of a complex disorder into a usable framework of common biological functions.

## Materials and Methods

### Compilation of Gene ASD and Control Reference Sets

We have developed an autism gene database, AutDB [71], [20]–[21]), for ongoing cataloguing of genes linked to ASD. A comprehensive collection of ASD-linked genes was initially compiled from an exhaustive search of the scientific literature from PubMed database at NCBI [72]. The search terms included ‘gene’ AND (‘autism’ OR ‘autistic’) restricted to the titles and abstracts of the publication for retrieval. Furthermore, candidate genes listed in review articles on molecular genetics of ASD, along with cross-references therein, were mapped and added (if new) to our candidate gene list from PubMed searches to compile the most exhaustive gene set. After its first release (Jan 1, 2007), a daily semi-automated search of PubMed with the same keywords was performed to maintain an up-to-date resource of all candidate genes linked to ASD. Additionally, relevant journal articles in the fields of genetics, neurobiology, and psychiatry were screened on a regular basis to enrich the resource. AutRef84 assembled with a data-freeze of May 2010. The authors individually verified all candidate genes included in the reference dataset by reading the full-text primary reference article linking the candidate gene to ASD.

Non-ASD gene sets were compiled using the Online Mendelian Inheritance in Man (OMIM) database [73]. The diabetes dataset consisted of 54 genes verified for linkage association with diabetes and expression in Beta Cells/Islets in the Type 1 Diabetes Database [74] and manually analyzed to exclude any genes whose link to Diabetes was based on genetic association studies.

The non-specific disease reference set was curated by generating a random sampling of 78 genes from the OMIM database which did not show significant association to any one particular disease. The 1000 control gene sets of n = 84 were assembled by randomly sampling the OMIM database.

### Bioconductor Analysis

Enrichment of GO categories was performed using the Conditional HyperGTest in the annotation background of hgu133a as described in the GOStats vignette (S.Falcon and R. Gentlemen, October 3, 2007). The Conditional HyperG Test uses the structure of the GO graph to estimate for each term whether or not there is evidence beyond that which is provided by the term's children to designate the term statistically over-represented. The algorithm conditions on all child terms also significant at the specified *P*-value cut-off. Given a subgraph of one of the three GO ontologies, the terms with no child categories are tested first, followed by the nodes whose children have already been tested. If any of a given node's children tested significant, the appropriate conditioning is performed.

Results of the Conditional HyperG Test were analyzed and visualized in an Excel spreadsheet for GO category, *P*-value, Odds ratio, Expected count, AutRef84 gene count, and annotation category size (**Table S2**). The hierarchical relationship between enriched GO terms was visualized by constructing directed acyclic graphs using GOStats package in Bioconductor (**Figure 3**; **Figures S1 and S2**). Terminal leaves of the graphs were extracted for analysis. The complete list of packages used for GO analysis is shown in the **Methods S1** section.

### DAVID Analysis

We used the Database for Annotation, Visualization, and Integrated Discovery (DAVID) version 6.7 [75] to identify annotation terms significantly enriched in each reference gene set. We used the modified Fisher's exact test, or EASE score, to identify enriched annotation terms derived from GNF_U133A_QUARTILE and gene ontology (GO) annotation terms, which includes Biological Process (BP), Molecular function (MF), and Cellular Component (CC) categories. We used the more specific GO term categories provided by DAVID, called GO FAT, to minimize the redundancy of the more general GO terms in the analysis to increase the specificity of the terms.

A list of gene symbols was generated for each dataset and used as input into DAVID. We used the Functional Annotation Tool, with the Human Genome U133A Plus 2.0 Array as the gene background, to independently analyze each gene set. We used a count threshold of 5 and the default value of 0.1 for the EASE score settings. We also used the Benjamini corrected *P*-value, with p<0.05 as the significance threshold. Significant annotation terms identified in the GNF annotation category were further filtered using the interquartile range of the category size, where the 1^st^ and 3^rd^ quartile were removed from the results. Significant annotation terms in the remaining GO annotation categories were filtered by removing those terms with a category size less than 100 and greater than 1000.

### Genome-Wide Expression Profile

We used the biomaRt package of Bioconductor to screen the human genomic sequence at Ensembl database (NCBI build 36) with the optimized AutRef84 profile. For this analysis, hgu133a was used as the universe.

### Network Visualization

To convey overlapping gene expression between these four regions, we produced a bipartite network consisting of AutRef84 ASD candidate genes and the four brain regions. We assigned links between the genes and their corresponding brain regions. We then assigned a category to each gene with respect to its linked brain regions. (For example, genes expressed in the occipital lobe and in the pituitary were placed in one category, while genes expressed only in the prefrontal cortex were placed in another, and so on.) Next, we used the attribute circle layout in Cytoscape [76] to arrange the nodes in each category into circles. Each circle was then manually repositioned in a location close to its linked brain region or regions. The four brain region nodes were assigned colors based on their positions in an RGB (red, green, blue) cube color space [77]. The color of the nodes in each gene category circle was derived by averaging R, G, and B values of the colors of the linked brain region nodes.

## Supporting Information

Figure S1 **AutRef84 functional profile: graphical representation of over-represented Molecular Function (MF) categories.** Using Bioconductor, we generated directed acyclic graphs based on GO knowledge structure. Enriched GO categories of AutRef84 are represented by rectangular boxes. Terminal nodes are illustrated in yellow. Similar to the AutRef84 BP GO Tree (**Figure 2**), enriched terminal nodes also relate to ion channel activity (*voltage-gated cation channel activity*: 6 genes, P = 7.0×10^−5^; *sodium ion binding*: 5 genes, P = 2.6×10^−4^).
(PDF)

Figure S2 **AutRef84 functional profile: graphical representation of over-represented Cellular Component (CC) categories.** Using Bioconductor, we generated directed acyclic graphs based on GO knowledge structure. Enriched GO categories of AutRef84 are represented by rectangular boxes. Terminal nodes are illustrated in yellow. Like the AutRef84 BP GO Tree (**Figure 2**) and MF GO Tree (**Figure S1**), enriched terminal nodes describe cellular components important for ion channel activity (*cation channel complex*:6 genes, P = 6.0×10^−5^) or ion channel activity/cell adhesion (*synapse*: 7 genes, P = 6.2×10^−4^).
(PDF)

Table S1 **Expanded details of AutRef84 gene set.**
(PDF)

Table S2 **Enriched GO categories of AutRef84 using DAVID analysis.**
(PDF)

Table S3 **KEGG pathway analysis of AutRef84 using Onto Express.**
(PDF)

Table S4 **List of AutRef84 genes expressed within enriched regions.**
(PDF)

Table S5 **Reference set of diabetes-linked genes.**
(PDF)

Table S6 **Non-specific disease gene set.**
(PDF)

Table S7 **Set of 1185 predicted ASD candidate genes matching the AutRef84 functional profile.**
(PDF)

Table S8 **Significantly enriched cytoband categories for the predictive set of 1185 genes using DAVID analysis.**
(PDF)

Table S9 **Set of 460 predicted ASD candidate genes matching the AutRef84 dual profile.**
(PDF)

Table S10 **Previously identified ASD-linked genes matching the AutRef84 dual profile which were not included in the input dataset.**
(PDF)

Methods S1 **Bioconductor Statistics and Packages for GO Enrichment Analysis.**
(DOCX)

## References

[1] Lord C, Cook EH, Leventhal BL, Amaral DG (2000). Autism spectrum disorders.. Neuron.

[2] Van Naarden Braun K, Pettygrove S, Daniels J, Miller L, Nicholas J (2007). Evaluation of a methodology for a collaborative multiple source surveillance network for autism spectrum disorders–Autism and Developmental Disabilities Monitoring Network, 14 sites, United States, 2002.. MMWR Surveill Summ.

[3] Fombonne E (2005). Epidemiology of autistic disorder and other pervasive developmental disorders.. J Clin Psychiatry.

[4] Bailey A, Le Couteur A, Gottesman I, Bolton P, Simonoff E (1995). Autism as a strongly genetic disorder: evidence from a British twin study.. Psychol Med.

[5] Le Couteur A, Bailey A, Goode S, Pickles A, Robertson S (1996). A broader phenotype of autism: the clinical spectrum in twins.. J Child Psychol Psychiatry.

[6] Chakrabarti S, Fombonne E (2001). Pervasive developmental disorders in preschool children.. Jama.

[7] Folstein SE, Rosen-Sheidley B (2001). Genetics of autism: complex aetiology for a heterogeneous disorder.. Nat Rev Genet.

[8] Szatmari P, Paterson AD, Zwaigenbaum L, Roberts W, Brian J (2007). Mapping autism risk loci using genetic linkage and chromosomal rearrangements.. Nat Genet.

[9] Freitag CM (2007). The genetics of autistic disorders and its clinical relevance: a review of the literature.. Mol Psychiatry.

[10] Persico AM, Bourgeron T (2006). Searching for ways out of the autism maze: genetic, epigenetic and environmental clues.. Trends Neurosci.

[11] Yang MS, Gill M (2007). A review of gene linkage, association and expression studies in autism and an assessment of convergent evidence.. Int J Dev Neurosci.

[12] Abrahams BS, Geschwind DH (2008). Advances in autism genetics: on the threshold of a new neurobiology.. Nat Rev Genet.

[13] Vorstman JA, Staal WG, van Daalen E, van Engeland H, Hochstenbach PF (2006). Identification of novel autism candidate regions through analysis of reported cytogenetic abnormalities associated with autism.. Mol Psychiatry.

[14] Sebat J, Lakshmi B, Malhotra D, Troge J, Lese-Martin C (2007). Strong association of de novo copy number mutations with autism.. Science.

[15] Marshall CR, Noor A, Vincent JB, Lionel AC, Feuk L (2008). Structural variation of chromosomes in autism spectrum disorder.. Am J Hum Genet.

[16] Rogers SJ, Wehner DE, Hagerman R (2001). The behavioral phenotype in fragile X: symptoms of autism in very young children with fragile X syndrome, idiopathic autism, and other developmental disorders.. J Dev Behav Pediatr.

[17] Cohen D, Pichard N, Tordjman S, Baumann C, Burglen L (2005). Specific genetic disorders and autism: clinical contribution towards their identification.. J Autism Dev Disord.

[18] Jamain S, Quach H, Betancur C, Rastam M, Colineaux C (2003). Mutations of the X-linked genes encoding neuroligins NLGN3 and NLGN4 are associated with autism.. Nat Genet.

[19] Durand CM, Betancur C, Boeckers TM, Bockmann J, Chaste P (2007). Mutations in the gene encoding the synaptic scaffolding protein SHANK3 are associated with autism spectrum disorders.. Nat Genet.

[20] Basu SN, Kollu R, Banerjee-Basu S (2009). AutDB: a gene reference resource for autism research.. Nucleic Acids Res.

[21] Kumar A, Wadhawan R, Swanwick CC, Basu SN, Kollu R (2011). Animal model integration to AutDB, a genetic database for autism.. BMC Med Genomics.

[22] Crespi B, Stead P, Elliot M (2010). Copy-number variations associated with neuropsychiatric conditions.. Nature.

[23] Elia J, Gai X, Xie HM, Perin JC, Geiger E (2010). Rare structural variants found in attention-deficit hyperactivity disorder are preferentially associated with neurodevelopmental genes.. Mol Psychiatry.

[24] Gillis J, Mistry M, Pavlidis P (2010). Gene function analysis in complex data sets using ErmineJ.. Nat Protoc.

[25] Toro R, Konyukh M, Delorme R, Leblond C, Chaste P (2010). Key role for gene dosage and synaptic homeostasis in autism spectrum disorders.. Trends Genet.

[26] Banerjee-Basu S, Packer A (2010). SFARI Gene: an evolving database for the autism research community.. Dis Model Mech.

[27] Tabuchi K, Blundell J, Etherton MR, Hammer RE, Liu X (2007). A neuroligin-3 mutation implicated in autism increases inhibitory synaptic transmission in mice.. Science.

[28] Ehninger D, Han S, Shilyansky C, Zhou Y, Li W (2008). Reversal of learning deficits in a Tsc2+/− mouse model of tuberous sclerosis.. Nat Med.

[29] El-Fishawy P, State MW (2010). The genetics of autism: key issues, recent findings, and clinical implications.. The Psychiatric clinics of North America.

[30] McClellan J, King MC (2010). Genetic heterogeneity in human disease.. Cell.

[31] Ashburner M, Ball CA, Blake JA, Botstein D, Butler H (2000). Gene ontology: tool for the unification of biology. The Gene Ontology Consortium.. Nat Genet.

[32] Gentleman RC, Carey VJ, Bates DM, Bolstad B, Dettling M (2004). Bioconductor: open software development for computational biology and bioinformatics.. Genome Biol.

[33] Khatri P, Draghici S, Ostermeier GC, Krawetz SA (2002). Profiling gene expression using onto-express.. Genomics.

[34] Cook EH, Courchesne RY, Cox NJ, Lord C, Gonen D (1998). Linkage-disequilibrium mapping of autistic disorder, with 15q11-13 markers.. Am J Hum Genet.

[35] Menold MM, Shao Y, Wolpert CM, Donnelly SL, Raiford KL (2001). Association analysis of chromosome 15 gabaa receptor subunit genes in autistic disorder.. J Neurogenet.

[36] Buxbaum JD, Silverman JM, Smith CJ, Greenberg DA, Kilifarski M (2002). Association between a GABRB3 polymorphism and autism.. Mol Psychiatry.

[37] Nurmi EL, Dowd M, Tadevosyan-Leyfer O, Haines JL, Folstein SE (2003). Exploratory subsetting of autism families based on savant skills improves evidence of genetic linkage to 15q11-q13.. J Am Acad Child Adolesc Psychiatry.

[38] McCauley JL, Olson LM, Delahanty R, Amin T, Nurmi EL (2004). A linkage disequilibrium map of the 1-Mb 15q12 GABA(A) receptor subunit cluster and association to autism.. Am J Med Genet B Neuropsychiatr Genet.

[39] Curran S, Roberts S, Thomas S, Veltman M, Browne J (2005). An association analysis of microsatellite markers across the Prader-Willi/Angelman critical region on chromosome 15 (q11-13) and autism spectrum disorder.. Am J Med Genet B Neuropsychiatr Genet.

[40] Ashley-Koch AE, Mei H, Jaworski J, Ma DQ, Ritchie MD (2006). An analysis paradigm for investigating multi-locus effects in complex disease: examination of three GABA receptor subunit genes on 15q11-q13 as risk factors for autistic disorder.. Ann Hum Genet.

[41] Delahanty RJ, Kang JQ, Brune CW, Kistner EO, Courchesne E (2011). Maternal transmission of a rare GABRB3 signal peptide variant is associated with autism.. Mol Psychiatry.

[42] Nicholas B, Rudrasingham V, Nash S, Kirov G, Owen MJ (2007). Association of Per1 and Npas2 with autistic disorder: support for the clock genes/social timing hypothesis.. Mol Psychiatry.

[43] Persico AM, D'Agruma L, Maiorano N, Totaro A, Militerni R (2001). Reelin gene alleles and haplotypes as a factor predisposing to autistic disorder.. Mol Psychiatry.

[44] Skaar DA, Shao Y, Haines JL, Stenger JE, Jaworski J (2005). Analysis of the RELN gene as a genetic risk factor for autism.. Mol Psychiatry.

[45] Serajee FJ, Zhong H, Mahbubul Huq AH (2006). Association of Reelin gene polymorphisms with autism.. Genomics.

[46] Li H, Li Y, Shao J, Li R, Qin Y (2008). The association analysis of RELN and GRM8 genes with autistic spectrum disorder in Chinese Han population.. Am J Med Genet B Neuropsychiatr Genet.

[47] Melin M, Carlsson B, Anckarsater H, Rastam M, Betancur C (2006). Constitutional downregulation of SEMA5A expression in autism.. Neuropsychobiology.

[48] Pinto D, Pagnametna AT, Klei L, Anney R, Merico D (2010). Functional impact of global rare copy number variation in autism spectrum disorders.. Nature.

[49] Zoghbi HY (2003). Postnatal neurodevelopmental disorders: meeting at the synapse?. Science.

[50] Garber K (2007). Neuroscience. Autism's cause may reside in abnormalities at the synapse.. Science.

[51] Rubenstein JL, Merzenich MM (2003). Model of autism: increased ratio of excitation/inhibition in key neural systems.. Genes Brain Behav.

[52] Volkmar FR, Nelson DS (1990). Seizure disorders in autism.. Journal of the American Academy of Child and Adolescent Psychiatry.

[53] Mouridsen SE, Rich B, Isager T (2010). A longitudinal study of epilepsy and other central nervous system diseases in individuals with and without a history of infantile autism.. Brain Dev epub.

[54] Davidovitch M, Patterson B, Gartside P (1996). Head circumference measurements in children with autism.. Journal of child neurology.

[55] Aylward EH, Minshew NJ, Field K, Sparks BF, Singh N (2002). Effects of age on brain volume and head circumference in autism.. Neurology.

[56] Eriksson PS, Perfilieva E, Bjork-Eriksson T, Alborn AM, Nordborg C (1998). Neurogenesis in the adult human hippocampus.. Nat Med.

[57] Bernier PJ, Bedard A, Vinet J, Levesque M, Parent A (2002). Newly generated neurons in the amygdala and adjoining cortex of adult primates.. Proceedings of the National Academy of Sciences of the United States of America.

[58] Baron-Cohen S, Ring HA, Bullmore ET, Wheelwright S, Ashwin C (2000). The amygdala theory of autism.. Neurosci Biobehavioral Rev.

[59] Mercadante MT, Cysneiros RM, Schwartzman JS, Arida RM, Cavalheiro EA (2008). Neurogenesis in the amygdala: a new etiologic hypothesis of autism?. Med Hypotheses.

[60] Crawley JN (2007). Mouse behavioral assays relevant to the symptoms of autism.. Brain Pathol.

[61] Suzuki Y, Critchley HD, Rowe A, Howlin P, Murphy DG (2003). Impaired olfactory identification in Asperger's syndrome.. J Neuropsychiatry Clin Neurosci.

[62] Bennetto L, Kuschner ES, Hyman SL (2007). Olfaction and taste processing in autism.. Biol Psychiatry.

[63] Nass R, Gross A, Devinsky O (1998). Autism and autistic epileptiform regression with occipital spikes.. Dev Med Child Neurol.

[64] Boucher J, Lewis V (1992). Unfamiliar face recognition in relatively able autistic children.. J Child Psychol Psychiatry.

[65] Silk TJ, Rinehart N, Bradshaw JL, Tonge B, Egan G (2006). Visuospatial processing and the function of prefrontal-parietal networks in autism spectrum disorders: a functional MRI study.. Am J Psychiatry.

[66] Chamberlain RS, Herman BH (1990). A novel biochemical model linking dysfunctions in brain melatonin, proopiomelanocortin peptides, and serotonin in autism.. Biol Psychiatry.

[67] Insel TR (2010). The challenge of translation in social neuroscience: a review of oxytocin, vasopressin, and affiliative behavior.. Neuron.

[68] Amaral DG, Schumann CM, Nordahl CW (2008). Neuroanatomy of autism.. Trends Neurosci.

[69] Tan GC, Doke TF, Ashburner J, Wood NW, Frackowiak RS (2010). Normal variation in fronto-occipital circuitry and cerebellar structure with an autism-associated polymorphism of CNTNAP2.. Neuroimage.

[70] Scott-Van Zeeland AA, Abrahams BS, Alvarez-Retuerto AI, Sonnenblick LI (2010). Altered functional connectivity in frontal lobe circuits is associated with variation in the autism risk gene CNTNAP2.. Sci Transl Med.

[71] AutDB website.. http://www.mindspec.org/autdb.html.

[72] NCBI website.. http://www.ncbi.nih.gov/pubmed.

[73] OMIM website.. http://www.ncbi.nlm.nih.gov/omim.

[74] T1Dbase website.. http://www.t1dbase.org.

[75] DAVID website.. http://david.abcc.ncifcrf.gov/.

[76] Shannon P, Markiel A, Ozier O, Baliga NS, Wang JT (2003). Cytoscape: a software environment for integrated models of biomolecular interaction networks.. Genome Research.

[77] Bales ME, Kaufman DR, Johnson SB (2009). Evaluation of a prototype search and visualization system for exploring scientific communities..

