# Supplementary figures and images for: A Brain Region-Specific Predictive Gene Map for Autism Derived by Profiling a Reference Gene Set
Source: PLoS One. 2011 Dec 9;6(12):e28431. doi: 10.1371/journal.pone.0028431 (PMC3235126; doi:10.1371/journal.pone.0028431)

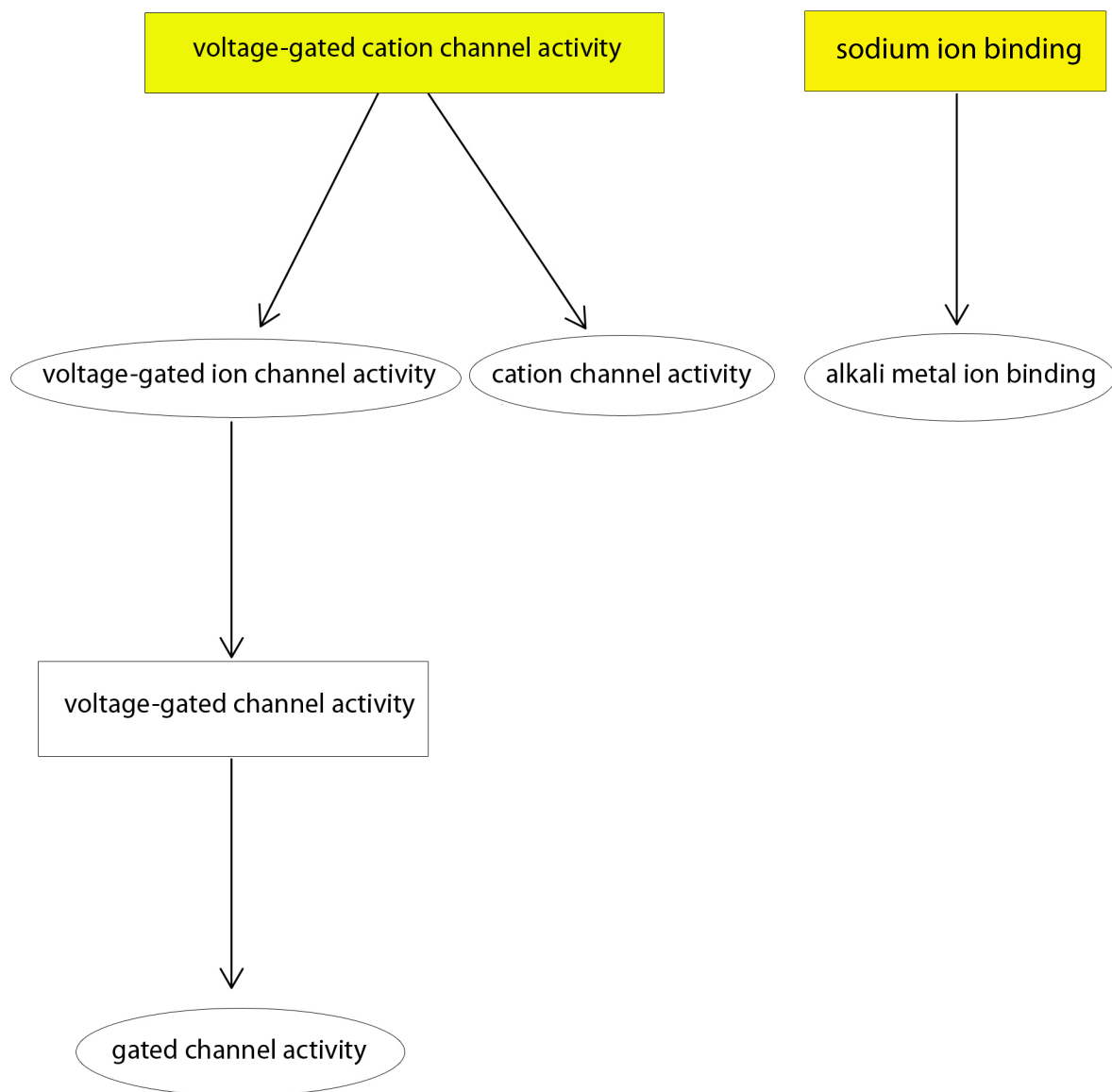

**Supplementary Figure S1**

Supplement: Figure S1 — AutRef84 functional profile: graphical representation of over-represented Molecular Function (MF) categories. Using Bioconductor, we generated directed acyclic graphs based on GO knowledge structure. Enriched GO categories of AutRef84 are represented by rectangular boxes. Terminal nodes are illustrated in yellow. Similar to the AutRef84 BP GO Tree ( Figure 2 ), enriched terminal nodes also relate to ion channel activity (voltage-gated cation channel activity: 6 genes, P = 7.0×10^−5^; sodium ion binding: 5 genes, P = 2.6×10^−4^). (PDF) [file pone.0028431.s001.pdf]

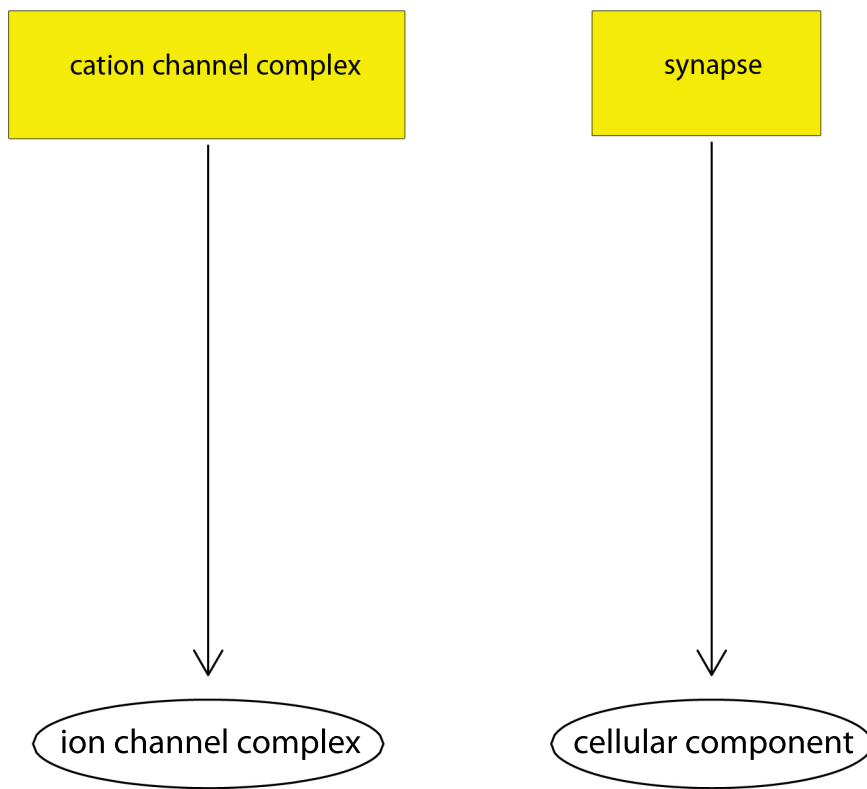

**Supplementary Figure S2**

Supplement: Figure S2 — AutRef84 functional profile: graphical representation of over-represented Cellular Component (CC) categories. Using Bioconductor, we generated directed acyclic graphs based on GO knowledge structure. Enriched GO categories of AutRef84 are represented by rectangular boxes. Terminal nodes are illustrated in yellow. Like the AutRef84 BP GO Tree ( Figure 2 ) and MF GO Tree (Figure S1), enriched terminal nodes describe cellular components important for ion channel activity (cation channel complex:6 genes, P = 6.0×10^−5^) or ion channel activity/cell adhesion (synapse: 7 genes, P = 6.2×10^−4^). (PDF) [file pone.0028431.s002.pdf]
