# Supplementary material for: A Brain Region-Specific Predictive Gene Map for Autism Derived by Profiling a Reference Gene Set
Source: PLoS One. 2011 Dec 9;6(12):e28431. doi: 10.1371/journal.pone.0028431 (PMC3235126; doi:10.1371/journal.pone.0028431)
Supplement: Table S1 — Expanded details of AutRef84 gene set. (PDF) [file pone.0028431.s003.pdf]

Supplementary Table S1. Expanded details of AutRef84 gene set.

	Gene Symbol	Entrez GeneID	Chromosome Number	Chromosomal Location	Genetic Category	Reference PMID
1	ADSL	158	22	22q13.1, 22q13.2	Syndromic	15471876
2	AGAP1	116987	2	2p24.3-p24.1	Rare/Association	15892143
3	AGTR2	186	X	Xq22-q23	Syndromic	12089445
4	AHI1	54806	6	6q23.3	Syndromic/Association	18782849
5	ALDH5A1	7915	6	6p22.2-p22.3	Syndromic	12743223
6	ANKRD11	29123	16	16q24.3	Rare	18252227
7	APC	324	5	5q21-q22	Rare/Association	8071957
8	ARX	170302	X	Xp22.1-22.3	Syndromic	14631200
9	ASTN2	23245	9	118,227,328-119,217,138	Rare	19404257
10	AUTS2	26053	7	7q11.22	Rare	12160723
11	BZRAP1	9256	17	17q22-q23	Rare	19557195
12	C3orf58	205428	3	3q24	Rare	18621663
13	CA6	765	1	1p36.2	Rare	19557195
14	CACNA1C	775	12	12p13.3	Syndromic	15454078
15	CACNA1F	778	X	Xp11.23	Syndromic	11441182
16	CACNA1H	8912	16	16p13.3	Rare	16754686
17	CADM1	23705	11	11q23.2	Rare	18957284
18	CDKL5	6792	X	Xp22	Syndromic	15492925
19	CNTN4	152330	3	3p26-p25	Rare	18349135
20	CNTNAP2	26047	7	7q35-q36	Rare/Association	18179894
21	DHCR7	1717	11	11q13.2-q13.5	Syndromic	16761297
22	DLGAP2	9228	8	8p23	Rare	18252227
23	DMD	1756	X	Xp21.2	Syndromic	16417872
24	DMPK	1760	19	19q13.3	Syndromic	18228241
25	DPP10	57628	2	2q14.1	Rare	18252227
26	DPP6	1804	7	7q36.2	Rare	18252227
27	DPYD	1806	1	1p22	Rare	18252227

28	EIF4E	1977	4	4q21-q25	Rare	19556253
29	FABP5	2171	8	8q21.13	Rare/Association	20057506
30	FABP7	2173	6	6q22-q23	Rare/Association	20057506
31	FBXO40	51725	3	122,794,656-122,831,829	Rare	19404257
32	FHIT	2272	3	3p14.2	Rare	17363630
33	FMR1	2332	x	Xq27.3	Syndromic	9813775
34	GALNT13	114805	2	2q23.3-q24.1	Rare	19557195
35	GRPR	2925	X	Xp22.2-p22.13	Rare	9259269
36	IL1RAPL1	11141	X	Xp22.1-p21.3	Rare	18005360
37	IMMP2L	83943	7	7q31	Rare/Association	11254443
38	JMJD1C	221037	10	10q21.2	Rare	17290275
39	KCNMA1	3778	10	10q22.3	Rare	16946189
40	KIAA1586	57691	6	6p12.1	Rare	19557195
41	MBD1	4152	18	18q21	Rare	19921286
42	MBD3	53615	19	19p13.3	Rare	19921286
43	MBD4	8930	3	3q21-q22	Rare	19921286
44	MCPH1	79648	8	8p23.1	Rare	19793310
45	MDGA2	161357	14	14q21.3	Rare	19557195
46	MECP2	4204	X	Xq28	Syndromic	10508514
47	NBEA	26960	13	13q13	Rare	12746398
48	NF1	4763	17	17q11.2	Syndromic	15389774
49	NLGN1	22871	3	174,805,083-175,483,810	Rare	19404257
50	NLGN3	54413	X	Xq13.1	Rare	12669065
51	NLGN4X	57502	X	Xp22.32-p22.31	Rare	12669065
52	NRXN1	9378	2	2p16.3	Rare	17322880
53	NTNG1	22854	1	1p13.3	Syndromic	15870826
54	ODF3L2	284451	19	19p13.3	Rare	19557195
55	OR1C1	26188	1	1q44	Rare	19557195
56	PARK2	5071	6	161,688,442-163,068,793	Rare	19404257
57	PCDH10	57575	4	4q28.3	Rare	18621663

58	PCDH9	5101	13	13q14.3-q21.1	Rare	18252227
59	PLN	5350	6	6q22.1	Rare	18252227
60	PTCHD1	139411	X	Xp22.11	Rare	18252227
61	PTEN	5728	10	10q23.3	Syndromic	11496368
62	RAB39B	116442	X	Xq28	Rare/Association	20159109
63	RAPGEF4	11069	2	2q31-q32	Rare/No Association	14593429
64	RB1CC1	9821	8	8q11	Rare	18252227
65	RBFOX1	54715	16	16p13.3	Rare/Association	17363630
66	REEP3	221035	10	10q21.3	Rare	17290275
67	RFWD2	64326	1	174,180,590-174,442,993	Rare	19404257
68	RIMS3	9783	1	1pter-p22.2	Rare	19546099
69	RPL10	6134	X	Xq28	Rare	16940977
70	RPS6KA2	6196	6	6q27	Rare	18252227
71	SCN1A	6323	2	2q24.3	Rare	12610651
72	SCN2A	6326	2	2q23-q24	Rare	12610651
73	SEZ6L2	26470	16	16p11.2	Rare	19242545
74	SHANK3	85358	22	22q13.3	Rare	17173049
75	SLC4A10	57282	2	2q23-q24	Rare	17363630
76	SLC6A8	6535	X	Xq28	Syndromic	16601898
77	SLC9A9	285195	3	3q24	Rare	18621663
78	ST7	7982	7	7q31.1-q31.3	Rare	10889047
79	SUCLG2	8801	3	3p14.1	Rare	19557195
80	TMEM195	392636	7	7p21.1	Rare	17363630
81	TSC1	7248	9	9q34	Syndromic	9813776
82	TSC2	7249	16	16p13.3	Syndromic	9813776
83	UBE3A	7337	15	15q11-q13	Rare/Association	11543639
84	XPC	7508	3	3p25	Syndromic	9804340
