# Supplementary material for: A Brain Region-Specific Predictive Gene Map for Autism Derived by Profiling a Reference Gene Set
Source: PLoS One. 2011 Dec 9;6(12):e28431. doi: 10.1371/journal.pone.0028431 (PMC3235126; doi:10.1371/journal.pone.0028431)
Supplement: Table S2 — Enriched GO categories of AutRef84 using DAVID analysis. (PDF) [file pone.0028431.s004.pdf]

Supplementary Table S2. Enriched GO categories of AutRef84 using DAVID analysis.

GO Category	GO ID	Annotation term	Genes in list with term	Category size	p-value*
BP	GO:0019226	transmission of nerve impulse	14	346	2.08E-05
	GO:0050877	neurological system process	21	875	2.56E-05
	GO:0044057	regulation of system process	10	305	0.00620
	GO:0007610	behavior	12	458	0.00631
	GO:0007611	learning or memory	7	111	0.00678
	GO:0050801	ion homeostasis	11	402	0.00832
	GO:0007268	synaptic transmission	10	295	0.00834
	GO:0030182	neuron differentiation	11	426	0.01196
	GO:0042592	homeostatic process	14	739	0.01418
	GO:0019725	cellular homeostasis	11	458	0.01498
	GO:0030001	metal ion transport	11	449	0.01506
	GO:0030030	cell projection organization	10	359	0.01510
	GO:0043062	extracellular structure organization	7	163	0.02020
	GO:0048666	neuron development	9	331	0.02726
	GO:0048878	chemical homeostasis	11	503	0.02736
	GO:0031175	neuron projection development	8	249	0.02763
	GO:0006812	cation transport	11	534	0.03630
	GO:0042391	regulation of membrane potential	6	133	0.04484
	GO:0048812	neuron projection morphogenesis	7	206	0.04598
	GO:0006873	cellular ion homeostasis	9	368	0.04607
	GO:0055082	cellular chemical homeostasis	9	374	0.04650
CC	GO:0043005	neuron projection	13	339	9.29E-05
	GO:0045202	synapse	10	348	0.01060
	GO:0042995	cell projection	14	687	0.01293
	GO:0030424	axon	7	159	0.01421
	GO:0034703	cation channel complex	6	129	0.03228
