# Supplementary material for: A Brain Region-Specific Predictive Gene Map for Autism Derived by Profiling a Reference Gene Set
Source: PLoS One. 2011 Dec 9;6(12):e28431. doi: 10.1371/journal.pone.0028431 (PMC3235126; doi:10.1371/journal.pone.0028431)
Supplement: Table S3 — KEGG pathway analysis of AutRef84 using Onto Express. (PDF) [file pone.0028431.s005.pdf]

Supplementary Table S3. KEGG pathway analysis of AutRef84 using Onto Express

Rank	Pathway Name	Impact Factor	# Genes in Pathway	# Input Genes in Pathway	% Pathway Genes in Input	corrected p-value	Unique Pathway-id
1	Cell adhesion molecules (CAMs)	11.794	134	6	4.478	7.55E-06	1:04514
2	mTOR signaling pathway	10.325	52	4	7.692	3.28E-05	1:04150
3	Calcium signaling pathway	7.719	182	5	2.747	4.44E-04	1:04020
4	p53 signaling pathway	6.311	69	3	4.348	0.0018165	1:04115
5	MAPK signaling pathway	5.935	272	5	1.838	0.0026451	1:04010
