# Supplementary material for: A Brain Region-Specific Predictive Gene Map for Autism Derived by Profiling a Reference Gene Set
Source: PLoS One. 2011 Dec 9;6(12):e28431. doi: 10.1371/journal.pone.0028431 (PMC3235126; doi:10.1371/journal.pone.0028431)
Supplement: Table S4 — List of AutRef84 genes expressed within enriched regions. (PDF) [file pone.0028431.s006.pdf]

Supplementary Table S4. List of AutRef84 genes expressed within enriched regions

Olfactory Bulb_3rd	Occipital Lobe_3rd	Prefrontal Cortex_3rd	Pituitary_3rd	fetalliver_3rd	Colorectal Adenocarcinoma_3rd	adrenal gland_3rd	Thymus_3rd	Uterus Corpus_3rd
**Brain**	**Brain**	**Brain**	**Brain**	**System**	**System**	**System**	**System**	**System**
SCN1A	SCN1A	FBXO40	SCN1A	SCN1A	SCN1A	FHIT	SCN1A	KCNMA1
CADM1	CADM1	CADM1	UBE3A	CADM1	CADM1	SCN1A	CADM1	SUCLG2
UBE3A	NBEA	UBE3A	NBEA	UBE3A	FBXO40	CADM1	NBEA	UBE3A
NBEA	ASTN2	NBEA	ASTN2	NBEA	NBEA	NBEA	ASTN2	NF1
ASTN2	RIMS3	RIMS3	RIMS3	RIMS3	ASTN2	ASTN2	RIMS3	FMR1
RIMS3	SEZ6L2	SEZ6L2	SEZ6L2	DMPK	RIMS3	RIMS3	DMPK	MECP2
SEZ6L2	AGTR2	AGTR2	ANKRD11	SEZ6L2	SEZ6L2	SEZ6L2	SEZ6L2	MBD4
DMD	RB1CC1	DMD	CNTNAP2	DMD	ANKRD11	DHCR7	DHCR7	NBEA
RB1CC1		GRPR	RAPGEF4	RB1CC1	GRPR	CNTNAP2	GRPR	PTEN
CNTNAP2	RAPGEF4	CNTNAP2	DPP6	CNTNAP2	CNTNAP2	RAPGEF4	AUTS2	MBD1
AUTS2	DPP6	RAPGEF4	IL1RAPL1	AUTS2	RAPGEF4	DPP6	CNTNAP2	DMPK
RAPGEF4		DPP6	APC	DPP6	DPP6	APC	RAPGEF4	XPC
DPP6	APC	IL1RAPL1	DLGAP2	APC	IL1RAPL1	KCNMA1	DPP6	EIF4E
IL1RAPL1	KCNMA1	APC	SCN2A	DLGAP2	APC	ALDH5A1	APC	RPS6KA2
APC	DLGAP2	KCNMA1	NF1	NF1	KCNMA1	DLGAP2	KCNMA1	PLN
ALDH5A1	NF1	SCN2A	MECP2	FMR1	ALDH5A1	NF1	NF1	DMD
DLGAP2	FMR1	NF1	PCDH9	SCN2A	DLGAP2	FMR1	BZRAP1	RB1CC1
NF1	SCN2A	BZRAP1	NLGN3	NLGN1	NF1	SCN2A	MECP2	TSC2
FMR1	NLGN1	PCDH9	NRXN1	MECP2	SCN2A	NLGN1	AHI1	ANKRD11
SCN2A	BZRAP1	NLGN3	EIF4E	BZRAP1	BZRAP1	BZRAP1	PCDH9	ADSL
NLGN1	MECP2	NRXN1	TSC1	AHI1	MECP2	MECP2	NLGN3	DPYD
MECP2	AHI1	SLC4A10	SLC6A8	PCDH9	AHI1	PCDH9	NRXN1	JMJD1C
AHI1	PCDH9	A2BP1	RPS6KA2	NRXN1	PCDH9	NRXN1	EIF4E	CACNA1C
PCDH9	NLGN3	NLGN4X	A2BP1	MBD3	NLGN3	MBD3	SLC6A8	DPP6
NLGN3	NRXN1	FABP7	NLGN4X	MBD1	NRXN1	MBD1	RPS6KA2	
NRXN1	SLC6A8		FABP7	EIF4E	EIF4E	EIF4E	A2BP1	
EIF4E	RPS6KA2			TSC1	TSC1	TSC1	CA6	
TSC1	A2BP1			RPS6KA2	SLC6A8	SLC6A8	NLGN4X	
SLC6A8	CA6			A2BP1	RPS6KA2	RPS6KA2	FABP7	
RPS6KA2	NLGN4X			NLGN4X	A2BP1	A2BP1		
A2BP1	ADSL			TSC2	NLGN4X	NLGN4X		
NLGN4X	FABP7			CACNA1H	ADSL	TSC2		
FABP7				JMJD1C	CACNA1C	FABP7		
				FABP7	FABP7			
				FABP5				
				ST7				
