# Supplementary material for: A Brain Region-Specific Predictive Gene Map for Autism Derived by Profiling a Reference Gene Set
Source: PLoS One. 2011 Dec 9;6(12):e28431. doi: 10.1371/journal.pone.0028431 (PMC3235126; doi:10.1371/journal.pone.0028431)
Supplement: Table S5 — Reference set of diabetes-linked genes. (PDF) [file pone.0028431.s007.pdf]

Supplementary Table S5. Reference set of diabetes-linked genes

	Gene Symbol	Entrez Gene ID	OMIM ID
1	ABCA1	19	600046
2	ACE	1636	106180
3	AKT2	208	164731
4	ALMS1	7840	606844
5	APOC2	344	608083
6	AQP2	359	107777
7	AVP	551	192340
8	AVPR2	554	300538
9	BSCL2	26580	606158
10	BTK	695	300300
11	CEL	1056	114840
12	CFTR	1080	602421
13	CP	1356	117700
14	CX3CR1	1524	601470
15	CYP19A1	1588	107910
16	DYT1	1861	605204
17	EIF2AK3	9451	604032
18	EPHX2	2053	132811
19	EPO	2056	133170
20	FGF8	2253	600483
21	FOXC2	2303	602402
22	GCCR	2908	138040
23	GCGR	2642	138033
24	GLA	2717	300644
25	GPD2	2820	138430
26	HAMP	57817	606464
27	HBA1	3039	141800

**28**	HBA2	3040	141850
**29**	HBB	3043	141900
**30**	HFE	3077	235200
**31**	HNF1A	6927	142410
**32**	HNF1B	6928	189907
**33**	INSR	3643	147670
**34**	IPF1	3651	600733
**35**	ITGB3	3690	173470
**36**	KLF11	8462	603301
**37**	LDHB	3945	150100
**38**	LIG4	3981	601837
**39**	LMNB2	84823	150341
**40**	MAPK8IP1	9479	604641
**41**	OB	1009	164160
**42**	PCNT2	5116	605925
**43**	PNPLA2	57104	609059
**44**	PTF1A	256297	607194
**45**	PTPRC	5788	151460
**46**	RECQL2	641	604611
**47**	SLC19A2	10560	603941
**48**	SLC2A4	6517	138190
**49**	SMARCB1	6598	601607
**50**	SOD2	6648	147460
**51**	SPINK1	6690	167790
**52**	TGIF	7050	602630
**53**	WFS1	7466	606201
**54**	ZFP57	346171	612192
