# Supplementary material for: A Brain Region-Specific Predictive Gene Map for Autism Derived by Profiling a Reference Gene Set
Source: PLoS One. 2011 Dec 9;6(12):e28431. doi: 10.1371/journal.pone.0028431 (PMC3235126; doi:10.1371/journal.pone.0028431)
Supplement: Table S6 — Non-specific disease gene set. (PDF) [file pone.0028431.s008.pdf]

Supplementary Table S6. Non-specific disease gene set.

	Gene Symbol	Entrez Gene ID	OMIM ID	Disease Name
1	A2M	2	103950	Alpha-2-macroglobulin deficiency
2	ABCA1	19	600046	Coronary heart disease in familial hypercholesterolemia
3	ACVR2B	93	602730	Heterotaxy, Visceral
4	JAG1	182	182	Deafness, Congenital heart defects
5	AHCY	191	180960	Hypermethioninemia with deficiency of S-Adenosylhomocysteine hydrolase
6	ALDH2	217	100650	Esophageal cancer
7	APOB	338	107730	Familial hypobetalipoproteinemia
8	APOE	348	107741	Deficiency or defect of apolipoprotein E
9	BCHE	590	177400	Butyrylcholinesterase deficiency
10	C3	718	120700	Complement component 3 deficiency
11	C5	727	120900	Susceptibility to liver fibrosis
12	SLC25A20	788	212138	CACT Deficiency
13	CASR	846	601199	Hypercalciuric hypercalcemia
14	CBFB	865	121360	Delayed cranial ossification due to CBFB haploinsufficiency
15	CBS	875	236200	Homocystinuria due to cystathionine beta-synthase deficiency
16	COL1A1	1277	120150	OI/EDS Combined syndrome
17	COMT	1312	116790	Low COMT activity in red cells
18	CYP2A6	1548	122720	Poor metabolism of tegafur
19	CYP2A7	1549	608054	
20	CYP2D6	1565	124030	
21	CYP19A1	1588	107910	Aromatase deficiency
22	ACE	1636	106180	IgA nephropathy with progression to renal failure

**23**	ERCC6	2074	609413	Age-related macular degeneration
**24**	ESR1	2099	133430	Estrogen resistance
**25**	F10	2159	227600	Stuart-Prower factor deficiency
**26**	FANCC	2176	227645	Fanconi pancytopenia Type 3
**27**	FAH	2184	276700	Tyrosinemia, Type I
**28**	FGA	2243	134820	Dysfibrinogenemia causing recurrent thrombosis
**29**	G6PC	2538	232200	Glycogen storage disease 1
**30**	G6PD	2539	305900	Nonspherocytic hemolytic anemia
**31**	GNAS	2778	139320	Prolonged bleeding time, brachydactyly, and mental retardation
**32**	HBA1	3039	141800	Alpha-thalassemias
**33**	HBA1	3040	141800	Alpha-thalassemias
**34**	HBB	3043	141900	Beta-thalassemias
**35**	HFE	3077	235200	Hemochromatosis
**36**	HMGCL	3155	246450	HMG-CoA lyase deficiency
**37**	HMOX1	3162	141250	Heme oxygenase 1 deficiency
**38**	IGFALS	3483	601489	Acid-labile subunit deficiency
**39**	INS	3630	176730	Hyperproinsulinemia
**40**	IRS1	3667	147545	Coronary Artery Disease
**41**	ITGB3	3690	173470	Thrombocytopenia
**42**	JAK2	3717	147796	Myeloproliferative disorder with erythrocytosis
**43**	KAL1	3730	308700	Kallmann syndrome interval gene 1
**44**	LDHB	3945	150100	Lactate dehydrogenase B
**45**	LEP	3952	164160	Obese

**46**	LEPR	3953	601007	Morbid obesity
**47**	LHB	3972	152780	Male pseudohermaphroditism due to defective LH molecule
**48**	LHCGR	3973	152790	
**49**	LIPC	3990	151670	Hepatic triglyceride lipase deficiency
**50**	MBL2	4153	154545	Mannose-binding protein deficiency
**51**	MEN1	4221	131100	Multiple endocrine neoplasia Type I
**52**	NOS3	4846	163729	Coronary artery spasm 1
**53**	NP	4860	164050	
**54**	PDE6B	5158	180072	Retinitis pigmentosa 40
**55**	PEX12	5193	601758	Peroxisome biogenesis disorder
**56**	PIGA	5277	311770	
**57**	PKD1	5310	601313	Polycystic Kidney Disease I
**58**	PON1	5444	168820	Variation in PON1 enzyme activity
**59**	PPARA	5465	170998	Hyperapobetalipoproteinemia
**60**	PYGL	5836	232700	Glycogen storage disease VI
**61**	RB1	5925	180200	Retinoblastoma
**62**	RET	5979	164761	Hirschsprung disease
**63**	RHO	6010	180380	Retinitis pigmentosa 4
**64**	RPE65	6121	180069	Retinitis pigmentosa 20
**65**	SLC4A1	6521	109270	Acanthocytosis
**66**	TERT	7015	187270	Susceptibility to coronary artery disease
**67**	LEFTY2	7044	601877	Left-right axis malformations
**68**	TTR	7276	176300	Transthyretin

**69**	LRAT	9227		
**70**	CYP7B1	9420	603711	Congenital Bile acid synthesis defect
**71**	KCNE2	9992	603796	Long QT syndrome 6
**72**	CHEK2	11200	604373	Susceptibility to breast and colorectal cancer
**73**	AMACR	23600	604489	Alpha-methylacyl-CoA racemase deficiency
**74**	BLNK	29760	604515	Hypoglobulinemia and absent B cells
**75**	UPB1	51733	606673	
**76**	C3	653879	120700	C3 deficiency
**77**	INS	723961	176730	Hyperproinsulinemia
**78**	C3	100133511	120700	C3 deficiency
