# Supplementary material for: A Brain Region-Specific Predictive Gene Map for Autism Derived by Profiling a Reference Gene Set
Source: PLoS One. 2011 Dec 9;6(12):e28431. doi: 10.1371/journal.pone.0028431 (PMC3235126; doi:10.1371/journal.pone.0028431)
Supplement: Table S7 — Set of 1185 predicted ASD candidate genes matching the AutRef84 functional profile. (PDF) [file pone.0028431.s009.pdf]

Supplementary Table S7. Set of 1185 predicted ASD candidate genes matching the AutRef84 functional profile.

Gene Symbol	Entrez Gene ID	Chromosome	Band	Start Position	End Position
AATF	26574	17	q12	35306175	35414170
ABI1	10006	10	p12.1	27035522	27149959
ABL1	25	9	q34.12	133589333	133763062
ABL2	27	1	q25.2	179068462	179198819
ACAN	176	15	q26.1	89346674	89418583
ACCN1	40	17	q12	31340105	32483551
ACCN2	41	12	q13.12	50451487	50477393
ACCN3	9311	7	q36.1	150745379	150749843
ACCN4	55515	2	q35	220378892	220403494
ACCN5	51802	4	q32.1	156750881	156787425
ACHE	43	7	q22.1	100487615	100494594
ACTN2	88	1	q43	236849808	236927931
ACVR1	90	2	q24.1	158592958	158732374
ACVR1B	91	12	q13.13	52345486	52390857
ADA	100	20	q13.12	43248163	43280383
ADAM12	8038	10	q26.2	127700950	128077065
ADAM15	8751	1	q22	155023177	155035252
ADAM17	6868	2	p25.1	9628615	9695921
ADAM2	2515	8	p11.22	39601254	39695808
ADAM22	53616	7	q21.12	87563458	87832204
ADAM23	8745	2	q33.3	207308263	207485851
ADAM9	8754	8	p11.22	38854388	38962663
ADD1	118	4	p16.3	2845584	2931803
ADRBK1	156	11	q13.2	67033905	67054029
AEBP1	165	7	p13	44143960	44154161
AGGF1	55109	5	q13.3	76325076	76361059
AGRN	375790	1	p36.33	955503	991496
AIMP1	9255	4	q24	107236701	107270383
AJAP1	55966	1	p36.32	4714792	4843850
AKAP9	10142	7	q21.2	91570181	91739989
ALCAM	214	3	q13.11	105085713	105295744
ALDH1A2	8854	15	q21.3	58245622	58358616
ALDH3A2	224	17	p11.2	19551459	19580909
ALKBH1	8846	14	q24.3	78138749	78174356
AMBN	258	4	q13.3	71457973	71473005
AMBP	259	9	q32	116822407	116840752
AMELX	265	X	p22.2	11311533	11318881
AMICA1	120425	11	q23.3	118064442	118095809
AMIGO1	57463	1	p13.3	110046797	110052360

AMIGO2	347902	12	q13.11	47469490	47473734
AMIGO3	386724	3	p21.31	49754268	49757128
AMPH	273	7	p14.1	38423305	38671167
AMTN	401138	4	q13.3	71384257	71398459
ANK3	288	10	q21.2	61788159	62493248
ANKS1B	56899	12	q23.1	99129338	100378015
AOC3	8639	17	q21.31	41003201	41010138
APBA1	320	9	q21.12	72045204	72287222
APBB1	322	11	p15.4	6413729	6440644
APBB2	323	4	p13	40812044	41218731
APLP1	333	19	q13.12	36359401	36370699
APP	351	21	q21.3	27252861	27543446
ARC	23237	8	q24.3	143692410	143695833
ARF6	382	14	q21.3	50359810	50361490
ARHGAP32	9743	11	q24.3	128834955	129062093
ARHGAP5	394	14	q12	32546495	32628934
ARHGEF40	55701	14	q11.2	21538527	21558036
ARNT2	9915	15	q25.1	80696692	80890269
ARR3	407	X	q13.1	69488155	69501690
ARRB1	408	11	q13.4	74976482	75062873
ARRB2	409	17	p13.2	4613784	4624795
ARSA	410	22	q13.33	51063446	51066607
ARSB	411	5	q14.1	78073032	78281910
ARVCF	421	22	q11.21	19957419	20004331
ASCL1	429	12	q23.2	103351452	103354287
ASCL2	430	11	p15.5	2289725	2292182
ATN1	1822	12	p13.31	7033626	7051482
ATOH1	474	4	q22.2	94750042	94751221
ATP10D	57205	4	p12	47487305	47595503
ATP12A	479	13	q12.12	25254695	25285918
ATP13A1	57130	19	p13.11	19756007	19774502
ATP13A2	23400	1	p36.13	17312453	17338423
ATP13A3	79572	3	q29	194123401	194219093
ATP13A4	84239	3	q29	193119866	193310900
ATP13A5	344905	3	q29	192992579	193096632
ATP1A1	476	1	p13.1	116915290	116952883
ATP1A2	477	1	q23.2	160085549	160113381
ATP1A3	478	19	q13.2	42470734	42498384
ATP1A4	480	1	q23.2	160121352	160156767
ATP1B1	481	1	q24.2	169074935	169101960
ATP1B2	482	17	p13.1	7554254	7561087

ATP1B3	483	3	q23	141594966	141645356
ATP1B4	23439	X	q24	119495967	119516226
ATP2A1	487	16	p11.2	28889804	28915787
ATP2A2	488	12	q24.11	110719032	110788893
ATP2A3	489	17	p13.2	3822943	3867736
ATP2B1	490	12	q21.33	89981828	90049844
ATP2B2	491	3	p25.3	10365707	10749716
ATP2B3	492	X	q28	152783134	152848397
ATP2B4	493	1	q32.1	203595689	203713209
ATP2C1	27032	3	q22.1	130569439	130735556
ATP2C2	9914	16	q24.1	84402133	84497792
ATP4A	495	19	q13.12	36041096	36054560
ATP4B	496	13	q34	114303173	114312501
ATXN10	25814	22	q13.31	46067679	46241187
AZGP1	563	7	q22.1	99564343	99573780
B3GNT5	84002	3	q27.1	182971032	183016292
B4GALT1	2683	9	p21.1	33110635	33167354
BAG1	573	9	p13.3	33247818	33264761
BAI1	575	8	q24.3	143530791	143626370
BARHL2	343472	1	p22.2	91177159	91182794
BCAM	4059	19	q13.32	45312338	45324677
BCAN	63827	1	q23.1	156611182	156629324
BCAR1	9564	16	q23.1	75262928	75301951
BGLAP	632	1	q22	156211753	156213112
BHLHE22	27319	8	q12.3	65492814	65496181
BOC	91653	3	q13.2	112929850	113006303
BRAP	8315	12	q24.12	112079951	112123790
BSN	8927	3	p21.31	49591922	49708978
BTBD9	114781	6	p21.2	38136227	38607924
BTD	686	3	p25.1	15642848	15687329
BTG2	7832	1	q32.1	203274619	203278730
BTG4	54766	11	q23.1	111338255	111383064
BVES	11149	6	q21	105544697	105584560
BYSL	705	6	p21.1	41888926	41900784
C1orf38	9473	1	p35.3	28199055	28213196
C21orf29	54084	21	q22.3	45917775	46131495
C7orf16	10842	7	p14.3	31726329	31748069
CABP1	9478	12	q24.31	121078355	121105127
CABP4	57010	11	q13.2	67222818	67226651
CACNB4	785	2	q23.3	152689290	152955593
CADM3	57863	1	q23.2	159141399	159173103

CADM4	199731	19	q13.31	44126522	44143991
CADPS	8618	3	p14.2	62384021	62861064
CADPS2	93664	7	q31.32	121958481	122526554
CAMK2A	815	5	q32	149599054	149669854
CAMK2N1	55450	1	p36.12	20808884	20812713
CASK	8573	X	p11.4	41374187	41782716
CASS4	57091	20	q13.2	54987168	55034395
CATSPER3	347732	5	q31.1	134303596	134347392
CATSPER4	378807	1	p36.11	26517052	26529459
CAV1	857	7	q31.2	116164839	116201233
CBLN1	869	16	q12.1	49312435	49315742
CBLN3	643866	14	q12	24895742	24898731
CBLN4	140689	20	q13.2	54572496	54580528
CCDC64	92558	12	q24.23	120427648	120532299
CCL11	6356	17	q12	32612761	32615143
CCL2	6347	17	q12	32582313	32584222
CCL4	6351	17	q12	34431220	34433665
CCL5	6352	17	q12	34198495	34207797
CCR1	1230	3	p21.31	46243200	46249887
CCR3	1232	3	p21.31	46205096	46308111
CCR8	1237	3	p22.2	39371197	39375002
CD151	977	11	p15.5	832843	838834
CD164	8763	6	q21	109687717	109703762
CD2	914	1	p13.1	117297007	117311850
CD209	30835	19	p13.2	7804879	7812464
CD22	933	19	q13.12	35820079	35838262
CD226	10666	18	q22.2	67528097	67624160
CD300A	11314	17	q25.1	72462522	72480933
CD33	945	19	q13.41	51728335	51743274
CD36	948	7	q21.11	79998891	80308593
CD4	920	12	p13.31	6898651	6929976
CD44	960	11	p13	35160417	35253946
CD47	961	3	q13.12	107761941	107809935
CD58	965	1	p13.1	117057157	117113715
CD6	923	11	q12.2	60739115	60787846
CD72	971	9	p13.3	35609530	35646807
CD84	8832	1	q23.3	160510888	160549294
CD9	928	12	p13.31	6308881	6347427
CD96	10225	3	q13.13	111260926	111384597
CD97	976	19	p13.12	14492213	14519531
CD99	4267	X	p22.33	2609220	2659350

CD99L2	83692	X	q28	149934810	150067179
CDH1	999	16	q22.1	68771128	68869444
CDH11	1009	16	q21	64977656	65156101
CDH12	1010	5	p14.3	21750777	22853731
CDH13	1012	16	q23.3	82882260	83830204
CDH15	1013	16	q24.3	89238175	89261900
CDH16	1014	16	q22.1	66942025	66952860
CDH17	1015	8	q22.1	95139399	95229531
CDH18	1016	5	p14.3	19473060	20575982
CDH2	1000	18	q12.1	25530930	25757410
CDH22	64405	20	q13.12	44802372	44880334
CDH23	64072	10	q22.1	73156694	73575702
CDH24	64403	14	q11.2	23516271	23526747
CDH3	1001	16	q22.1	68678151	68732971
CDH4	1002	20	q13.33	59827559	60512277
CDH5	1003	16	q21	66400525	66438686
CDH6	1004	5	p13.3	31193857	31329253
CDH7	1005	18	q22.1	63417488	63552376
CDH8	1006	16	q21	61685917	62070739
CDHR1	92211	10	q23.1	85954410	85979377
CDHR2	54825	5	q35.2	175969512	176022975
CDHR3	222256	7	q22.3	105517242	105676877
CDHR4	389118	3	p21.31	49828165	49837268
CDHR5	53841	11	p15.5	616575	626078
CDK5	1020	7	q36.1	150750899	150755617
CDK5R1	8851	17	q11.2	30813637	30818274
CEBPB	1051	20	q13.13	48807376	48809212
CELSR1	9620	22	q13.31	46755733	46933067
CELSR2	1952	1	p13.3	109792641	109818377
CELSR3	1951	3	p21.31	48673902	48700348
CEP120	153241	5	q23.2	122680579	122759286
CERCAM	51148	9	q34.11	131174030	131199626
CFDP1	10428	16	q23.1	75327608	75467383
CHAT	1103	10	q11.23	50817141	50873150
CHD7	55636	8	q12.1	61591339	61779463
CHL1	10752	3	p26.3	238279	451090
CHRD	8646	3	q27.1	184097861	184108524
CHRDL1	91851	X	q23	109917084	110039286
CHRM5	1133	15	q14	34261089	34357295
CHRNA10	57053	11	p15.4	3686818	3692614
CHRNA2	1135	8	p21.2	27317279	27337400

CHRNA3	1136	15	q25.1	78885394	78913637
CHRNA4	1137	20	q13.33	61974665	62009753
CHRNA5	1138	15	q25.1	78857862	78887611
CHRNA6	8973	8	p11.21	42607782	42623619
CHRNA7	1139	15	q13.3	32322701	32464722
CHRNA9	55584	4	p14	40337346	40357234
CHRNB1	1140	17	p13.1	7348380	7361026
CHRNB2	1141	1	q21.3	154540257	154552502
CHRNB3	1142	8	p11.21	42552562	42592209
CHRNB4	1143	15	q25.1	78916461	78933587
CHRND	1144	2	q37.1	233390703	233401377
CHRNE	1145	17	p13.2	4801069	4806369
CHRNG	1146	2	q37.1	233404437	233411113
CHST10	9486	2	q11.2	101008327	101034118
CHST4	10164	16	q22.2	71560036	71572488
CHST8	64377	19	q13.11	34112861	34264413
CIB1	10519	15	q26.1	90773207	90777279
CISH	1154	3	p21.2	50643921	50649262
CLCA2	9635	1	p22.3	86889769	86922241
CLDN1	9076	3	q28	190023490	190040264
CLDN10	9071	13	q32.1	96085858	96231906
CLDN11	5010	3	q26.2	170136653	170578169
CLEC4A	50856	12	p13.31	8276226	8291203
CLN5	1203	13	q22.3	77564795	77576652
CLSTN1	22883	1	p36.22	9789084	9884584
CLSTN2	64084	3	q23	139654027	140286919
CLSTN3	9746	12	p13.31	7282967	7311526
CNTN1	1272	12	q12	41302156	41464094
CNTN2	6900	1	q32.1	205012415	205047144
CNTN3	5067	3	p12.3	74311719	74570291
CNTN5	53942	11	q22.1	99690263	100227473
CNTN6	27255	3	p26.3	1134260	1445901
CNTNAP1	8506	17	q21.2	40834632	40852010
CNTNAP3	79937	9	p13.1	39072764	39288456
CNTNAP3B	389722	9	p11.2	43684902	43924049
CNTNAP4	85445	16	q23.1	76311176	76593135
CNTNAP5	129684	2	q14.3	124782864	125672864
COL11A1	1301	1	p21.1	103342023	103574052
COL12A1	1303	6	q14.1	75794042	75915767
COL14A1	7373	8	q24.12	121072019	121384275
COL15A1	1306	9	q22.33	101705461	101833069

COL16A1	1307	1	p35.2	32117848	32169920
COL18A1	80781	21	q22.3	46825052	46933634
COL19A1	1310	6	q13	70576463	70919679
COL20A1	57642	20	q13.33	61924538	61966203
COL21A1	81578	6	p12.1	55921388	56258892
COL22A1	169044	8	q24.3	139600478	139926249
COL24A1	255631	1	p22.3	86194916	86622626
COL27A1	85301	9	q32	116917840	117074791
COL28A1	340267	7	p21.3	7395834	7575484
COL4A3	1285	2	q36.3	228029281	228179508
COL4A6	1288	X	q22.3	107386780	107682727
COL5A1	1289	9	q34.3	137533620	137736686
COL5A3	50509	19	p13.2	10070237	10121147
COL6A1	1291	21	q22.3	47401651	47424964
COL6A3	1293	2	q37.3	238232646	238323018
COL6A6	131873	3	q22.1	130279178	130395888
COL7A1	1294	3	p21.31	48601506	48632700
COL8A1	1295	3	q12.1	99357319	99518070
COL9A1	1297	6	q13	70924764	71012786
COLQ	8292	3	p25.1	15491640	15563258
COMP	1311	19	p13.11	18893584	18902114
COPS2	9318	15	q21.1	49417473	49447854
COQ7	10229	16	p12.3	19078921	19091417
CPEB1	64506	15	q25.2	83211954	83316728
CPLX3	594855	15	q24.1	75118887	75124143
CPLX4	339302	18	q21.32	56962634	56985881
CPXM1	56265	20	p13	2774715	2781283
CPXM2	119587	10	q26.13	125472792	125699779
CRIPT	9419	2	p21	46843555	46852881
CSF3R	1441	1	p34.3	36931644	36948879
CTBP2	1488	10	q26.13	126676418	126849739
CTGF	1490	6	q23.2	132269316	132272513
CTNNA1	1495	5	q31.2	137946656	138270723
CTNNA2	1496	2	p12	79412357	80875905
CTNNA3	29119	10	q21.3	67679719	69455927
CTNNAL1	8727	9	q31.3	111704851	111775809
CTNNB1	1499	3	p22.1	41236328	41301587
CTNND1	1500	11	q12.1	57529234	57586651
CTNND2	1501	5	p15.2	10971952	11904155
CUZD1	50624	10	q26.13	124591665	124610309
CX3CL1	6376	16	q21	57406375	57418960

CX3CR1	1524	3	p22.2	39304985	39323186
CXADR	1525	21	q21.1	18884700	18965897
CXCL12	6387	10	q11.21	44865613	44881941
CXCR3	2833	X	q13.1	70835766	70838367
CYFIP1	23191	15	q11.2	22892663	23006016
CYP26A1	1592	10	q23.33	94833232	94837647
CYP26C1	340665	10	q23.33	94820565	94829293
CYR61	3491	1	p22.3	86046444	86049645
CYTSA	135	22	q11.23	24666786	24838328
DAPK3	1613	19	p13.3	3958452	3969826
DCBLD1	285761	6	q22.1	117803820	117891021
DCBLD2	131566	3	q12.1	98514785	98620533
DCHS1	8642	11	p15.4	6642556	6677085
DCHS2	54798	4	q31.3	155155527	155412930
DCX	1641	X	q23	110537007	110655603
DDIT4	54541	10	q22.1	74033678	74035794
DDIT4L	115265	4	q24	101107027	101111939
DDR2	4921	1	q23.3	162601163	162750237
DENND1A	57706	9	q33.3	126141933	126692431
DES	1674	2	q35	220283099	220291461
DGCR2	9993	22	q11.21	19023799	19109967
DGCR6	8214	22	q11.21	18893541	18901751
DLG1	1739	3	q29	196769431	197026171
DLG2	1740	11	q14.1	83166055	85338966
DLG4	1742	17	p13.1	7093209	7123369
DLGAP1	9229	18	p11.31	3496030	3880135
DLGAP3	58512	1	p34.3	35331037	35395186
DLGAP4	22839	20	q11.23	34894258	35157040
DLL3	10683	19	q13.2	39989557	39999118
DMBX1	127343	1	p33	46972669	46979898
DMXL2	23312	15	q21.2	51739908	51914967
DNER	92737	2	q36.3	230222345	230579274
DNM2	1785	19	p13.2	10824143	10942579
DNMBP	23268	10	q24.2	101635334	101769676
DOC2A	8448	16	p11.2	30016835	30022401
DOK7	285489	4	p16.3	3465033	3503200
DPP4	1803	2	q24.2	162848751	162931052
DPT	1805	1	q24.2	168664697	168698502
DPYSL5	56896	2	p23.3	27070615	27173219
DRGX	644168	10	q11.23	50572237	50603986
DRP2	1821	X	q22.1	100474758	100519486

DSC1	1823	18	q12.1	28709199	28742819
DSC2	1824	18	q12.1	28645940	28682388
DSC3	1825	18	q12.1	28570052	28622781
DSCAM	1826	21	q22.2	41382926	42219065
DSCAML1	57453	11	q23.3	117299044	117667974
DSG2	1829	18	q12.1	29078027	29128813
DSG3	1830	18	q12.1	29027758	29058665
DSG4	147409	18	q12.1	28956740	28993880
DST	667	6	p12.1	56322785	56819426
DTNA	1837	18	q12.1	32073254	32471808
DTNB	1838	2	p23.3	25600112	25896503
DTNBP1	84062	6	p22.3	15523032	15663289
EDIL3	10085	5	q14.3	83236373	83680611
EDN3	1908	20	q13.32	57875482	57901047
EFHD1	80303	2	q37.1	233470767	233547491
EFNA1	1942	1	q22	155099936	155107333
EFNB1	1947	X	q13.1	68048840	68061990
EFS	10278	14	q11.2	23825611	23834961
EGFL6	25975	X	p22.2	13587724	13651694
EGFLAM	133584	5	p13.2	38258511	38465123
EIF2B2	8892	14	q24.3	75469612	75476292
EMB	133418	5	q11.1	49692026	49739082
EMILIN1	11117	2	p23.3	27301435	27309271
EMILIN2	84034	18	p11.32	2847028	2914090
EMR1	2015	19	p13.3	6887582	6940463
EMX1	2016	2	p13.2	73143389	73161477
EMX2	2018	10	q26.11	119301956	119309057
EN1	2019	2	q14.2	119599766	119605759
EN2	2020	7	q36.3	155250824	155257526
ENAH	55740	1	q42.12	225677502	225840844
ENG	2022	9	q34.11	130577291	130617035
ENTPD1	953	10	q24.1	97454774	97637023
EPHA2	1969	1	p36.13	16450832	16482582
ERBB3	2065	12	q13.2	56473892	56497128
ERC2	26059	3	p14.3	55542336	56502391
EXOC4	60412	7	q33	132937829	133751342
F11R	50848	1	q23.3	160965001	161008752
F5	2153	1	q24.2	169483404	169555826
F8	2157	X	q28	154064063	154255215
FAIM2	23017	12	q13.12	50260680	50297720
FAT2	2196	5	q33.1	150883654	150948505

FAT3	120114	11	q14.3	92085262	92629636
FAT4	79633	4	q28.1	126237554	126414087
FBLIM1	54751	1	p36.21	16083102	16113089
FBLN5	10516	14	q32.12	92335758	92414046
FBLN7	129804	2	q13	112895962	112988430
FBXO45	200933	3	q29	196295482	196315930
FER	2241	5	q21.3	108083523	108532542
FERMT1	55612	20	p12.3	6055492	6104191
FERMT2	10979	14	q22.1	53323986	53417815
FERMT3	83706	11	q13.1	63974152	63991363
FEZ1	9638	11	q24.2	125315657	125366206
FGFR1	2260	8	p11.22	38268656	38326352
FLOT2	2319	17	q11.2	27206353	27224715
FLRT1	23769	11	q13.1	63871362	63886645
FLRT2	23768	14	q31.3	85996488	86094269
FLRT3	23767	20	p12.1	14303634	14318262
FN1	2335	2	q35	216225163	216300895
FPR2	2358	19	q13.41	52264183	52273760
FREM1	158326	9	p22.3	14737150	14910993
FREM2	341640	13	q13.3	39261173	39460074
FREM3	166752	4	q31.21	144498455	144621828
FXYD2	486	11	q23.3	117690790	117698807
FZD1	8321	7	q21.13	90893783	90898123
FZD10	11211	12	q24.33	130647032	130650284
FZD2	2535	17	q21.31	42634925	42636907
FZD3	7976	8	p21.1	28351731	28431775
FZD4	8322	11	q14.2	86656722	86666433
FZD5	7855	2	q33.3	208627310	208634287
FZD7	8324	2	q33.1	202899310	202903160
FZD8	8325	10	p11.21	35927177	35930362
GABBR2	9568	9	q22.33	101050391	101471479
GABRA1	2554	5	q34	161274197	161326975
GABRA2	2555	4	p12	46250444	46477247
GABRA3	2556	X	q28	151334706	151620337
GABRA4	2557	4	p12	46920917	46996424
GABRA5	2558	15	q12	27112058	27194349
GABRA6	2559	5	q34	160974069	161129599
GABRB1	2560	4	p12	46995740	47428461
GABRB2	2561	5	q34	160715436	160976050
GABRB3	2562	15	q12	26788693	27018924
GABRD	2563	1	p36.33	1950780	1962192

GABRE	2564	X	q28	151121596	151143152
GABRG1	2565	4	p12	46037786	46126098
GABRG2	2566	5	q34	161494546	161582542
GABRG3	2567	15	q12	27216429	27778373
GABRP	2568	5	q35.1	170190354	170241051
GABRQ	55879	X	q28	151806637	151825999
GABRR1	2569	6	q15	89887220	89940997
GABRR2	2570	6	q15	89967239	90025018
GAD1	2571	2	q31.1	171669723	171717661
GAD2	2572	10	p12.1	26505236	26593487
GALR2	8811	17	q25.1	74070884	74073573
GAP43	2596	3	q13.31	115342171	115440337
GAS7	8522	17	p13.1	9813926	10101868
GATA2	2624	3	q21.3	128198265	128212028
GDNF	2668	5	p13.2	37812779	37839788
GLI3	2737	7	p14.1	42000548	42277469
GLRA1	2741	5	q33.1	151202074	151304403
GLRA2	2742	X	p22.2	14547420	14749934
GLRA3	8001	4	q34.1	175558065	175750465
GLRA4	441509	X	q22.2	102962152	102983583
GLRB	2743	4	q32.1	157997209	158093242
GNAO1	2775	16	q12.2	56225302	56391354
GNE	10020	9	p13.3	36214430	36277041
GOPC	57120	6	q22.1	117639374	117923691
GP1BB	2812	22	q11.21	19710468	19712294
GP5	2814	3	q29	194115550	194119995
GP9	2815	3	q21.3	128779610	128781249
GPC2	221914	7	q22.1	99767229	99774995
GPHN	10243	14	q23.3	66974125	67648520
GPNMB	10457	7	p15.3	23275586	23314727
GPR56	9289	16	q21	57662419	57698944
GPRIN1	114787	5	q35.2	176022803	176037134
GRASP	160622	12	q13.13	52400724	52409648
GRIA1	2890	5	q33.2	152869175	153193240
GRIA2	2891	4	q32.1	158125334	158287227
GRIA4	2893	11	q22.3	105480800	105852819
GRID1	2894	10	q23.2	87359312	88126250
GRID2	2895	4	q22.1	93225550	94695707
GRID2IP	392862	7	p22.1	6537093	6591092
GRIK1	2897	21	q21.3	30909254	31312351
GRIK2	2898	6	q16.3	101846664	102517958

GRIK3	2899	1	p34.3	37261128	37499730
GRIK4	2900	11	q23.3	120530971	120857132
GRIK5	2901	19	q13.2	42502477	42569957
GRIN1	2902	9	q34.3	140032842	140063207
GRIN2A	2903	16	p13.2	9847261	10276611
GRIN2B	2904	12	p13.1	13714144	14133053
GRIN2C	2905	17	q25.1	72838162	72856966
GRIN2D	2906	19	q13.33	48898132	48948187
GRIN3A	116443	9	q31.1	104331635	104500862
GRIN3B	116444	19	p13.3	1000437	1009721
GSTP1	2950	11	q13.2	67351066	67354131
GSX2	170825	4	q12	54965690	54968672
HABP2	3026	10	q25.3	115312785	115349358
HAPLN1	1404	5	q14.3	82933624	83017432
HAPLN2	60484	1	q23.1	156589086	156595517
HAPLN3	145864	15	q26.1	89420519	89438857
HAPLN4	404037	19	p13.11	19366456	19373605
HAS1	3036	19	q13.41	52216365	52227237
HCN2	610	19	p13.3	589893	617157
HCN4	10021	15	q24.1	73612200	73661605
HCRT	3060	17	q21.2	40336078	40337470
HDAC2	3066	6	q21	114254192	114332472
HELT	391723	4	q35.1	185939995	185941958
HEPACAM	220296	11	q24.2	124789148	124806308
HES1	3280	3	q29	193853934	193856521
HES5	388585	1	p36.32	2460184	2461684
HHIP	64399	4	q31.21	145567173	145666423
HMGB1	3146	13	q12.3	31032884	31191734
HOMER1	9456	5	q14.1	78668459	78810040
HOMER2	9455	15	q25.2	83517738	83621473
HOMER3	9454	19	p13.11	19040010	19051983
HOXC8	3224	12	q13.13	54402890	54406547
HPCAL4	51440	1	p34.2	40144320	40157361
HSPB11	51668	1	p32.3	54387234	54411975
HSPG2	3339	1	p36.12	22148738	22263790
HTR3A	3359	11	q23.2	113845603	113861035
HTT	3064	4	p16.3	3076408	3245676
IBSP	3381	4	q22.1	88720733	88733074
ICA1	3382	7	p21.3	8152814	8302317
ICAM1	3383	19	p13.2	10381517	10397291
ID3	3399	1	p36.12	23884409	23886285

IGF1R	3480	15	q26.3	99192200	99507759
IGFALS	3483	16	p13.3	1840414	1843734
IGFBP3	3486	7	p12.3	45951951	45961473
IGFBP7	3490	4	q12	57896939	57976551
IGSF11	152404	3	q13.32	118619404	118864915
IGSF9	57549	1	q23.2	159896829	159915394
IHH	3549	2	q35	219919146	219925189
IL1RAPL2	26280	X	q22.3	103810996	105011822
IL2	3558	4	q27	123372625	123377880
IL6	3569	7	p15.3	22765503	22771621
INPP5D	3635	2	q37.1	233924677	234116549
INPPL1	3636	11	q13.4	71935825	71950149
INSR	3643	19	p13.2	7112266	7294011
ISL1	3670	5	q11.1	50678958	50690555
ISLR	3671	15	q24.1	74466012	74469213
ITGA1	3672	5	q11.2	52083730	52252327
ITGA10	8515	1	q21.1	145524891	145543868
ITGA2	3673	5	q11.2	52285156	52390609
ITGA2B	3674	17	q21.31	42449550	42466873
ITGA3	3675	17	q21.33	48133332	48167848
ITGA4	3676	2	q31.3	182321619	182400914
ITGA5	3678	12	q13.13	54789047	54813050
ITGA9	3680	3	p22.2	37493606	37865005
ITGAE	3682	17	p13.2	3617923	3704544
ITGAL	3683	16	p11.2	30483983	30534505
ITGAM	3684	16	p11.2	31271288	31344213
ITGAV	3685	2	q32.1	187454792	187545628
ITGAX	3687	16	p11.2	31366509	31394318
ITGB1	3688	10	p11.22	33189247	33294720
ITGB2	3689	21	q22.3	46305868	46351904
ITGB3	3690	17	q21.32	45331208	45390077
ITGB3BP	23421	1	p31.3	63906441	64059392
ITGB4	3691	17	q25.1	73717516	73753898
ITGB5	3693	3	q21.2	124480795	124606674
ITGB6	3694	2	q24.2	160956177	161128399
ITGB7	3695	12	q13.13	53585110	53601000
ITGB8	3696	7	p21.1	20370725	20450419
ITGBL1	9358	13	q33.1	102104966	102371145
ITM2C	81618	2	q37.1	231729313	231743963
ITSN1	6453	21	q22.11	35014706	35272165
IZUMO1	284359	19	q13.33	49244145	49250166

JARID2	3720	6	p22.3	15246527	15522253
JRKL	8690	11	q21	96123158	96126727
JUB	84962	14	q11.2	23440383	23451851
JUP	3728	17	q21.2	39910856	39943183
KAL1	3730	X	p22.31	8496915	8700227
KCTD12	115207	13	q22.3	77454312	77460540
KCTD16	57528	5	q31.3	143550396	143856944
KCTD8	386617	4	p13	44175926	44450824
KIAA0195	9772	17	q25.1	73452664	73496530
KIAA1919	91749	6	q21	111580551	111592370
KIF26A	26153	14	q32.33	104605060	104647235
KIRREL2	84063	19	q13.12	36347810	36358048
KITLG	4254	12	q21.32	88886566	88974238
KLHL17	339451	1	p36.33	895967	901095
KLK6	5653	19	q13.41	51461888	51472929
L1CAM	3897	X	q28	153126969	153174677
LAMA1	284217	18	p11.23	6941743	7117813
LAMA2	3908	6	q22.33	129204286	129837714
LAMA3	3909	18	q11.2	21269562	21535030
LAMA4	3910	6	q21	112429963	112576141
LAMA5	3911	20	q13.33	60883011	60942368
LAMB1	3912	7	q31.1	107564244	107643804
LAMB2	3913	3	p21.31	49158547	49170599
LAMB3	3914	1	q32.2	209788220	209825820
LAMB4	22798	7	q31.1	107663993	107770801
LAMC1	3915	1	q25.3	182992597	183114727
LAMC2	3918	1	q25.3	183155373	183214035
LAMC3	10319	9	q34.12	133884469	133969860
LDB1	8861	10	q24.32	103867317	103880210
LEF1	51176	4	q25	108968701	109089578
LGALS3BP	3959	17	q25.3	76967337	76976061
LGALS4	3960	19	q13.2	39292312	39303740
LGI1	9211	10	q23.33	95517642	95557916
LGI3	203190	8	p21.3	22004338	22014597
LIG4	3981	13	q33.3	108859794	108870716
LIN7A	8825	12	q21.31	81191175	81331694
LIN7B	64130	19	q13.33	49617581	49621717
LIN7C	55327	11	p14.1	27516124	27528303
LINGO1	84894	15	q24.3	77905369	77924709
LMLN	89782	3	q29	197687071	197770591
LMX1A	4009	1	q23.3	165171104	165325952

LMX1B	4010	9	q33.3	129376722	129463311
LOXL2	4017	8	p21.3	23154702	23282841
LPP	4026	3	q27.3	187871072	188608460
LPXN	9404	11	q12.1	58294349	58343390
LRFN1	57622	19	q13.2	39797208	39805976
LRFN2	57497	6	p21.1	40359325	40555204
LRFN3	79414	19	q13.12	36427750	36436084
LRRC4	64101	7	q32.1	127667124	127672160
LRRC4B	94030	19	q13.33	51020149	51071302
LRRN2	10446	1	q32.1	204586298	204654861
LSAMP	4045	3	q13.31	115528641	116858236
LY6D	8581	8	q24.3	143866296	143868008
LY9	4063	1	q23.3	160765896	160798045
LYN	4067	8	q12.1	56792372	56923939
LYVE1	10894	11	p15.4	10579413	10590365
LZTS1	11178	8	p21.3	20103676	20161474
MADCAM1	8174	19	p13.3	496454	505340
MAEA	10296	4	p16.3	1283639	1333935
MAG	4099	19	q13.12	35783028	35804707
MAGI2	9863	7	q21.11	77646393	79082890
MAL	4118	2	q11.1	95691422	95719737
MAP1B	4131	5	q13.2	71403061	71505395
MAP1S	55201	19	p13.11	17830291	17845322
MAP2K1	5604	15	q22.31	66679155	66783882
MBP	4155	18	q23	74690783	74845639
MCAM	4162	11	q23.3	119179234	119187840
MCOLN1	57192	19	p13.2	7587514	7598868
MEGF10	84466	5	q23.2	126626523	126801429
MFAP4	4239	17	p11.2	19286755	19290553
MFGE8	4240	15	q26.1	89441914	89456685
MMRN1	22915	4	q22.1	90800683	90875780
MNX1	3110	7	q36.3	156786745	156803239
MPZL2	10205	11	q23.3	118124137	118135009
MPZL3	196264	11	q23.3	118097409	118123035
MSLN	10232	16	p13.3	812594	818865
MTPN	136319	7	q33	135611509	135662107
MTSS1	9788	8	q24.13	125563031	125740730
MUC4	4585	3	q29	195473636	195539148
MYBPC1	4604	12	q23.2	101988747	102079658
MYBPC2	4606	19	q13.33	50936160	50969583
MYBPH	4608	1	q32.1	203136939	203144941

MYH9	4627	22	q12.3	36677327	36784063
MYO7A	4647	11	q13.5	76839310	76926286
MYRIP	25924	3	p22.1	39850405	40301812
NAPA	8775	19	q13.33	47990891	48018497
NCAM1	4684	11	q23.2	113003650	113149133
NCAM2	4685	21	q21.1	22370633	22915650
NCAN	1463	19	p13.11	19322782	19363061
NCDN	23154	1	p34.3	36023074	36032875
NCKAP1	10787	2	q32.1	183789605	183903586
NCS1	23413	9	q34.11	132934857	132999583
NDEL1	81565	17	p13.1	8339179	8383942
NDN	4692	15	q11.2	23930565	23932450
NDUFA9	4704	12	p13.32	4758283	4796397
NEDD4	4734	15	q21.3	56119120	56286053
NEDD4L	23327	18	q21.31	55711619	56068772
NEDD9	4739	6	p24.2	11183531	11382581
NEFM	4741	8	p21.2	24770525	24776607
NEGR1	257194	1	p31.1	71868625	72748417
NELL1	4745	11	p15.1	20691117	21597227
NELL2	4753	12	q12	44902058	45278334
NEO1	4756	15	q24.1	73344875	73597546
NES	10763	1	q23.1	156638555	156647189
NEUROD1	4760	2	q31.3	182537815	182545603
NEUROG1	4762	5	q31.1	134869991	134871639
NEUROG2	63973	4	q25	113434672	113437328
NEUROG3	50674	10	q22.1	71331454	71332994
NFASC	23114	1	q32.1	204797779	204991950
NGF	4803	1	p13.2	115828539	115880857
NGFR	4804	17	q21.33	47572655	47592379
NGRN	51335	15	q26.1	90808891	90816463
NHEDC1	100134103	4	q24	103806205	103940896
NHEDC2	133308	4	q24	103941025	104006986
NHEJ1	79840	2	q35	219940039	220025587
NHLH1	4807	1	q23.2	160336857	160342638
NHLH2	4808	1	p13.1	116378998	116386538
NID1	4811	1	q42.3	236139130	236228481
NID2	22795	14	q22.1	52471521	52536545
NINJ1	4814	9	q22.31	95883771	95896570
NINJ2	4815	12	p13.33	673462	772907
NLGN4Y	22829	Y	q11.221	16634518	16957530
NLRP12	91662	19	q13.42	54296857	54327648

NME2	4831	17	q21.33	49230951	49249108
NMUR2	56923	5	q33.1	151771093	151812929
NNAT	4826	20	q11.23	36149617	36152092
NOG	9241	17	q22	54671060	54672951
NOTCH1	4851	9	q34.3	139388896	139440314
NPAS1	4861	19	q13.32	47523101	47549033
NPAS2	4862	2	q11.2	101436614	101613291
NPHS1	4868	19	q13.12	36316283	36342739
NPNT	255743	4	q24	106815932	106925184
NPTN	27020	15	q24.1	73852355	73925756
NPTX1	4884	17	q25.3	78440633	78450404
NR2C2AP	126382	19	p13.11	19312226	19314238
NRCAM	4897	7	q31.1	107788082	108097161
NRG1	3084	8	p12	31496902	32622294
NRP1	8829	10	p11.22	33466420	33625190
NRP2	8828	2	q33.3	206546714	206662857
NRTN	4902	19	p13.3	5823818	5828334
NRXN2	9379	11	q13.1	64373646	64490660
NRXN3	9369	14	q24.3	79081275	80330758
NTM	50863	11	q25	131240373	132206716
OLFM4	10562	13	q14.3	53602830	53626196
OLR1	4973	12	p13.2	10310904	10324790
OMD	4958	9	q22.31	95176527	95186743
OMG	4974	17	q11.2	29621665	29624349
OPCML	4978	11	q25	132284871	133402219
OPHN1	4983	X	q12	67262186	67653755
OTOF	9381	2	p23.3	26680071	26781566
OTX2	5015	14	q22.3	57267426	57277187
P2RX3	5024	11	q12.1	57105949	57137549
P2RX4	5025	12	q24.31	121647664	121671907
P2RX7	5027	12	q24.31	121570678	121623856
PAK1IP1	55003	6	p24.2	10694928	10710015
PAM	5066	5	q21.1	102089685	102366809
PANX1	24145	11	q21	93862094	93915133
PARVA	55742	11	p15.3	12399146	12551410
PARVB	29780	22	q13.31	44395091	44565106
PARVG	64098	22	q13.31	44568836	44615413
PAX6	5080	11	p13	31806340	31839509
PCDH1	5097	5	q31.3	141232938	141258811
PCDH11X	27328	X	q21.31	91034260	91878229
PCDH11Y	83259	Y	p11.2	4868267	5610265

PCDH12	51294	5	q31.3	141323150	141349304
PCDH15	65217	10	q21.1	55562531	57387702
PCDH17	27253	13	q21.1	58205789	58303445
PCDH18	54510	4	q28.3	138440072	138453648
PCDH19	57526	X	q22.1	99546642	99665271
PCDH20	64881	13	q21.2	61983821	62002220
PCDH7	5099	4	p15.1	30722037	31144728
PCDH8	5100	13	q14.3	53418109	53422775
PCDHA1	56147	5	q31.3	140165721	140168446
PCDHA10	56139	5	q31.3	140235468	140391929
PCDHA11	56138	5	q31.3	140247722	140391929
PCDHA12	56137	5	q31.3	140254887	140391929
PCDHA13	56136	5	q31.3	140261793	140391929
PCDHA2	56146	5	q31.3	140174444	140185220
PCDHA4	56144	5	q31.3	140186514	140189252
PCDHA5	56143	5	q31.3	140201189	140203862
PCDHA6	56142	5	q31.3	140206816	140391929
PCDHA7	56141	5	q31.3	140213816	140216711
PCDHA9	9752	5	q31.3	140227357	140233515
PCDHAC1	56135	5	q31.3	140306457	140391929
PCDHAC2	56134	5	q31.3	140345820	140391936
PCDHB1	29930	5	q31.3	140430961	140433547
PCDHB10	56126	5	q31.3	140571952	140575213
PCDHB11	56125	5	q31.3	140579298	140581769
PCDHB12	56124	5	q31.3	140588291	140591696
PCDHB13	56123	5	q31.3	140593538	140596290
PCDHB14	56122	5	q31.3	140602946	140605869
PCDHB15	56121	5	q31.3	140624981	140627742
PCDHB16	57717	5	q31.3	140560980	140565793
PCDHB2	56133	5	q31.3	140474237	140476962
PCDHB3	56132	5	q31.3	140480234	140483406
PCDHB4	56131	5	q31.3	140501581	140505201
PCDHB5	26167	5	q31.3	140514810	140517703
PCDHB6	56130	5	q31.3	140529839	140532868
PCDHB7	56129	5	q31.3	140552335	140555385
PCDHB8	56128	5	q31.3	140557371	140560081
PCDHGA1	56114	5	q31.3	140710204	140892546
PCDHGA10	56106	5	q31.3	140792743	140892546
PCDHGA11	56105	5	q31.3	140800762	140891835
PCDHGA12	26025	5	q31.3	140810185	140892546
PCDHGA2	56113	5	q31.3	140718488	140892546

PCDHGA3	56112	5	q31.3	140723601	140892546
PCDHGA5	56110	5	q31.3	140743898	140892546
PCDHGA6	56109	5	q31.3	140753651	140892546
PCDHGA7	56108	5	q31.3	140762467	140892546
PCDHGA8	9708	5	q31.3	140772381	140892546
PCDHGB1	56104	5	q31.3	140729828	140892546
PCDHGB2	56103	5	q31.3	140739703	140892546
PCDHGB4	8641	5	q31.3	140767452	140892546
PCDHGB6	56100	5	q31.3	140787770	140892546
PCDHGB7	56099	5	q31.3	140797427	140892546
PCDHGC4	56098	5	q31.3	140864741	140892546
PCDHGC5	56097	5	q31.3	140868808	140892546
PCLO	27445	7	q21.11	82383321	82792246
PCP4	5121	21	q22.2	41239243	41301322
PCSK1	5122	5	q15	95726119	95769847
PCSK9	255738	1	p32.3	55505220	55530525
PDGFC	56034	4	q32.1	157681606	157892546
PDLIM5	10611	4	q22.3	95373037	95589377
PDPN	10630	1	p36.21	13909960	13944452
PDXK	8566	21	q22.3	45138975	45194151
PDZD2	23037	5	p13.3	31639517	32111037
PERP	64065	6	q23.3	138411923	138428648
PGM5	5239	9	q21.11	70971815	71145977
PHACTR1	221692	6	p24.1	12717893	13288075
PHGDH	26227	1	p12	120202421	120286838
PHOX2B	8929	4	p13	41746099	41750987
PIAS3	10401	1	q21.1	145575233	145586546
PICK1	9463	22	q13.1	38452318	38471708
PIGT	51604	20	q13.12	44044717	44054884
PJA2	9867	5	q21.3	108670410	108745695
PKD2	5311	4	q22.1	88928820	88998929
PKD2L1	9033	10	q24.31	102047903	102090243
PKP1	5317	1	q32.1	201252580	201302121
PKP3	11187	11	p15.5	394217	404908
PKP4	8502	2	q24.1	159313476	159539391
PLAT	5327	8	p11.21	42032236	42065242
PLD2	5338	17	p13.2	4710391	4726727
PLXNC1	10154	12	q22	94542748	94701444
PNMA1	9240	14	q24.3	74178494	74181128
PNN	5411	14	q21.1	39644425	39652422
PODXL2	50512	3	q21.3	127348024	127391652

POSTN	10631	13	q13.3	38136722	38172981
POU3F2	5454	6	q16.1	99282611	99286660
POU3F3	5455	2	q12.1	105471969	105473471
POU4F1	5457	13	q31.1	79173227	79177695
POU4F2	5458	4	q31.22	147560045	147563626
POU6F2	11281	7	p14.1	39017598	39532694
PPFIBP1	8496	12	p11.23	27677045	27848496
PPP1R9A	55607	7	q21.3	94536514	94925725
PRDM12	59335	9	q34.12	133539981	133558368
PRDM16	63976	1	p36.32	2985732	3355185
PRDM6	93166	5	q23.2	122424816	122523745
PRDM8	56978	4	q21.21	81105033	81125483
PRIMA1	145270	14	q32.12	94184644	94254827
PRKCQ	5588	10	p14	6469105	6622263
PROP1	5626	5	q35.3	177419236	177423243
PRPH2	5961	6	p21.1	42664340	42690312
PRR7	80758	5	q35.3	176873446	176883283
PRSS12	8492	4	q26	119201193	119274158
PSD	5662	10	q24.32	104162376	104181296
PSEN1	5663	14	q24.2	73603155	73687109
PSPN	5623	19	p13.3	6375305	6375860
PSTPIP1	9051	15	q24.3	77287465	77329671
PTEN	5728	10	q23.31	89622870	89731687
PTK2B	2185	8	p21.2	27168999	27316903
PTK7	5754	6	p21.1	43044006	43129457
PTPRF	5792	1	p34.2	43990858	44089343
PTPRM	5797	18	p11.23	7567817	8406859
PTPRR	5801	12	q15	71031862	71314586
PTPRS	5802	19	p13.3	5205519	5340814
PTPRT	11122	20	q13.11	40701392	41818610
PTPRU	10076	1	p35.3	29563028	29653313
PTPRZ1	5803	7	q31.32	121513143	121702090
PTS	5805	11	q23.1	112097088	112104691
PVRL1	5818	11	q23.3	119508808	119599435
PVRL2	5819	19	q13.32	45349393	45392485
PVRL3	25945	3	q13.13	110788918	110994410
PVRL4	81607	1	q23.3	161040785	161059389
PXN	5829	12	q24.31	120648251	120703563
RAB13	100131294	1	q21.3	153954127	153958834
RABAC1	10567	19	q13.2	42460838	42463528
RAC1	5879	7	p22.1	6414154	6443608

RAC3	5881	17	q25.3	79989532	79992077
RADIL	55698	7	p22.1	4836686	4923350
RAPSN	5913	11	p11.2	47459308	47470730
RARB	5915	3	p24.2	25215823	25639423
RASEF	158158	9	q21.32	85594500	85678092
RASGRF1	5923	15	q25.1	79252289	79383215
RASGRP2	10235	11	q13.1	64494383	64512928
RBPJ	3516	4	p15.2	26165077	26436541
RCAN1	1827	21	q22.12	35885440	35987441
RCAN2	10231	6	p12.3	46188475	46459709
RELN	5649	7	q22.1	103112231	103629963
RGMB	285704	5	q15	98104354	98134347
RGS1	5996	1	q31.2	192544857	192549161
RGS11	8786	16	p13.3	318300	325980
RGS12	6002	4	p16.3	3294755	3441640
RGS13	6003	1	q31.2	192605275	192629390
RGS14	10636	5	q35.3	176784838	176799602
RGS16	6004	1	q25.3	182567758	182573543
RGS17	26575	6	q25.2	153331857	153452384
RGS18	64407	1	q31.2	192127587	192154945
RGS19	10287	20	q13.33	62704534	62711323
RGS2	5997	1	q31.2	192778169	192781403
RGS20	8601	8	q11.23	54764368	54871863
RGS21	431704	1	q31.2	192286122	192336415
RGS22	26166	8	q22.2	100973164	101143496
RGS3	5998	9	q32	116207011	116360018
RGS4	5999	1	q23.3	163038396	163046592
RGS5	8490	1	q23.3	163080911	163187426
RGS6	9628	14	q24.2	72431509	73033237
RGS7BP	401190	5	q12.3	63802084	63908139
RGS8	85397	1	q25.3	182615239	182653711
RGS9	8787	17	q24.1	63133549	63223819
RGS9BP	388531	19	q13.11	33166313	33169206
RHOA	387	3	p21.31	49396578	49450431
RHOB	388	2	p24.1	20646835	20649206
RIMBP2	23504	12	q24.33	130880682	131002410
RIMS1	22999	6	q13	72596406	73112845
RIMS4	140730	20	q13.12	43380449	43438979
RND3	390	2	q23.3	151324709	151395525
RNF103	7844	2	p11.2	86830516	86850989
ROBO1	6091	3	p12.2	78646390	79816965

ROBO2	6092	3	p12.3	75955846	77699115
ROM1	6094	11	q12.3	62380213	62382590
RPH3A	22895	12	q24.13	113229549	113336679
RPS6KA3	6197	X	p22.12	20168029	20285218
RPS6KA6	27330	X	q21.1	83318984	83442915
RPS6KB1	6198	17	q23.1	57970443	58027925
RPSA	3921	3	p22.1	39448180	39454030
RS1	6247	X	p22.13	18658030	18690229
RTN1	6252	14	q23.1	60062694	60337684
RUNX1	861	21	q22.12	36160098	37357047
RUNX3	864	1	p36.11	25226002	25291612
S100B	6285	21	q22.3	48018875	48025121
S1PR1	1901	1	p21.2	101702444	101707074
SAMD4A	23034	14	q22.2	55034637	55255845
SCAI	286205	9	q33.3	127704887	127905785
SCAMP1	9522	5	q14.1	77656407	77774894
SCAMP5	192683	15	q24.2	75287901	75313836
SCARB1	949	12	q24.31	125262186	125348519
SCARB2	950	4	q21.1	77079890	77135046
SCARF1	8578	17	p13.3	1537152	1549047
SCN10A	6336	3	p22.2	38738293	38835501
SCN11A	11280	3	p22.2	38887260	38992052
SCN1B	6324	19	q13.12	35521534	35531352
SCN2B	6327	11	q23.3	118036187	118047241
SCN3A	6328	2	q24.3	165944032	166060577
SCN3B	55800	11	q24.1	123499897	123525315
SCN4A	6329	17	q23.3	62015914	62050278
SCN4B	6330	11	q23.3	118004092	118023630
SCN5A	6331	3	p22.2	38589548	38691164
SCN7A	6332	2	q24.3	167260083	167350757
SCN8A	6334	12	q13.13	51985020	52202297
SCN9A	6335	2	q24.3	167051695	167232503
SCNN1A	6337	12	p13.31	6456015	6484715
SCNN1B	6338	16	p12.2	23313591	23392620
SCNN1D	6339	1	p36.33	1215852	1227409
SCNN1G	6340	16	p12.2	23194036	23228204
SDC2	6383	8	q22.1	97505579	97624000
SDC3	9672	1	p35.2	31342313	31381608
SDK1	221935	7	p22.2	3341080	4308632
SDK2	54549	17	q25.1	71330523	71640227
SEBOX	7448	17	q11.2	26691290	26697717

SELE	6401	1	q24.2	169691781	169703220
SELL	6402	1	q24.2	169659808	169680839
SELP	6403	1	q24.2	169558037	169599380
SELPLG	6404	12	q24.11	109015686	109027670
SEMA4C	54910	2	q11.2	97525453	97536494
SEMA4D	10507	9	q22.2	91975706	92112888
SEMA4F	10505	2	p13.1	74881355	74909186
SEMA5A	9037	5	p15.31	9035138	9546187
SEPT11	55752	4	q21.1	77870856	77961537
SEPT2	4735	2	q37.3	242254515	242293442
SEPT3	55964	22	q13.2	42372276	42394225
SERPINI1	5274	3	q26.1	167453031	167543356
SFXN1	94081	5	q35.2	174904065	174956745
SFXN2	118980	10	q24.32	104474297	104498951
SFXN3	81855	10	q24.31	102790991	102800998
SFXN4	119559	10	q26.11	120900279	120925189
SFXN5	94097	2	p13.2	73169165	73302747
SGK1	6446	6	q23.2	134490387	134639196
SH3GL1	6455	19	p13.3	4360370	4400496
SH3GL2	6456	9	p22.2	17578953	17797127
SH3GL3	6457	15	q25.2	84115980	84287495
SH3KBP1	30011	X	p22.12	19552083	19905719
SHANK1	50944	19	q13.33	51165084	51222707
SHC1	6464	1	q21.3	154934774	154946959
SHC3	53358	9	q22.1	91628060	91793682
SHC4	399694	15	q21.1	49115932	49255641
SHH	6469	7	q36.3	155592680	155604967
SHISA9	729993	16	p13.12	12995455	13329566
SIGLEC11	114132	19	q13.33	50412758	50464429
SIGLEC12	89790	19	q13.41	51913276	52005001
SIGLEC14	8778	19	q13.41	52114781	52150151
SIGLEC6	946	19	q13.41	52020951	52035110
SIGLEC7	27036	19	q13.41	51645558	51656783
SIGLEC8	27181	19	q13.41	51954101	51961708
SIGLEC9	27180	19	q13.41	51628165	51633566
SIPA1L1	26037	14	q24.2	71996042	72206118
SIRPA	140885	20	p13	1874813	1920543
SIRPG	55423	20	p13	1609798	1638425
SLAMF7	57823	1	q23.3	160709037	160724611
SLC10A1	6554	14	q24.2	70242589	70264006
SLC10A2	6555	13	q33.1	103696350	103719196

SLC10A3	8273	X	q28	153715645	153719016
SLC10A4	201780	4	p11	48485360	48491213
SLC10A5	347051	8	q21.13	82605842	82608409
SLC10A6	345274	4	q21.3	87744621	87770416
SLC10A7	84068	4	q31.22	147175127	147443123
SLC12A1	6557	15	q21.1	48498498	48596275
SLC12A2	6558	5	q23.3	127419458	127525380
SLC12A3	6559	16	q13	56899119	56949760
SLC12A4	6560	16	q22.1	67978230	68002555
SLC12A5	57468	20	q13.12	44650329	44688789
SLC12A6	9990	15	q14	34522197	34630265
SLC12A7	10723	5	p15.33	1050491	1112172
SLC13A1	6561	7	q31.32	122753585	122840040
SLC13A2	9058	17	q11.2	26800311	26824799
SLC13A3	64849	20	q13.12	45186463	45304714
SLC13A4	26266	7	q33	135365985	135412952
SLC13A5	284111	17	p13.1	6588032	6616740
SLC17A1	6568	6	p22.2	25783125	25832287
SLC17A3	10786	6	p22.2	25833294	25882514
SLC17A4	10050	6	p22.2	25754927	25781419
SLC17A6	57084	11	p14.3	22359667	22401044
SLC17A7	57030	19	q13.33	49932656	49944808
SLC17A8	246213	12	q23.1	100750857	100815837
SLC1A2	6506	11	p13	35272753	35441610
SLC1A3	6507	5	p13.2	36606457	36688436
SLC20A1	6574	2	q13	113403434	113421404
SLC20A2	6575	8	p11.21	42273993	42397069
SLC22A1	6580	6	q25.3	160542805	160579750
SLC22A2	6582	6	q25.3	160592093	160698670
SLC22A4	6583	5	q31.1	131630136	131679899
SLC22A5	6584	5	q31.1	131705401	131731306
SLC23A1	9963	5	q31.2	138702885	138720242
SLC23A2	9962	20	p13	4833002	4990939
SLC24A2	25769	9	p22.1	19515978	19786926
SLC24A3	57419	20	p11.23	19193290	19703545
SLC24A4	123041	14	q32.12	92788925	92962595
SLC24A5	283652	15	q21.1	48413169	48434869
SLC24A6	80024	12	q24.13	113736572	113772925
SLC30A1	7779	1	q32.3	211744910	211752065
SLC30A10	55532	1	q41	219858769	220131989
SLC30A2	7780	1	p36.11	26363743	26372624

SLC30A3	7781	2	p23.3	27476552	27498685
SLC30A4	7782	15	q21.1	45771809	45815005
SLC30A5	64924	5	q13.1	68389473	68426896
SLC30A6	55676	2	p22.3	32390933	32449448
SLC30A7	148867	1	p21.2	101361632	101447309
SLC30A8	169026	8	q24.11	117962512	118188950
SLC30A9	10463	4	p13	41992489	42089551
SLC32A1	140679	20	q11.23	37353105	37358015
SLC34A1	6569	5	q35.3	176806236	176825849
SLC34A2	10568	4	p15.2	25656923	25680370
SLC34A3	142680	9	q34.3	140125385	140131006
SLC38A1	81539	12	q13.11	46576843	46662780
SLC38A10	124565	17	q25.3	79218801	79269096
SLC38A2	54407	12	q13.11	46751972	46766645
SLC38A4	55089	12	q13.11	47158546	47219780
SLC38A6	145389	14	q23.1	61447880	61550450
SLC38A7	55238	16	q21	58699013	58718674
SLC38A8	146167	16	q23.3	84043389	84075762
SLC38A9	153129	5	q11.2	54921673	55069022
SLC41A1	254428	1	q32.1	205758221	205782876
SLC41A2	84102	12	q23.3	105197276	105352522
SLC41A3	54946	3	q21.3	125725198	125820404
SLC4A11	83959	20	p13	3208063	3219855
SLC4A4	8671	4	q13.3	72053003	72437804
SLC4A5	57835	2	p13.1	74443369	74570541
SLC4A7	9497	3	p24.1	27414214	27525911
SLC4A8	9498	12	q13.13	51796945	51902980
SLC5A1	6523	22	q12.3	32439019	32509016
SLC5A10	125206	17	p11.2	18853989	18924004
SLC5A11	115584	16	p12.1	24857162	24922947
SLC5A12	159963	11	p14.2	26688566	26744973
SLC5A2	6524	16	p11.2	31494439	31502091
SLC5A3	6526	21	q22.11	35445870	35478559
SLC5A4	6527	22	q12.3	32614465	32651328
SLC5A5	6528	19	p13.11	17982782	18005982
SLC5A6	8884	2	p23.3	27422455	27435826
SLC5A7	60482	2	q12.3	108602979	108630450
SLC5A8	160728	12	q23.2	101549566	101603989
SLC5A9	200010	1	p33	48688357	48714316
SLC8A1	6546	2	p22.1	40339286	40838193
SLC8A2	6543	19	q13.32	47931279	47975307

SLC8A3	6547	14	q24.2	70510934	70655787
SLC9A1	6548	1	p36.11	27425306	27493472
SLC9A10	285335	3	q13.2	111859734	112013105
SLC9A11	284525	1	q25.1	173469603	173572233
SLC9A2	6549	2	q12.1	103236166	103327777
SLC9A3	6550	5	p15.33	473425	524447
SLC9A4	389015	2	q12.1	103089762	103150431
SLC9A5	6553	16	q22.1	67282853	67306093
SLC9A6	10479	X	q26.3	135067598	135129423
SLC9A7	84679	X	p11.23	46464753	46618490
SLC9A8	23315	20	q13.13	48429250	48508779
SLURP1	57152	8	q24.3	143822362	143823829
SMARCA1	6594	X	q25	128580480	128657477
SMO	6608	7	q32.1	128828713	128853386
SNAP23	8773	15	q15.1	42787801	42825259
SNAP25	6616	20	p12.2	10199478	10288066
SNAP29	9342	22	q11.21	21213271	21245502
SNAPIN	23557	1	q21.3	153631130	153634306
SNPH	9751	20	p13	1246960	1289972
SNTB1	6641	8	q24.12	121547985	121825513
SNTB2	6645	16	q22.1	69221032	69342955
SNTG2	54221	2	p25.3	946554	1371385
SOCS1	8651	16	p13.13	11348262	11350036
SOCS2	8835	12	q22	93963598	93969978
SOCS3	9021	17	q25.3	76352864	76356158
SOCS4	122809	14	q22.2	55493844	55516206
SOCS5	9655	2	p21	46926091	46990268
SOCS6	9306	18	q22.2	67956168	67997436
SOCS7	30837	17	q12	36508005	36556015
SORBS3	10174	8	p21.3	22402499	22433301
SOX3	6658	X	q27.1	139585152	139587225
SPACA4	171169	19	q13.33	49110000	49110970
SPAM1	6677	7	q31.32	123565291	123611468
SPARCL1	8404	4	q22.1	88394487	88452213
SPOCK1	6695	5	q31.2	136310987	136934068
SPON1	10418	11	p15.2	13984452	14287233
SPON2	10417	4	p16.3	1160720	1202750
SPP1	6696	4	q22.1	88896802	88904562
SRCIN1	80725	17	q12	36686259	36762183
SRPX	8406	X	p11.4	38008592	38080696
SSPN	8082	12	p12.1	26348269	26387706

SSPO	23145	7	q36.1	149473131	149531068
SSX2IP	117178	1	p22.3	85109390	85156486
STAB1	23166	3	p21.1	52529354	52558511
STAB2	55576	12	q23.3	103981051	104160505
STIM1	6786	11	p15.4	3876932	4114439
STMN2	11075	8	q21.13	80523049	80578397
STMN3	50861	20	q13.33	62271061	62284780
STX1A	6804	7	q11.23	73113536	73134002
STX3	6809	11	q12.1	59522532	59570176
STXBP5	134957	6	q24.3	147525508	147708707
SUSD5	26032	3	p22.3	33191537	33260707
SV2B	9899	15	q26.1	91643515	91844539
SV2C	22987	5	q13.3	75378997	75649764
SVEP1	79987	9	q31.3	113127531	113342160
SVOP	55530	12	q24.11	109304658	109372440
SYMPK	8189	19	q13.32	46318693	46366548
SYN1	6853	X	p11.23	47431303	47479252
SYN3	8224	22	q12.3	32908539	33454358
SYNC	81493	1	p35.1	33145507	33169197
SYNGR1	9145	22	q13.1	39745930	39781593
SYNGR3	9143	16	p13.3	2039946	2044276
SYNPO	11346	5	q33.1	149980642	150038782
SYNPR	132204	3	p14.2	63213991	63602597
SYP	6855	X	p11.23	49044269	49056718
SYT1	6857	12	q21.2	79257773	79845787
SYT10	341359	12	p11.1	33528360	33592754
SYT11	23208	1	q22	155829300	155854990
SYT12	91683	11	q13.2	66790891	66818329
SYT2	127833	1	q32.1	202559724	202679545
SYT3	84258	19	q13.33	51125236	51141302
SYT4	6860	18	q12.3	40847859	40857615
SYT5	6861	19	q13.42	55684469	55691720
SYT6	148281	1	p13.2	114631914	114696541
SYT7	9066	11	q12.2	61282793	61348298
SYT9	143425	11	p15.4	7273181	7490268
TACC2	10579	10	q26.13	123748689	124014060
TACC3	10460	4	p16.3	1723227	1746898
TAGLN3	29114	3	q13.2	111717511	111732734
TANC1	85461	2	q24.2	159825146	160089170
TECTA	7007	11	q23.3	120971882	121062202
TGFB1I1	7041	16	p11.2	31483394	31489281

TGFBI	7045	5	q31.1	135364584	135399507
THBS1	7057	15	q14	39873127	39891119
THBS2	7058	6	q27	169615875	169654139
THBS3	7059	1	q22	155165379	155178842
THBS4	7060	5	q14.1	79287134	79379110
TIMP3	7078	22	q12.3	33197687	33259030
TIMP4	7079	3	p25.2	12194551	12200851
TINAG	27283	6	p12.1	54172657	54254950
TLN1	7094	9	p13.3	35696945	35732392
TLN2	83660	15	q22.2	62939477	63136830
TLX1	3195	10	q24.31	102890262	102897546
TLX3	30012	5	q35.1	170736288	170739138
TMCO3	55002	13	q34	114145308	114204542
TMEM8A	58986	16	p13.3	420773	437113
TMEM8B	51754	9	p13.3	35814448	35854844
TNC	3371	9	q33.1	117782806	117880486
TNFAIP6	7130	2	q23.3	152214106	152236560
TNFRSF12A	51330	16	p13.3	3070313	3072384
TNR	7143	1	q25.1	175291935	175712906
TP53	7157	17	p13.1	7565257	7590856
TP53BP2	7159	1	q41	223967596	224033674
TPBG	7162	6	q14.1	83073348	83080545
TPRG1L	127262	1	p36.32	3541566	3546691
TRAPPC10	7109	21	q22.3	45432200	45526433
TRAPPC4	51399	11	q23.3	118889241	118894382
TRIM9	114088	14	q22.1	51441980	51562779
TRIP6	7205	7	q22.1	100464760	100471076
TRO	7216	X	p11.21	54946895	54957864
TROAP	10024	12	q13.12	49716971	49725514
TRPC6	7225	11	q22.1	101322295	101454659
TRPM3	80036	9	q21.13	73149949	74061820
TTYH1	57348	19	q13.42	54926373	54948080
TUBB2A	7280	6	p25.2	3153903	3157760
TUBB2B	347733	6	p25.2	3224517	3231964
TUBB3	4157	16	q24.3	89985667	90002505
TULP1	7287	6	p21.31	35465651	35480715
TYRO3	7301	15	q15.1	41851232	41871536
UCN	7349	2	p23.3	27530268	27531313
UGT8	7368	4	q26	115519611	115599380
UHMK1	127933	1	q23.3	162467633	162499400
ULK1	8408	12	q24.33	132379279	132407696

UNC13A	23025	19	p13.11	17712137	17799401
UNC13B	10497	9	p13.3	35161999	35405335
UNC13C	440279	15	q21.3	54305101	54920806
USH1C	10083	11	p15.1	17515442	17565963
USH2A	7399	1	q41	215796236	216596738
UTRN	7402	6	q24.2	144606837	145174170
VAMP1	6843	12	p13.31	6571406	6580065
VAMP2	6844	17	p13.1	8054134	8066864
VAMP3	9341	1	p36.23	7831329	7841492
VAX1	11023	10	q25.3	118888032	118897812
VCAM1	7412	1	p21.2	101185305	101204601
VCAN	1462	5	q14.2	82767284	82878122
VCL	7414	10	q22.2	75757872	75879918
VWF	7450	12	p13.31	6058040	6233836
WISP1	8840	8	q24.22	134203282	134241569
WISP2	8839	20	q13.12	43343485	43357150
WNT10B	7480	12	q13.12	49359123	49365546
WNT11	7481	11	q13.5	75897370	75917574
WNT2	7472	7	q31.2	116916685	116963343
WNT2B	7482	1	p13.2	113009163	113065288
WNT3	7473	17	q21.31	44839876	44895981
WNT3A	89780	1	q42.13	228194752	228248961
WNT5A	7474	3	p14.3	55499743	55523973
WNT5B	81029	12	p13.33	1726222	1756409
WNT6	7475	2	q35	219724544	219738955
WNT7B	7477	22	q13.31	46316242	46373009
WNT8B	7479	10	q24.31	102222798	102243501
WNT9A	7483	1	q42.13	228106357	228135631
WNT9B	7484	17	q21.32	44910712	44962485
WWP1	11059	8	q21.3	87354967	87490649
XRCC4	7518	5	q14.2	82373317	82649606
ZBTB16	7704	11	q23.2	113930315	114121395
ZEB1	6935	10	p11.22	31608126	31818742
ZEB2	9839	2	q22.3	145145568	145282147
ZIC1	7545	3	q24	147111209	147228080
ZIC2	7546	13	q32.3	100634026	100639018
ZNRF1	84937	16	q23.1	75033250	75144611
ZNRF2	223082	7	p14.3	30323923	30407308
ZYX	7791	7	q34	143078173	143088204
