# Supplementary material for: A Brain Region-Specific Predictive Gene Map for Autism Derived by Profiling a Reference Gene Set
Source: PLoS One. 2011 Dec 9;6(12):e28431. doi: 10.1371/journal.pone.0028431 (PMC3235126; doi:10.1371/journal.pone.0028431)
Supplement: Table S8 — Significantly enriched cytoband categories for the predictive set of 1185 genes using DAVID analysis. (PDF) [file pone.0028431.s010.pdf]

Supplementary Table S8. Significantly enriched cytoband categories for the predictive set of 1185 ASD candidate genes using DAVID analysis.

Term	Genes in list with term	Category size	Fold Enrichment	p-value*
5q31	44	114	13.87	4.21E-37
4p12	7	19	13.24	9.07E-06
3p21	6	31	6.95	0.00153
10q24	6	33	6.53	0.00205
15q24	5	21	8.56	0.00244
19q13.1	7	55	4.57	0.00414
11q23	6	40	5.39	0.00484
3q29	8	86	3.34	0.01013
19q13.3	8	96	2.99	0.01775
11q23.3	7	75	3.35	0.01809
17q21	6	60	3.59	0.02556
16q22.1	8	109	2.64	0.03273
17p13.1	8	110	2.61	0.03415

* Uncorrected p-value
