# Supplementary material for: A Brain Region-Specific Predictive Gene Map for Autism Derived by Profiling a Reference Gene Set
Source: PLoS One. 2011 Dec 9;6(12):e28431. doi: 10.1371/journal.pone.0028431 (PMC3235126; doi:10.1371/journal.pone.0028431)
Supplement: Table S9 — Set of 460 predicted ASD candidate genes matching the AutRef84 dual profile. (PDF) [file pone.0028431.s011.pdf]

Supplementary Table S9. Set of 460 predicted ASD candidate genes matching the dual AutRef84 profile.

Gene	Chromosome	Band	Start Point	End Point	# of Enriched Regions	Enriched annotation terms
ACAN	15	15q26.1	89346674	89418585	3	Prefrontal Cortex_3rd,Pituitary_3rd,Occipital Lobe_3rd
ACCN1	17	17q12	31340105	32483825	3	Olfactory Bulb_3rd,Prefrontal Cortex_3rd,Pituitary_3rd
ACCN2	12	12q12	50451487	50477394	4	Olfactory Bulb_3rd,Prefrontal Cortex_3rd,Pituitary_3rd,Occipital Lobe_3rd
ACCN3	7	7q35	150745605	150749843	1	Prefrontal Cortex_3rd
ACCN4	2	2q35	220378892	220403494	2	Prefrontal Cortex_3rd,Pituitary_3rd
ACTN2	1	1q42-q43	236849770	236927558	4	Olfactory Bulb_3rd,Prefrontal Cortex_3rd,Pituitary_3rd,Occipital Lobe_3rd
ACVR1B	12	12q13	52345451	52390863	4	Olfactory Bulb_3rd,Prefrontal Cortex_3rd,Pituitary_3rd,Occipital Lobe_3rd
ADAM22	7	7q21	87563702	87826454	1	Prefrontal Cortex_3rd
ADAM23	2	2q33	207308368	207482685	4	Olfactory Bulb_3rd,Prefrontal Cortex_3rd,Pituitary_3rd,Occipital Lobe_3rd
ADD1	4	4p16.3	2845584	2931789	3	Olfactory Bulb_3rd,Pituitary_3rd,Occipital Lobe_3rd
AGGF1	5	5q13.3	76326232	76361038	1	Olfactory Bulb_3rd
AGRN	1	1p36.33	955503	991492	2	Olfactory Bulb_3rd,Pituitary_3rd
ALCAM	3	3q13.1	105085713	105295744	3	Olfactory Bulb_3rd,Pituitary_3rd,Occipital Lobe_3rd
ALDH1A2	15	15q21.3	58245627	58357906	4	Olfactory Bulb_3rd,Prefrontal Cortex_3rd,Pituitary_3rd,Occipital Lobe_3rd
AMPH	7	7p14-p13	38423305	38671020	4	Olfactory Bulb_3rd,Prefrontal Cortex_3rd,Pituitary_3rd,Occipital Lobe_3rd
ANK3	10	10q21	61788159	62149488	4	Olfactory Bulb_3rd,Prefrontal Cortex_3rd,Pituitary_3rd,Occipital Lobe_3rd
APBA1	9	9q13-q21.1	72050667	72099278	1	Occipital Lobe_3rd
APBB1	11	11p15	6416355	6440644	4	Olfactory Bulb_3rd,Prefrontal Cortex_3rd,Pituitary_3rd,Occipital Lobe_3rd
APBB2	4	4p13	40812044	41216635	4	Olfactory Bulb_3rd,Prefrontal Cortex_3rd,Pituitary_3rd,Occipital Lobe_3rd
APLP1	19	19q13.1	36359401	36370699	4	Olfactory Bulb_3rd,Prefrontal Cortex_3rd,Pituitary_3rd,Occipital Lobe_3rd
APP	21	21q21.2|21q21.3	27252861	27543138	4	Olfactory Bulb_3rd,Prefrontal Cortex_3rd,Pituitary_3rd,Occipital Lobe_3rd
ARC	8	8q24.3	143692410	143695833	3	Olfactory Bulb_3rd,Pituitary_3rd,Occipital Lobe_3rd
ARHGAP32	11	11q24.3	128834955	129062093	4	Olfactory Bulb_3rd,Prefrontal Cortex_3rd,Pituitary_3rd,Occipital Lobe_3rd
ARHGAP5	14	14q12	32546495	32628934	3	Olfactory Bulb_3rd,Prefrontal Cortex_3rd,Occipital Lobe_3rd
ARNT2	15	15q24	80696692	80890278	4	Olfactory Bulb_3rd,Prefrontal Cortex_3rd,Pituitary_3rd,Occipital Lobe_3rd
ARVCF	22	22q11.21	19957402	20004309	2	Prefrontal Cortex_3rd,Pituitary_3rd
ASCL1	12	12q23.2	103351452	103354294	3	Olfactory Bulb_3rd,Pituitary_3rd,Occipital Lobe_3rd

ATN1	12	12p13.31	7033626	7051484	1	Prefrontal Cortex_3rd
ATOH1	4	4q22	94750078	94751142	1	Prefrontal Cortex_3rd
ATP13A2	1	1p36	17312453	17338423	3	Olfactory Bulb_3rd,Pituitary_3rd,Occipital Lobe_3rd
ATP1A2	1	1q21-q23	160085520	160113381	4	Olfactory Bulb_3rd,Prefrontal Cortex_3rd,Pituitary_3rd,Occipital Lobe_3rd
ATP1A3	19	19q13.31	42470734	42498382	4	Olfactory Bulb_3rd,Prefrontal Cortex_3rd,Pituitary_3rd,Occipital Lobe_3rd
ATP1B1	1	1q24	169075947	169101960	4	Olfactory Bulb_3rd,Prefrontal Cortex_3rd,Pituitary_3rd,Occipital Lobe_3rd
ATP1B2	17	17p13.1	7554254	7561089	4	Olfactory Bulb_3rd,Prefrontal Cortex_3rd,Pituitary_3rd,Occipital Lobe_3rd
ATP2A2	12	12q24.11	110719032	110788898	4	Olfactory Bulb_3rd,Prefrontal Cortex_3rd,Pituitary_3rd,Occipital Lobe_3rd
ATP2A3	17	17p13.3	3827169	3867736	2	Prefrontal Cortex_3rd,Pituitary_3rd
ATP2B1	12	12q21.3	89981826	90049844	4	Olfactory Bulb_3rd,Prefrontal Cortex_3rd,Pituitary_3rd,Occipital Lobe_3rd
ATP2B2	3	3p25.3	10365707	10547268	4	Olfactory Bulb_3rd,Prefrontal Cortex_3rd,Pituitary_3rd,Occipital Lobe_3rd
ATP2B3	X	Xq28	152801580	152848387	4	Olfactory Bulb_3rd,Prefrontal Cortex_3rd,Pituitary_3rd,Occipital Lobe_3rd
ATP2B4	1	1q32.1	203595928	203713209	1	Olfactory Bulb_3rd
ATP2C1	3	3q22.1	130613434	130735556	3	Olfactory Bulb_3rd,Pituitary_3rd,Occipital Lobe_3rd
ATP4B	13	13q34	114303119	114312513	1	Pituitary_3rd
ATXN10	22	22q13.31	46067678	46241187	1	Olfactory Bulb_3rd
BAI1	8	8q24	143545377	143626368	2	Prefrontal Cortex_3rd,Pituitary_3rd
BCAM	19	19q13.2	45312338	45324678	1	Pituitary_3rd
BCAN	1	1q31	156611740	156629320	4	Olfactory Bulb_3rd,Prefrontal Cortex_3rd,Pituitary_3rd,Occipital Lobe_3rd
BGLAP	1	1q25-q31	156211951	156213112	1	Prefrontal Cortex_3rd
BSN	3	3p21.31	49591922	49708982	4	Olfactory Bulb_3rd,Prefrontal Cortex_3rd,Pituitary_3rd,Occipital Lobe_3rd
BTD	3	3p25	15643255	15687325	1	Occipital Lobe_3rd
CABP1	12	12q24.31	121078422	121105127	4	Olfactory Bulb_3rd,Prefrontal Cortex_3rd,Pituitary_3rd,Occipital Lobe_3rd
CACNB4	2	2q22-q23	152689285	152955593	4	Olfactory Bulb_3rd,Prefrontal Cortex_3rd,Pituitary_3rd,Occipital Lobe_3rd
CADM3	1	1q21.2-q22	159141377	159172932	4	Olfactory Bulb_3rd,Prefrontal Cortex_3rd,Pituitary_3rd,Occipital Lobe_3rd
CADPS	3	3p14.2	62384021	62861064	2	Prefrontal Cortex_3rd,Occipital Lobe_3rd
CADPS2	7	7q31.3	121958478	122526813	3	Olfactory Bulb_3rd,Pituitary_3rd,Occipital Lobe_3rd
CAMK2A	5	5q32	149599054	149669403	4	Olfactory Bulb_3rd,Prefrontal Cortex_3rd,Pituitary_3rd,Occipital Lobe_3rd
CAMK2N1	1	1p36.12	20808884	20812728	4	Olfactory Bulb_3rd,Prefrontal Cortex_3rd,Pituitary_3rd,Occipital Lobe_3rd
CASK	X	Xp11.4	41374187	41782287	1	Olfactory Bulb_3rd

CCDC64	12	12q24.23	120427648	120532299	1	Prefrontal Cortex_3rd
CCL11	17	17q21.1-q21.2	32612687	32615199	3	Prefrontal Cortex_3rd,Pituitary_3rd,Occipital Lobe_3rd
CCL2	17	17q11.2-q12	32582296	32584222	1	Prefrontal Cortex_3rd
CCR1	3	3p21	46243200	46249832	1	Prefrontal Cortex_3rd
CD2	1	1p13.1	117297086	117311851	1	Occipital Lobe_3rd
CD209	19	19p13	7804879	7812464	1	Occipital Lobe_3rd
CD22	19	19q13.1	35820072	35838264	1	Pituitary_3rd
CD226	18	18q22.3	67530192	67624232	1	Occipital Lobe_3rd
CD33	19	19q13.3	51728335	51743274	1	Occipital Lobe_3rd
CD47	3	3q13.1-q13.2	107761941	107809935	3	Prefrontal Cortex_3rd,Pituitary_3rd,Occipital Lobe_3rd
CD6	11	11q13	60739115	60787849	1	Pituitary_3rd
CD84	1	1q24	160510884	160549306	2	Prefrontal Cortex_3rd,Occipital Lobe_3rd
CDH11	16	16q22.1	64980683	65155919	1	Olfactory Bulb_3rd
CDH12	5	5p14.3	21750973	22853731	2	Olfactory Bulb_3rd,Pituitary_3rd
CDH15	16	16q24.3	89238163	89261900	1	Prefrontal Cortex_3rd
CDH17	8	8q22.1	95139394	95229531	2	Prefrontal Cortex_3rd,Occipital Lobe_3rd
CDH18	5	5p14.3	19473140	19988353	4	Olfactory Bulb_3rd,Prefrontal Cortex_3rd,Pituitary_3rd,Occipital Lobe_3rd
CDH2	18	18q11.2	25530930	25757445	2	Olfactory Bulb_3rd,Pituitary_3rd
CDH22	20	20q13.1	44802376	44880334	1	Prefrontal Cortex_3rd
CDH4	20	20q13.3	59827559	60512299	1	Pituitary_3rd
CDH6	5	5p13.3	31193796	31325237	3	Olfactory Bulb_3rd,Prefrontal Cortex_3rd,Occipital Lobe_3rd
CDH8	16	16q22.1	61687235	62070036	1	Pituitary_3rd
CDHR5	11	11p15.5	616565	625067	1	Pituitary_3rd
CDK5	7	7q36	150750899	150755052	4	Olfactory Bulb_3rd,Prefrontal Cortex_3rd,Pituitary_3rd,Occipital Lobe_3rd
CDK5R1	17	17q11.2	30814105	30818271	4	Olfactory Bulb_3rd,Prefrontal Cortex_3rd,Pituitary_3rd,Occipital Lobe_3rd
CELSR2	1	1p21	109792641	109818378	4	Olfactory Bulb_3rd,Prefrontal Cortex_3rd,Pituitary_3rd,Occipital Lobe_3rd
CELSR3	3	3p21.31	48673896	48700348	3	Olfactory Bulb_3rd,Pituitary_3rd,Occipital Lobe_3rd
CHL1	3	3p26.1	238650	451098	4	Olfactory Bulb_3rd,Prefrontal Cortex_3rd,Pituitary_3rd,Occipital Lobe_3rd
CHRD	3	3q27	184097861	184107617	1	Pituitary_3rd
CHRDL1	X	Xq23	109917084	110039286	1	Olfactory Bulb_3rd

CHRM5	15	15q26	34261089	34357287	2	Prefrontal Cortex_3rd,Pituitary_3rd
CHRNA2	8	8p21	27317278	27336813	1	Occipital Lobe_3rd
CHRNA3	15	15q24	78885394	78913637	2	Prefrontal Cortex_3rd,Occipital Lobe_3rd
CHRNB3	8	8p11.2	42552562	42592209	1	Occipital Lobe_3rd
CHRND	2	2q33-q34	233390922	233400205	1	Pituitary_3rd
CHST10	2	2q11.2	101008322	101034091	2	Pituitary_3rd,Occipital Lobe_3rd
CHST4	16	16q22.2	71560023	71572493	2	Pituitary_3rd,Occipital Lobe_3rd
CHST8	19	19q13.1	34112861	34264414	2	Olfactory Bulb_3rd,Pituitary_3rd
CLCA2	1	1p22.3	86889769	86922241	1	Pituitary_3rd
CLDN1	3	3q28-q29	190023490	190040235	1	Occipital Lobe_3rd
CLDN10	13	13q31-q34	96085853	96232010	3	Olfactory Bulb_3rd,Pituitary_3rd,Occipital Lobe_3rd
CLDN11	3	3q26.2-q26.3	170136653	170152479	1	Occipital Lobe_3rd
CLN5	13	13q21.1-q32	77566059	77576652	1	Pituitary_3rd
CLSTN1	1	1p36.22	9789079	9884550	4	Olfactory Bulb_3rd,Prefrontal Cortex_3rd,Pituitary_3rd,Occipital Lobe_3rd
CLSTN3	12	12p13.31	7282967	7311541	4	Olfactory Bulb_3rd,Prefrontal Cortex_3rd,Pituitary_3rd,Occipital Lobe_3rd
CNTN1	12	12q11-q12	41086358	41464094	4	Olfactory Bulb_3rd,Prefrontal Cortex_3rd,Pituitary_3rd,Occipital Lobe_3rd
CNTN2	1	1q32.1	205012340	205047138	3	Olfactory Bulb_3rd,Pituitary_3rd,Occipital Lobe_3rd
CNTN6	3	3p26-p25	1134629	1445278	1	Prefrontal Cortex_3rd
CNTNAP1	17	17q21	40834632	40852011	4	Olfactory Bulb_3rd,Prefrontal Cortex_3rd,Pituitary_3rd,Occipital Lobe_3rd
COL14A1	8	8q23	121137352	121384273	2	Pituitary_3rd,Occipital Lobe_3rd
COL16A1	1	1p35-p34	32117848	32169768	1	Pituitary_3rd
COL4A3	2	2q36-q37	228029281	228179508	1	Occipital Lobe_3rd
COL5A1	9	9q34.2-q34.3	137533652	137736689	3	Olfactory Bulb_3rd,Prefrontal Cortex_3rd,Pituitary_3rd
COL5A3	19	19p13.2	10070237	10121147	3	Olfactory Bulb_3rd,Prefrontal Cortex_3rd,Occipital Lobe_3rd
COL6A1	21	21q22.3	47401663	47424963	2	Prefrontal Cortex_3rd,Pituitary_3rd
COL8A1	3	3q12.3	99357454	99515158	1	Pituitary_3rd
COPS2	15	15q21.2	49417471	49447854	1	Olfactory Bulb_3rd
CPEB1	15	15q25.2	83211951	83316728	3	Olfactory Bulb_3rd,Pituitary_3rd,Occipital Lobe_3rd
CPLX3	15	15q24.1	75118951	75124136	2	Prefrontal Cortex_3rd,Pituitary_3rd
CRIPT	2	2p21	46844325	46852881	1	Occipital Lobe_3rd

CTNNA2	2	2p12-p11.1	79740060	80875993	4	Olfactory Bulb_3rd,Prefrontal Cortex_3rd,Pituitary_3rd,Occipital Lobe_3rd
CTNND2	5	5p15.2	10971952	11904110	4	Olfactory Bulb_3rd,Prefrontal Cortex_3rd,Pituitary_3rd,Occipital Lobe_3rd
CX3CL1	16	16q13	57406414	57418956	4	Olfactory Bulb_3rd,Prefrontal Cortex_3rd,Pituitary_3rd,Occipital Lobe_3rd
CX3CR1	3	3p21|3p21.3	39304985	39323226	1	Olfactory Bulb_3rd
CXADR	21	21q21.1	18885330	18939276	1	Olfactory Bulb_3rd
CXCR3	X	Xq13	70835766	70838367	1	Prefrontal Cortex_3rd
CYP26A1	10	10q23-q24	94833232	94837641	1	Occipital Lobe_3rd
DAPK3	19	19p13.3	3958452	3969826	1	Occipital Lobe_3rd
DCBLD2	3	3q12.1|3	98514814	98620533	2	Prefrontal Cortex_3rd,Occipital Lobe_3rd
DCHS1	11	11p15.4	6642558	6677080	1	Pituitary_3rd
DCX	X	Xq22.3-q23	110537007	110655406	2	Pituitary_3rd,Occipital Lobe_3rd
DES	2	2q35	220283099	220291461	1	Pituitary_3rd
DLG1	3	3q29	196769431	197025447	3	Olfactory Bulb_3rd,Pituitary_3rd,Occipital Lobe_3rd
DLG2	11	11q14.1	83166055	85338314	1	Pituitary_3rd
DLG4	17	17p13.1	7093209	7123369	2	Prefrontal Cortex_3rd,Pituitary_3rd
DLGAP1	18	18p11.3	3498837	3880135	1	Occipital Lobe_3rd
DLGAP4	20	20q11.23	34995444	35157040	1	Occipital Lobe_3rd
DMXL2	15	15q21.2	51739921	51914967	2	Olfactory Bulb_3rd,Occipital Lobe_3rd
DRP2	X	Xq22	100474933	100519485	1	Occipital Lobe_3rd
DSC1	18	18q12.2|18q12.1	28709199	28742819	1	Occipital Lobe_3rd
DSCAM	21	21q22.2	41384343	42219039	2	Prefrontal Cortex_3rd,Pituitary_3rd
DST	6	6p12.1	56322785	56507694	4	Olfactory Bulb_3rd,Prefrontal Cortex_3rd,Pituitary_3rd,Occipital Lobe_3rd
DTNA	18	18q12	32073254	32471808	4	Olfactory Bulb_3rd,Prefrontal Cortex_3rd,Pituitary_3rd,Occipital Lobe_3rd
DTNB	2	2p24	25600112	25896503	2	Prefrontal Cortex_3rd,Pituitary_3rd
EDIL3	5	5q14	83238126	83680611	4	Olfactory Bulb_3rd,Prefrontal Cortex_3rd,Pituitary_3rd,Occipital Lobe_3rd
EDN3	20	20q13.2-q13.3	57875499	57901047	1	Pituitary_3rd
EFHD1	2	2q37.1	233470767	233547491	4	Olfactory Bulb_3rd,Prefrontal Cortex_3rd,Pituitary_3rd,Occipital Lobe_3rd
EFS	14	14q11.2-q12	23825611	23834842	3	Olfactory Bulb_3rd,Pituitary_3rd,Occipital Lobe_3rd
EMX2	10	10q26.1	119301956	119309057	1	Occipital Lobe_3rd
ENAH	1	1q42.12	225674534	225840845	2	Olfactory Bulb_3rd,Occipital Lobe_3rd

ERBB3	12	12q13	56473892	56497128	3	Olfactory Bulb_3rd,Pituitary_3rd,Occipital Lobe_3rd
ERC2	3	3p14.3	55542336	56502391	4	Olfactory Bulb_3rd,Prefrontal Cortex_3rd,Pituitary_3rd,Occipital Lobe_3rd
F11R	1	1q21.2-q21.3	160965001	160991133	1	Pituitary_3rd
F8	X	Xq28	154064063	154250998	2	Pituitary_3rd,Occipital Lobe_3rd
FAIM2	12	12q13	50260680	50297720	4	Olfactory Bulb_3rd,Prefrontal Cortex_3rd,Pituitary_3rd,Occipital Lobe_3rd
FAT2	5	5q33.1	150883653	150948505	1	Pituitary_3rd
FERMT2	14	14q22.1	53323989	53417815	1	Olfactory Bulb_3rd
FEZ1	11	11q24.2	125315648	125366123	4	Olfactory Bulb_3rd,Prefrontal Cortex_3rd,Pituitary_3rd,Occipital Lobe_3rd
FGFR1	8	8p12	38268656	38326352	1	Pituitary_3rd
FLRT1	11	11q12-q13	63871362	63886645	2	Pituitary_3rd,Occipital Lobe_3rd
FLRT2	14	14q24-q32	85996488	86094270	3	Olfactory Bulb_3rd,Pituitary_3rd,Occipital Lobe_3rd
FLRT3	20	20p11	14304639	14318313	2	Olfactory Bulb_3rd,Pituitary_3rd
FN1	2	2q34	216225177	216300791	1	Occipital Lobe_3rd
FXYD2	11	11q23	117690790	117698807	2	Prefrontal Cortex_3rd,Pituitary_3rd
FZD3	8	8p21	28351773	28421982	2	Olfactory Bulb_3rd,Prefrontal Cortex_3rd
GABBR2	9	9q22.1-q22.3	101050364	101471479	4	Olfactory Bulb_3rd,Prefrontal Cortex_3rd,Pituitary_3rd,Occipital Lobe_3rd
GABRA1	5	5q34-q35	161274197	161326965	4	Olfactory Bulb_3rd,Prefrontal Cortex_3rd,Pituitary_3rd,Occipital Lobe_3rd
GABRA2	4	4p12	46251575	46392056	4	Olfactory Bulb_3rd,Prefrontal Cortex_3rd,Pituitary_3rd,Occipital Lobe_3rd
GABRA5	15	15q11.2-q12	27111866	27194357	4	Olfactory Bulb_3rd,Prefrontal Cortex_3rd,Pituitary_3rd,Occipital Lobe_3rd
GABRB1	4	4p12	47033295	47428462	4	Olfactory Bulb_3rd,Prefrontal Cortex_3rd,Pituitary_3rd,Occipital Lobe_3rd
GABRB3	15	15q11.2-q12	26788693	27018935	2	Olfactory Bulb_3rd,Prefrontal Cortex_3rd
GABRD	1	1p|1p36.3	1950768	1962192	4	Olfactory Bulb_3rd,Prefrontal Cortex_3rd,Pituitary_3rd,Occipital Lobe_3rd
GABRG2	5	5q34	161494648	161582545	4	Olfactory Bulb_3rd,Prefrontal Cortex_3rd,Pituitary_3rd,Occipital Lobe_3rd
GABRP	5	5q33-q34	170210723	170241051	1	Prefrontal Cortex_3rd
GABRR2	6	6q14-q21|6q13-q16.3	89967239	90024967	1	Pituitary_3rd
GAD1	2	2q31	171673200	171717661	4	Olfactory Bulb_3rd,Prefrontal Cortex_3rd,Pituitary_3rd,Occipital Lobe_3rd
GAD2	10	10p11.23	26505236	26593491	4	Olfactory Bulb_3rd,Prefrontal Cortex_3rd,Pituitary_3rd,Occipital Lobe_3rd
GAP43	3	3q13.1-q13.2	115342151	115440334	4	Olfactory Bulb_3rd,Prefrontal Cortex_3rd,Pituitary_3rd,Occipital Lobe_3rd
GAS7	17	17p13.1	9813926	10101868	4	Olfactory Bulb_3rd,Prefrontal Cortex_3rd,Pituitary_3rd,Occipital Lobe_3rd
GLI3	7	7p13	42000547	42276618	1	Prefrontal Cortex_3rd

GLRA3	4	4q33-q34	175563198	175750465	1	Prefrontal Cortex_3rd
GLRB	4	4q31.3	157997277	158093242	4	Olfactory Bulb_3rd,Prefrontal Cortex_3rd,Pituitary_3rd,Occipital Lobe_3rd
GNAO1	16	16q13	56225251	56391356	4	Olfactory Bulb_3rd,Prefrontal Cortex_3rd,Pituitary_3rd,Occipital Lobe_3rd
GP1BB	22	22q11.21-q11.23|22q11.21	19711066	19712297	4	Olfactory Bulb_3rd,Prefrontal Cortex_3rd,Pituitary_3rd,Occipital Lobe_3rd
GPHN	14	14q23.3	66974125	67648525	3	Olfactory Bulb_3rd,Pituitary_3rd,Occipital Lobe_3rd
GRIA1	5	5q33|5q31.1	152870084	153193429	4	Olfactory Bulb_3rd,Prefrontal Cortex_3rd,Pituitary_3rd,Occipital Lobe_3rd
GRIA2	4	4q32-q33	158141736	158287227	4	Olfactory Bulb_3rd,Prefrontal Cortex_3rd,Pituitary_3rd,Occipital Lobe_3rd
GRIK1	21	21q22.11	30909254	31312282	1	Prefrontal Cortex_3rd
GRIK2	6	6q16.3-q21	101846861	102517958	4	Olfactory Bulb_3rd,Prefrontal Cortex_3rd,Pituitary_3rd,Occipital Lobe_3rd
GRIK5	19	19q13.2	42502473	42569957	3	Olfactory Bulb_3rd,Prefrontal Cortex_3rd,Pituitary_3rd
GRIN1	9	9q34.3	140033609	140063214	4	Olfactory Bulb_3rd,Prefrontal Cortex_3rd,Pituitary_3rd,Occipital Lobe_3rd
GRIN2A	16	16p13.2	9847265	10276611	4	Olfactory Bulb_3rd,Prefrontal Cortex_3rd,Pituitary_3rd,Occipital Lobe_3rd
HAPLN2	1	1q23.1	156589086	156595517	4	Olfactory Bulb_3rd,Prefrontal Cortex_3rd,Pituitary_3rd,Occipital Lobe_3rd
HCN2	19	19p13.3	589893	617159	4	Olfactory Bulb_3rd,Prefrontal Cortex_3rd,Pituitary_3rd,Occipital Lobe_3rd
HES1	3	3q28-q29	193853934	193856396	1	Prefrontal Cortex_3rd
HOMER1	5	5q14.2	78669786	78809700	4	Olfactory Bulb_3rd,Prefrontal Cortex_3rd,Pituitary_3rd,Occipital Lobe_3rd
HOMER3	19	19p13.11	19040010	19052041	1	Prefrontal Cortex_3rd
HPCAL4	1	1p34.2	40144645	40157089	4	Olfactory Bulb_3rd,Prefrontal Cortex_3rd,Pituitary_3rd,Occipital Lobe_3rd
HTT	4	4p16.3	3076408	3245687	3	Olfactory Bulb_3rd,Pituitary_3rd,Occipital Lobe_3rd
ICA1	7	7p22	8152814	8302185	2	Pituitary_3rd,Occipital Lobe_3rd
ICAM1	19	19p13.3-p13.2	10381517	10397291	2	Prefrontal Cortex_3rd,Pituitary_3rd
IGF1R	15	15q26.3	99192761	99507759	1	Prefrontal Cortex_3rd
IGFALS	16	16p13.3	1840414	1844909	2	Prefrontal Cortex_3rd,Pituitary_3rd
IGFBP7	4	4q12	57897244	57976539	1	Occipital Lobe_3rd
IL1RAPL2	X	Xq22	103810996	105011822	1	Occipital Lobe_3rd
IL6	7	7p21	22766766	22771621	1	Prefrontal Cortex_3rd
INSR	19	19p13.3-p13.2	7112266	7294011	1	Pituitary_3rd
ITGA2	5	5q11.2	52285156	52390609	1	Pituitary_3rd
ITGA9	3	3p21.3	37493813	37861281	1	Occipital Lobe_3rd

ITGAM	16	16p11.2	31271288	31344213	1	Occipital Lobe_3rd
ITGAV	2	2q31-q32	187454790	187545628	2	Olfactory Bulb_3rd,Occipital Lobe_3rd
ITGB3	17	17q21.32	45331208	45390077	1	Prefrontal Cortex_3rd
ITGBL1	13	13q33	102104966	102368794	1	Occipital Lobe_3rd
ITM2C	2	2q37	231729621	231743963	4	Olfactory Bulb_3rd,Prefrontal Cortex_3rd,Pituitary_3rd,Occipital Lobe_3rd
ITSN1	21	21q22.1-q22.2	35014784	35261609	1	Prefrontal Cortex_3rd
KAL1	X	Xp22.32	8496915	8700227	1	Olfactory Bulb_3rd
KLK6	19	19q13.3	51461887	51472929	4	Olfactory Bulb_3rd,Prefrontal Cortex_3rd,Pituitary_3rd,Occipital Lobe_3rd
L1CAM	X	Xq28	153126971	153141399	4	Olfactory Bulb_3rd,Prefrontal Cortex_3rd,Pituitary_3rd,Occipital Lobe_3rd
LAMA2	6	6q22-q23	129204286	129837711	1	Pituitary_3rd
LAMA4	6	6q21	112429134	112575828	3	Prefrontal Cortex_3rd,Pituitary_3rd,Occipital Lobe_3rd
LEF1	4	4q23-q25	108968701	109090112	1	Prefrontal Cortex_3rd
LGALS4	19	19q13.2	39292311	39303740	2	Prefrontal Cortex_3rd,Occipital Lobe_3rd
LGI1	10	10q24	95517566	95557916	4	Olfactory Bulb_3rd,Prefrontal Cortex_3rd,Pituitary_3rd,Occipital Lobe_3rd
LIG4	13	13q33-q34	108859790	108870716	2	Olfactory Bulb_3rd,Prefrontal Cortex_3rd
LIN7B	19	19q13.3	49617618	49621717	4	Olfactory Bulb_3rd,Prefrontal Cortex_3rd,Pituitary_3rd,Occipital Lobe_3rd
LRRN2	1	1q32.1	204586301	204654597	2	Olfactory Bulb_3rd,Occipital Lobe_3rd
LZTS1	8	8p22	20103676	20112803	4	Olfactory Bulb_3rd,Prefrontal Cortex_3rd,Pituitary_3rd,Occipital Lobe_3rd
MADCAM1	19	19p13.3	496490	505343	1	Prefrontal Cortex_3rd
MAG	19	19q13.1	35783038	35804707	4	Olfactory Bulb_3rd,Prefrontal Cortex_3rd,Pituitary_3rd,Occipital Lobe_3rd
MAGI2	7	7q21	77646374	79082890	3	Olfactory Bulb_3rd,Pituitary_3rd,Occipital Lobe_3rd
MAL	2	2cen-q13	95691479	95719735	3	Olfactory Bulb_3rd,Pituitary_3rd,Occipital Lobe_3rd
MAP1B	5	5q13	71403118	71505397	4	Olfactory Bulb_3rd,Prefrontal Cortex_3rd,Pituitary_3rd,Occipital Lobe_3rd
MAP1S	19	19p13.11	17830303	17845324	1	Olfactory Bulb_3rd
MAP2K1	15	15q22.1-q22.33	66679211	66783882	1	Olfactory Bulb_3rd
MBP	18	18q23	74690789	74844774	4	Olfactory Bulb_3rd,Prefrontal Cortex_3rd,Pituitary_3rd,Occipital Lobe_3rd
MPZL2	11	11q24	118124131	118135251	1	Pituitary_3rd
MYBPC1	12	12q23.2	101988747	102079658	2	Olfactory Bulb_3rd,Pituitary_3rd
MYO7A	11	11q13.5	76839310	76926286	3	Prefrontal Cortex_3rd,Pituitary_3rd,Occipital Lobe_3rd
MYRIP	3	3p22.1	39851303	40301812	1	Olfactory Bulb_3rd

NAPA	19	19q13.33	47990891	48018497	2	Pituitary_3rd,Occipital Lobe_3rd
NCAM1	11	11q23.1	112831995	113149158	4	Olfactory Bulb_3rd,Prefrontal Cortex_3rd,Pituitary_3rd,Occipital Lobe_3rd
NCAN	19	19p12	19322782	19363061	4	Olfactory Bulb_3rd,Prefrontal Cortex_3rd,Pituitary_3rd,Occipital Lobe_3rd
NCDN	1	1p34.3	36023393	36032380	4	Olfactory Bulb_3rd,Prefrontal Cortex_3rd,Pituitary_3rd,Occipital Lobe_3rd
NCKAP1	2	2q32	183789605	183903229	1	Olfactory Bulb_3rd
NCS1	9	9q34	132934857	132999583	3	Prefrontal Cortex_3rd,Pituitary_3rd,Occipital Lobe_3rd
NDN	15	15q11.2-q12	23930554	23932450	1	Olfactory Bulb_3rd
NEDD4L	18	18q21	55711619	56068772	4	Olfactory Bulb_3rd,Prefrontal Cortex_3rd,Pituitary_3rd,Occipital Lobe_3rd
NEFM	8	8p21	24771274	24776606	4	Olfactory Bulb_3rd,Prefrontal Cortex_3rd,Pituitary_3rd,Occipital Lobe_3rd
NELL1	11	11p15.1	20691117	21597232	1	Olfactory Bulb_3rd
NELL2	12	12q12	44902058	45307711	4	Olfactory Bulb_3rd,Prefrontal Cortex_3rd,Pituitary_3rd,Occipital Lobe_3rd
NEUROG1	5	5q23-q31	134869972	134871639	1	Prefrontal Cortex_3rd
NFASC	1	1q32.1	204797782	204991950	4	Olfactory Bulb_3rd,Prefrontal Cortex_3rd,Pituitary_3rd,Occipital Lobe_3rd
NGFR	17	17q21-q22	47572655	47592382	1	Prefrontal Cortex_3rd
NGRN	15	15q26.1	90808895	90815443	4	Olfactory Bulb_3rd,Prefrontal Cortex_3rd,Pituitary_3rd,Occipital Lobe_3rd
NHLH1	1	1q22	160336861	160342638	3	Olfactory Bulb_3rd,Prefrontal Cortex_3rd,Pituitary_3rd
NINJ2	12	12p13	673462	772755	1	Olfactory Bulb_3rd
NNAT	20	20q11.2-q12	36149607	36152092	4	Olfactory Bulb_3rd,Prefrontal Cortex_3rd,Pituitary_3rd,Occipital Lobe_3rd
NOTCH1	9	9q34.3	139388896	139440238	1	Prefrontal Cortex_3rd
NPAS1	19	19q13.2-q13.3	47524143	47549017	1	Pituitary_3rd
NPAS2	2	2q11.2	101436613	101613287	3	Olfactory Bulb_3rd,Prefrontal Cortex_3rd,Occipital Lobe_3rd
NPHS1	19	19q13.1	36316274	36342895	1	Prefrontal Cortex_3rd
NPTN	15	15q22	73852344	73925753	3	Olfactory Bulb_3rd,Pituitary_3rd,Occipital Lobe_3rd
NPTX1	17	17q25.3	78440633	78450404	4	Olfactory Bulb_3rd,Prefrontal Cortex_3rd,Pituitary_3rd,Occipital Lobe_3rd
NRCAM	7	7q31	107788071	108096841	4	Olfactory Bulb_3rd,Prefrontal Cortex_3rd,Pituitary_3rd,Occipital Lobe_3rd
NRG1	8	8p12	31496820	32622558	2	Olfactory Bulb_3rd,Prefrontal Cortex_3rd
NRP1	10	10p12	33466419	33623833	2	Prefrontal Cortex_3rd,Pituitary_3rd
NRP2	2	2q33.3	206547224	206662857	3	Prefrontal Cortex_3rd,Pituitary_3rd,Occipital Lobe_3rd
NRXN2	11	11q13	64373646	64490660	4	Olfactory Bulb_3rd,Prefrontal Cortex_3rd,Pituitary_3rd,Occipital Lobe_3rd
NRXN3	14	14q31	78870093	80330762	4	Olfactory Bulb_3rd,Prefrontal Cortex_3rd,Pituitary_3rd,Occipital Lobe_3rd

NTM	11	11q25	131240371	132206716	3	Olfactory Bulb_3rd,Pituitary_3rd,Occipital Lobe_3rd
OMD	9	9q22.31	95176527	95186836	1	Prefrontal Cortex_3rd
OMG	17	17q11.2	29621668	29624380	4	Olfactory Bulb_3rd,Prefrontal Cortex_3rd,Pituitary_3rd,Occipital Lobe_3rd
OPCML	11	11q25	132284875	133402403	4	Olfactory Bulb_3rd,Prefrontal Cortex_3rd,Pituitary_3rd,Occipital Lobe_3rd
OTOF	2	2p23.1	26680071	26781566	1	Prefrontal Cortex_3rd
P2RX7	12	12q24	121570622	121624354	2	Olfactory Bulb_3rd,Pituitary_3rd
PAX6	11	11p13	31806340	31839509	3	Olfactory Bulb_3rd,Pituitary_3rd,Occipital Lobe_3rd
PCDH1	5	5q31.3	141232672	141257944	1	Prefrontal Cortex_3rd
PCDH11X	X	Xq21.3	91034260	91878229	3	Olfactory Bulb_3rd,Prefrontal Cortex_3rd,Occipital Lobe_3rd
PCDH11Y	Y	Yp11.2	4868267	5610265	2	Olfactory Bulb_3rd,Pituitary_3rd
PCDH12	5	5q31	141324530	141338627	1	Prefrontal Cortex_3rd
PCDH17	13	13q21.1	58205789	58303065	4	Olfactory Bulb_3rd,Prefrontal Cortex_3rd,Pituitary_3rd,Occipital Lobe_3rd
PCDH7	4	4p15	30722037	31148423	4	Olfactory Bulb_3rd,Prefrontal Cortex_3rd,Pituitary_3rd,Occipital Lobe_3rd
PCDH8	13	13q21.1	53418109	53422774	3	Olfactory Bulb_3rd,Pituitary_3rd,Occipital Lobe_3rd
PCDHA10	5	5q31	140235634	140391929	4	Olfactory Bulb_3rd,Prefrontal Cortex_3rd,Pituitary_3rd,Occipital Lobe_3rd
PCDHA13	5	5q31	140261854	140391929	4	Olfactory Bulb_3rd,Prefrontal Cortex_3rd,Pituitary_3rd,Occipital Lobe_3rd
PCDHAC1	5	5q31	140306302	140391929	4	Olfactory Bulb_3rd,Prefrontal Cortex_3rd,Pituitary_3rd,Occipital Lobe_3rd
PCDHAC2	5	5q31	140345747	140391929	4	Olfactory Bulb_3rd,Prefrontal Cortex_3rd,Pituitary_3rd,Occipital Lobe_3rd
PCDHB8	5	5q31	140557430	140560021	1	Prefrontal Cortex_3rd
PCDHGA10	5	5q31	140792743	140892546	1	Prefrontal Cortex_3rd
PCDHGA12	5	5q31	140810158	140892546	3	Olfactory Bulb_3rd,Pituitary_3rd,Occipital Lobe_3rd
PCDHGC4	5	5q31	140864741	140892546	3	Olfactory Bulb_3rd,Pituitary_3rd,Occipital Lobe_3rd
PCDHGC5	5	5q31	140868808	140892546	3	Olfactory Bulb_3rd,Pituitary_3rd,Occipital Lobe_3rd
PCLO	7	7q11.23-q21.3	82383321	82792197	4	Olfactory Bulb_3rd,Prefrontal Cortex_3rd,Pituitary_3rd,Occipital Lobe_3rd
PCP4	21	21q22.2	41239347	41301322	4	Olfactory Bulb_3rd,Prefrontal Cortex_3rd,Pituitary_3rd,Occipital Lobe_3rd
PCSK1	5	5q15-q21	95726040	95768985	4	Olfactory Bulb_3rd,Prefrontal Cortex_3rd,Pituitary_3rd,Occipital Lobe_3rd
PDLIM5	4	4q22	95373038	95589377	1	Pituitary_3rd
PDXK	21	21q22.3	45138978	45182188	3	Olfactory Bulb_3rd,Prefrontal Cortex_3rd,Occipital Lobe_3rd
PDZD2	5	5p13.3	31799031	32111038	2	Olfactory Bulb_3rd,Occipital Lobe_3rd
PHACTR1	6	6p24.1	12717833	13287528	3	Olfactory Bulb_3rd,Prefrontal Cortex_3rd,Occipital Lobe_3rd

PHGDH	1	1p12	120254419	120286849	1	Olfactory Bulb_3rd
PICK1	22	22q13.1	38453262	38471708	1	Pituitary_3rd
PJA2	5	5q21.3	108670410	108745675	3	Olfactory Bulb_3rd,Pituitary_3rd,Occipital Lobe_3rd
PKP4	2	2q24.1	159313476	159537941	4	Olfactory Bulb_3rd,Prefrontal Cortex_3rd,Pituitary_3rd,Occipital Lobe_3rd
PNMA1	14	14q24.3	74178486	74181128	3	Olfactory Bulb_3rd,Pituitary_3rd,Occipital Lobe_3rd
PODXL2	3	3q21.3	127348039	127391652	2	Olfactory Bulb_3rd,Pituitary_3rd
POU3F2	6	6q16	99282580	99286666	2	Olfactory Bulb_3rd,Pituitary_3rd
POU6F2	7	7p14.1	39017609	39504390	2	Prefrontal Cortex_3rd,Pituitary_3rd
PPFIBP1	12	12p12.1	27677045	27848496	1	Prefrontal Cortex_3rd
PPP1R9A	7	7q21.3	94536949	94925727	2	Olfactory Bulb_3rd,Occipital Lobe_3rd
PRKCQ	10	10p15	6469105	6557111	2	Pituitary_3rd,Occipital Lobe_3rd
PRR7	5	5q35.3	176873796	176883287	1	Occipital Lobe_3rd
PRSS12	4	4q28.1	119201193	119273922	1	Occipital Lobe_3rd
PSD	10	10q24	104162376	104178901	3	Olfactory Bulb_3rd,Prefrontal Cortex_3rd,Occipital Lobe_3rd
PSEN1	14	14q24.3	73603143	73690399	3	Olfactory Bulb_3rd,Pituitary_3rd,Occipital Lobe_3rd
PTPRF	1	1p34	43996547	44089343	1	Prefrontal Cortex_3rd
PTPRR	12	12q15	71031862	71314584	2	Olfactory Bulb_3rd,Pituitary_3rd
PTPRT	20	20q12-q13	40701392	41818557	4	Olfactory Bulb_3rd,Prefrontal Cortex_3rd,Pituitary_3rd,Occipital Lobe_3rd
PTPRU	1	1p35.3	29563028	29653325	2	Prefrontal Cortex_3rd,Pituitary_3rd
PTPRZ1	7	7q31.3	121513159	121702090	4	Olfactory Bulb_3rd,Prefrontal Cortex_3rd,Pituitary_3rd,Occipital Lobe_3rd
PTS	11	11q22.3	112097088	112104696	1	Olfactory Bulb_3rd
RAC1	7	7p22	6414126	6443598	2	Olfactory Bulb_3rd,Pituitary_3rd
RARB	3	3p24	25469754	25639423	1	Occipital Lobe_3rd
RASGRP2	11	11q13	64494383	64512928	1	Prefrontal Cortex_3rd
RBPJ	4	4p15.2	26321332	26433278	2	Pituitary_3rd,Occipital Lobe_3rd
RCAN1	21	21q22.1-q22.2|21q22.12	35888782	35987382	4	Olfactory Bulb_3rd,Prefrontal Cortex_3rd,Pituitary_3rd,Occipital Lobe_3rd
RCAN2	6	6p12.3	46188469	46293531	4	Olfactory Bulb_3rd,Prefrontal Cortex_3rd,Pituitary_3rd,Occipital Lobe_3rd
RELN	7	7q22	103112231	103629963	2	Prefrontal Cortex_3rd,Occipital Lobe_3rd
RGS11	16	16p13.3	318310	325914	3	Olfactory Bulb_3rd,Pituitary_3rd,Occipital Lobe_3rd
RGS12	4	4p16.3	3315874	3441640	3	Olfactory Bulb_3rd,Prefrontal Cortex_3rd,Pituitary_3rd

RGS14	5	5q35.3	176784844	176799599	2	Prefrontal Cortex_3rd,Occipital Lobe_3rd
RGS16	1	1q25-q31	182567758	182573548	1	Pituitary_3rd
RGS17	6	6q25.3	153332026	153452389	1	Olfactory Bulb_3rd
RGS20	8	8q	54764368	54871863	2	Olfactory Bulb_3rd,Occipital Lobe_3rd
RGS4	1	1q23.3	163038396	163046592	4	Olfactory Bulb_3rd,Prefrontal Cortex_3rd,Pituitary_3rd,Occipital Lobe_3rd
RGS5	1	1q23.1	163112089	163172963	4	Olfactory Bulb_3rd,Prefrontal Cortex_3rd,Pituitary_3rd,Occipital Lobe_3rd
RGS6	14	14q24.3	72399786	73033238	2	Olfactory Bulb_3rd,Prefrontal Cortex_3rd
RGS9	17	17q24	63133456	63223821	1	Occipital Lobe_3rd
RIMBP2	12	12q24.33	130880681	131002410	4	Olfactory Bulb_3rd,Prefrontal Cortex_3rd,Pituitary_3rd,Occipital Lobe_3rd
RIMS1	6	6q12-q13	72596650	73112845	2	Olfactory Bulb_3rd,Pituitary_3rd
ROBO1	3	3p12	78646388	79817059	2	Olfactory Bulb_3rd,Prefrontal Cortex_3rd
RPH3A	12	12q24.13	113229549	113336686	4	Olfactory Bulb_3rd,Prefrontal Cortex_3rd,Pituitary_3rd,Occipital Lobe_3rd
RPS6KA6	X	Xq21	83313354	83442943	1	Prefrontal Cortex_3rd
RTN1	14	14q23.1	60062694	60337557	4	Olfactory Bulb_3rd,Prefrontal Cortex_3rd,Pituitary_3rd,Occipital Lobe_3rd
RUNX1	21	21q22.3	36160098	36421595	2	Prefrontal Cortex_3rd,Pituitary_3rd
S100B	21	21q22.3	48018531	48025035	4	Olfactory Bulb_3rd,Prefrontal Cortex_3rd,Pituitary_3rd,Occipital Lobe_3rd
SAMD4A	14	14q22.2	55034637	55260033	3	Olfactory Bulb_3rd,Prefrontal Cortex_3rd,Occipital Lobe_3rd
SCAI	9	9q33.3	127704887	127905838	2	Olfactory Bulb_3rd,Pituitary_3rd
SCAMP1	5	5q14.1	77656339	77776562	4	Olfactory Bulb_3rd,Prefrontal Cortex_3rd,Pituitary_3rd,Occipital Lobe_3rd
SCAMP5	15	15q24.2	75287876	75313836	4	Olfactory Bulb_3rd,Prefrontal Cortex_3rd,Pituitary_3rd,Occipital Lobe_3rd
SCARB2	4	4q21.1	77079894	77135035	2	Olfactory Bulb_3rd,Pituitary_3rd
SCN10A	3	3p22.2	38738837	38835501	2	Pituitary_3rd,Occipital Lobe_3rd
SCN1B	19	19q13.1	35521592	35531353	4	Olfactory Bulb_3rd,Prefrontal Cortex_3rd,Pituitary_3rd,Occipital Lobe_3rd
SCN2B	11	11q23	118033519	118047337	4	Olfactory Bulb_3rd,Prefrontal Cortex_3rd,Pituitary_3rd,Occipital Lobe_3rd
SCN3A	2	2q24	165944030	166060577	2	Olfactory Bulb_3rd,Occipital Lobe_3rd
SCN3B	11	11q23.3	123499895	123525315	4	Olfactory Bulb_3rd,Prefrontal Cortex_3rd,Pituitary_3rd,Occipital Lobe_3rd
SCNN1A	12	12p13	6456009	6486523	3	Prefrontal Cortex_3rd,Pituitary_3rd,Occipital Lobe_3rd
SDC3	1	1pter-p22.3	31342313	31381480	2	Olfactory Bulb_3rd,Pituitary_3rd
SDK2	17	17q25.1	71330523	71640227	1	Prefrontal Cortex_3rd
SEMA4F	2	2p13.1	74881393	74909185	4	Olfactory Bulb_3rd,Prefrontal Cortex_3rd,Pituitary_3rd,Occipital Lobe_3rd

SEMA5A	5	5p15.2	9035138	9546233	1	Pituitary_3rd
SEPTIN11	4	4q21.1	77870895	77959768	4	Olfactory Bulb_3rd,Prefrontal Cortex_3rd,Pituitary_3rd,Occipital Lobe_3rd
SERPINI1	3	3q26.1	167453432	167543357	4	Olfactory Bulb_3rd,Prefrontal Cortex_3rd,Pituitary_3rd,Occipital Lobe_3rd
SFXN1	5	-	174905514	174955621	1	Prefrontal Cortex_3rd
SFXN3	10	10q24.31	102790996	102800998	2	Prefrontal Cortex_3rd,Occipital Lobe_3rd
SH3GL2	9	9p22	17578953	17797122	4	Olfactory Bulb_3rd,Prefrontal Cortex_3rd,Pituitary_3rd,Occipital Lobe_3rd
SH3GL3	15	15q24	84116091	84287495	4	Olfactory Bulb_3rd,Prefrontal Cortex_3rd,Pituitary_3rd,Occipital Lobe_3rd
SHC3	9	9q22.1	91620686	91793682	4	Olfactory Bulb_3rd,Prefrontal Cortex_3rd,Pituitary_3rd,Occipital Lobe_3rd
SIGLEC6	19	19q13.3	52022784	52035110	1	Prefrontal Cortex_3rd
SIPA1L1	14	14q24.2	71996042	72206122	4	Olfactory Bulb_3rd,Prefrontal Cortex_3rd,Pituitary_3rd,Occipital Lobe_3rd
SIRPA	20	20p13	1874813	1920540	4	Olfactory Bulb_3rd,Prefrontal Cortex_3rd,Pituitary_3rd,Occipital Lobe_3rd
SLAMF7	1	1q23.1-q24.1	160709077	160724601	2	Pituitary_3rd,Occipital Lobe_3rd
SLC12A2	5	5q23.3	127419483	127525380	1	Pituitary_3rd
SLC12A4	16	16q22.1	67977377	68002597	1	Prefrontal Cortex_3rd
SLC12A5	20	20q13.12	44650329	44688789	4	Olfactory Bulb_3rd,Prefrontal Cortex_3rd,Pituitary_3rd,Occipital Lobe_3rd
SLC12A6	15	15q13	34522197	34630265	1	Pituitary_3rd
SLC13A3	20	20q12-q13.1	45186462	45313124	2	Pituitary_3rd,Occipital Lobe_3rd
SLC17A4	6	6p22-p21.3	25754927	25781403	2	Pituitary_3rd,Occipital Lobe_3rd
SLC17A7	19	19q13	49932655	49944808	4	Olfactory Bulb_3rd,Prefrontal Cortex_3rd,Pituitary_3rd,Occipital Lobe_3rd
SLC1A2	11	11p13-p12	35272752	35441105	4	Olfactory Bulb_3rd,Prefrontal Cortex_3rd,Pituitary_3rd,Occipital Lobe_3rd
SLC1A3	5	5p13	36606457	36688436	4	Olfactory Bulb_3rd,Prefrontal Cortex_3rd,Pituitary_3rd,Occipital Lobe_3rd
SLC23A2	20	20p13	4833002	4982145	1	Olfactory Bulb_3rd
SLC24A2	9	9p22-p13	19507450	19788591	1	Prefrontal Cortex_3rd
SLC24A3	20	20p13	19193290	19703541	2	Olfactory Bulb_3rd,Occipital Lobe_3rd
SLC30A3	2	2p23.3	27477440	27485960	1	Olfactory Bulb_3rd
SLC30A4	15	15q21.1|15q21.1	45774676	45815002	1	Prefrontal Cortex_3rd
SLC30A5	5	5q12.1	68389818	68425880	1	Prefrontal Cortex_3rd
SLC30A9	4	4p13	41992523	42089551	1	Olfactory Bulb_3rd
SLC34A1	5	5q35	176811432	176825849	1	Prefrontal Cortex_3rd
SLC34A2	4	4p15.2	25657435	25680370	2	Prefrontal Cortex_3rd,Occipital Lobe_3rd

SLC38A7	16	16q21	58700298	58718674	2	Prefrontal Cortex_3rd,Pituitary_3rd
SLC41A3	3	3q21.2	125725200	125820391	1	Occipital Lobe_3rd
SLC4A4	4	4q21	72053003	72437804	3	Olfactory Bulb_3rd,Pituitary_3rd,Occipital Lobe_3rd
SLC4A7	3	3p22	27414214	27498245	2	Prefrontal Cortex_3rd,Occipital Lobe_3rd
SLC4A8	12	12q13.13	51818594	51902980	3	Olfactory Bulb_3rd,Prefrontal Cortex_3rd,Pituitary_3rd
SLC5A5	19	19p13.2-p12	17982782	18005983	2	Prefrontal Cortex_3rd,Pituitary_3rd
SLC8A2	19	19q13.3	47931279	47975307	4	Olfactory Bulb_3rd,Prefrontal Cortex_3rd,Pituitary_3rd,Occipital Lobe_3rd
SLC9A1	1	1p36.1-p35	27425300	27481451	1	Occipital Lobe_3rd
SLC9A6	X	Xq26.3	135067583	135129428	3	Olfactory Bulb_3rd,Pituitary_3rd,Occipital Lobe_3rd
SMARCA1	X	Xq25	128580478	128657460	1	Occipital Lobe_3rd
SNAP25	20	20p12-p11.2	10199477	10288065	4	Olfactory Bulb_3rd,Prefrontal Cortex_3rd,Pituitary_3rd,Occipital Lobe_3rd
SNPH	20	20p13	1246960	1289971	4	Olfactory Bulb_3rd,Prefrontal Cortex_3rd,Pituitary_3rd,Occipital Lobe_3rd
SOCS1	16	16p13.13	11348274	11350039	2	Prefrontal Cortex_3rd,Pituitary_3rd
SOCS3	17	17q25.3	76352859	76356158	2	Prefrontal Cortex_3rd,Pituitary_3rd
SPAM1	7	7q31.3	123565286	123611464	1	Prefrontal Cortex_3rd
SPARCL1	4	4q22.1	88394487	88450655	4	Olfactory Bulb_3rd,Prefrontal Cortex_3rd,Pituitary_3rd,Occipital Lobe_3rd
SPOCK1	5	5q31	136310987	136835018	3	Olfactory Bulb_3rd,Pituitary_3rd,Occipital Lobe_3rd
SPON1	11	11p15.2	13983914	14289656	2	Olfactory Bulb_3rd,Prefrontal Cortex_3rd
SPP1	4	4q22.1	88896802	88904563	3	Olfactory Bulb_3rd,Pituitary_3rd,Occipital Lobe_3rd
SSX2IP	1	1p22.3	85109390	85156240	4	Olfactory Bulb_3rd,Prefrontal Cortex_3rd,Pituitary_3rd,Occipital Lobe_3rd
STAB2	12	12q23.3	103981069	104160502	2	Olfactory Bulb_3rd,Prefrontal Cortex_3rd
STMN2	8	8q21.13	80523380	80577237	4	Olfactory Bulb_3rd,Prefrontal Cortex_3rd,Pituitary_3rd,Occipital Lobe_3rd
STX1A	7	7q11.23	73113535	73134017	4	Olfactory Bulb_3rd,Prefrontal Cortex_3rd,Pituitary_3rd,Occipital Lobe_3rd
SUSD5	3	3p22.3	33191537	33260707	2	Prefrontal Cortex_3rd,Occipital Lobe_3rd
SV2B	15	15q26.1	91643182	91844539	4	Olfactory Bulb_3rd,Prefrontal Cortex_3rd,Pituitary_3rd,Occipital Lobe_3rd
SV2C	5	5q13.3	75379305	75621416	4	Olfactory Bulb_3rd,Prefrontal Cortex_3rd,Pituitary_3rd,Occipital Lobe_3rd
SYMPK	19	19q13.3	46318700	46366548	1	Pituitary_3rd
SYN1	X	Xp11.23	47431300	47479256	4	Olfactory Bulb_3rd,Prefrontal Cortex_3rd,Pituitary_3rd,Occipital Lobe_3rd
SYNGR1	22	22q13.1	39745954	39781593	4	Olfactory Bulb_3rd,Prefrontal Cortex_3rd,Pituitary_3rd,Occipital Lobe_3rd
SYNGR3	16	16p13	2039968	2044276	4	Olfactory Bulb_3rd,Prefrontal Cortex_3rd,Pituitary_3rd,Occipital Lobe_3rd

SYP	X	Xp11.23-p11.22	49044265	49056661	4	Olfactory Bulb_3rd,Prefrontal Cortex_3rd,Pituitary_3rd,Occipital Lobe_3rd
SYT1	12	12cen-q21	79257773	79845788	4	Olfactory Bulb_3rd,Prefrontal Cortex_3rd,Pituitary_3rd,Occipital Lobe_3rd
SYT11	1	1q21.2	155829260	155854990	4	Olfactory Bulb_3rd,Prefrontal Cortex_3rd,Pituitary_3rd,Occipital Lobe_3rd
SYT2	1	1q32.1	202559724	202679551	3	Prefrontal Cortex_3rd,Pituitary_3rd,Occipital Lobe_3rd
SYT5	19	19q|11p	55684469	55691720	4	Olfactory Bulb_3rd,Prefrontal Cortex_3rd,Pituitary_3rd,Occipital Lobe_3rd
SYT7	11	11q12-q13.1	61282785	61348298	2	Olfactory Bulb_3rd,Pituitary_3rd
TACC2	10	10q26	123748689	124014057	1	Occipital Lobe_3rd
TAGLN3	3	3q13.2	111717586	111732735	4	Olfactory Bulb_3rd,Prefrontal Cortex_3rd,Pituitary_3rd,Occipital Lobe_3rd
THBS2	6	6q27	169615875	169654137	3	Olfactory Bulb_3rd,Pituitary_3rd,Occipital Lobe_3rd
TLN2	15	15q15-q21	62939510	63136830	4	Olfactory Bulb_3rd,Prefrontal Cortex_3rd,Pituitary_3rd,Occipital Lobe_3rd
TNFAIP6	2	2q23.3	152214105	152236560	1	Prefrontal Cortex_3rd
TNR	1	1q24	175291935	175712752	1	Occipital Lobe_3rd
TP53BP2	1	1q42.1	223967595	224033674	1	Olfactory Bulb_3rd
TPBG	6	6q14-q15	83072923	83077134	2	Prefrontal Cortex_3rd,Occipital Lobe_3rd
TRAPPC10	21	21q22.3	45432206	45526433	1	Occipital Lobe_3rd
TRIM9	14	14q22.1	51441979	51562422	4	Olfactory Bulb_3rd,Prefrontal Cortex_3rd,Pituitary_3rd,Occipital Lobe_3rd
TRO	X	Xp11.22-p11.21	54947249	54957864	4	Olfactory Bulb_3rd,Prefrontal Cortex_3rd,Pituitary_3rd,Occipital Lobe_3rd
TRPM3	9	9q21.12	73149949	73736514	2	Olfactory Bulb_3rd,Pituitary_3rd
TTYH1	19	19q13.4	54926635	54948070	4	Olfactory Bulb_3rd,Prefrontal Cortex_3rd,Pituitary_3rd,Occipital Lobe_3rd
TUBB2A	6	6p25	3153900	3157783	4	Olfactory Bulb_3rd,Prefrontal Cortex_3rd,Pituitary_3rd,Occipital Lobe_3rd
TUBB2B	6	6p25	3224495	3227968	4	Olfactory Bulb_3rd,Prefrontal Cortex_3rd,Pituitary_3rd,Occipital Lobe_3rd
TUBB3	16	16q24.3	89989745	90002505	3	Olfactory Bulb_3rd,Pituitary_3rd,Occipital Lobe_3rd
TULP1	6	6p21.3	35465651	35480647	1	Prefrontal Cortex_3rd
TYRO3	15	15q15	41851220	41871536	4	Olfactory Bulb_3rd,Prefrontal Cortex_3rd,Pituitary_3rd,Occipital Lobe_3rd
UGT8	4	4q26	115519611	115598202	4	Olfactory Bulb_3rd,Prefrontal Cortex_3rd,Pituitary_3rd,Occipital Lobe_3rd
UNC13A	19	19p13.11	17712137	17799008	3	Olfactory Bulb_3rd,Prefrontal Cortex_3rd,Pituitary_3rd
UNC13B	9	9p13.3	35161989	35405332	1	Prefrontal Cortex_3rd
USH1C	11	11p14.3	17515442	17565963	2	Pituitary_3rd,Occipital Lobe_3rd
USH2A	1	1q41	215796236	216596738	1	Occipital Lobe_3rd
UTRN	6	6q24	144612873	145174170	1	Pituitary_3rd

VAMP1	12	12p	6571403	6579843	4	Olfactory Bulb_3rd,Prefrontal Cortex_3rd,Pituitary_3rd,Occipital Lobe_3rd
VAMP2	17	17p13.1	8062465	8066293	4	Olfactory Bulb_3rd,Prefrontal Cortex_3rd,Pituitary_3rd,Occipital Lobe_3rd
VAMP3	1	1p36.23	7831329	7841492	1	Olfactory Bulb_3rd
WISP1	8	8q24.22	134203312	134241571	3	Prefrontal Cortex_3rd,Pituitary_3rd,Occipital Lobe_3rd
WNT10B	12	12q13	49359123	49365641	4	Olfactory Bulb_3rd,Prefrontal Cortex_3rd,Pituitary_3rd,Occipital Lobe_3rd
WNT2	7	7q31.2	116916685	116963343	1	Occipital Lobe_3rd
WNT2B	1	1p13	113033630	113063910	2	Prefrontal Cortex_3rd,Occipital Lobe_3rd
WNT6	2	2q35	219724546	219738954	1	Prefrontal Cortex_3rd
WNT8B	10	10q24	102222812	102243399	1	Pituitary_3rd
XRCC4	5	5q14.2	82373317	82649579	1	Pituitary_3rd
ZBTB16	11	11q23.1	113930431	114121398	2	Pituitary_3rd,Occipital Lobe_3rd
ZEB1	10	10p11.2	31608101	31818742	2	Olfactory Bulb_3rd,Occipital Lobe_3rd
ZEB2	2	2q22.3	145141942	145277958	2	Olfactory Bulb_3rd,Occipital Lobe_3rd
ZIC1	3	3q24	147127181	147134506	2	Olfactory Bulb_3rd,Prefrontal Cortex_3rd
