# Supplementary material for: A Brain Region-Specific Predictive Gene Map for Autism Derived by Profiling a Reference Gene Set
Source: PLoS One. 2011 Dec 9;6(12):e28431. doi: 10.1371/journal.pone.0028431 (PMC3235126; doi:10.1371/journal.pone.0028431)
Supplement: Table S10 — Previously identified ASD-linked genes matching the AutRef84 dual profile which were not included in the input dataset. (PDF) [file pone.0028431.s012.pdf]

Supplementary Table S10. Previously identified ASD-linked genes matching the AutRef84 dual profile which were not included in the input dataset.

Gene	Relationship to ASD
ARNT2	Association
CADPS2	Functional
CCDC64	Association
CDH22	Association
CXCR3	Rare
GABRB1	Association
GABRB3	Association
GRIK2	Association
GRIN2A	Association
ITGB3	Association
NPAS2	Association
NRCAM	Association
NRP2	Association
RELN	Association
ROBO1	Functional
SEMA5A	Functional
SLC9A6	Syndromic
WNT2	Association
