# Supplementary material for: A Brain Region-Specific Predictive Gene Map for Autism Derived by Profiling a Reference Gene Set
Source: PLoS One. 2011 Dec 9;6(12):e28431. doi: 10.1371/journal.pone.0028431 (PMC3235126; doi:10.1371/journal.pone.0028431)
Supplement: Methods S1 — Bioconductor Statistics and Packages for GO Enrichment Analysis. (DOCX) [file pone.0028431.s013.docx]

**Methods S1. Bioconductor Statistics and Packages for GO Enrichment Analysis**

**A. Overall Statistics of Gene Ontology (GO) Enrichment Analysis**

*Summary of HyperGTest for Biological Process (BP):*

Gene to GO BP Conditional test for over-representation

1201 GO BP ids tested (20 have p < 0.001)

Selected gene set size: 71

Gene universe size: 12992

Annotation package: hgu133plus2

*Summary of HyperGTest for Molecular Function (MF):*

Gene to GO MF Conditional test for over-representation

271 GO MF ids tested (5 have p < 0.001)

Selected gene set size: 71

Gene universe size: 13703

Annotation package: hgu133plus2

*Summary of HyperGTest for Cellular Component:*

Gene to GO CC Conditional test for over-representation

156 GO CC ids tested (6 have p < 0.001)

Selected gene set size: 77

Gene universe size: 14249

Annotation package: hgu133plus2

**B. Packages for GO Analysis**

R version 2.6.1 (2007-11-26)

i386-pc-mingw32

locale:

LC_COLLATE=English_United

States.1252;LC_CTYPE=English_United

States.1252;LC_MONETARY=English_United

States.1252;LC_NUMERIC=C;LC_TIME=English_United States.1252

attached base packages:

[1] splines tools stats graphics grDevices utils

datasets

[8] methods base

other attached packages:

[1] Rgraphviz_1.16.0 GOstats_2.4.0 RBGL_1.14.0

[4] GO.db_2.0.2 hgu133a_2.0.1 KEGG_2.0.1

[7] GO_2.0.1 RColorBrewer_1.0-2 Category_2.4.0

[10] genefilter_1.16.0 survival_2.34-1 graph_1.17.15

[13] annotate_1.16.1 xtable_1.5-2

AnnotationDbi_1.0.6

[16] RSQLite_0.6-8 DBI_0.2-4 Biobase_1.16.3
